# p53 transcriptionally activates DCP1B to suppress tumor progression and enhance tumor sensitivity to PI3K blockade in non-small cell lung cancer

**DOI:** 10.1038/s41418-025-01501-y

**Published:** 2025-04-09

**Authors:** Shiqi Chen, Qian Hao, Yu Gan, Jing Tong, Chen Xiong, Quan Liao, Yang Zhang, Ting Ye, Xiang Zhou, Haiquan Chen

**Affiliations:** 1 Department of Thoracic Surgery and State Key Laboratory of Genetic Engineering, Fudan University Shanghai Cancer Center, Shanghai, 200032 China; 2 Institute of Thoracic Oncology, Fudan University, Shanghai, 200032 China; 3 Department of Oncology, Shanghai Medical College, Fudan University, Shanghai, 200032 China; 4 Fudan University Shanghai Cancer Center and Institutes of Biomedical Sciences, Fudan University, Shanghai, 200032 China; 5 Department of Radiation Oncology, Jiangxi Cancer Hospital, Jiangxi, 330029 China

**Keywords:** Non-small-cell lung cancer, Enzyme mechanisms

## Abstract

Non-small cell lung cancer (NSCLC), which accounts for approximately 85% of lung cancer patients, is characterized by its aggressive nature and poor prognosis. In this study, we identify decapping mRNA 1B (*DCP1B*) as a tumor suppressor gene that is transcriptionally regulated by p53. DCP1B is found to inhibit the growth and migration of NSCLC cells. Consistently, the level of DCP1B expression is decreased in NSCLC tissues, and its low expression is associated with NSCLC patients’ unfavorable outcomes. Mechanistic investigations reveal that DCP1B promotes the turnover of mitogen-activated protein kinase 4 (*MAPK4*) mRNA, and the activation of p53 reduces the expression level of MAPK4 partially through DCP1B. Notably, overexpression of MAPK4 can drive AKT phosphorylation independent of phosphoinositide 3-kinase (PI3K), thus neutralizing the anti-tumor activity of the PI3K inhibitor in NSCLC cells. Moreover, the p53 agonist combined with the PI3K inhibitor can suppress NSCLC proliferation synergistically in vitro and in vivo. Collectively, this study not only uncovers the function and mechanism of the p53-DCP1B-MAPK4 axis in suppressing NSCLC progression but also suggests a promising combination strategy for treating NSCLC.

## Introduction

Non-small cell lung cancer (NSCLC) is the primary subtype of lung cancer, accounting for the leading cause of cancer-related mortality [1]. Currently, NSCLC patients with specific genetic mutations, such as epidermal growth factor receptor (*EGFR*) E19 deletion/E21 L858R/E20 insertion mutation, Kirsten rat sarcoma (*KRAS*) G12C mutation, and others, have benefited from relevant targeted therapy [2–6]. However, resistance to these therapies is inevitable for NSCLC patients with disease development, thus leading to death eventually [7–9]. Therefore, it is of great significance to elucidate the molecular mechanism of NSCLC development and find new treatment strategies to improve the prognosis of NSCLC patients.

*TP53* is one of the most critical tumor suppressor genes in tumors, which plays a vital role in inhibiting tumorigenesis and tumor progression [10, 11]. p53 can protect cells from oncogenesis stress by regulating apoptosis, DNA damage repair, metabolism, and immune microenvironment, partially depending on its role as a transcription factor [12, 13]. Given the important role of p53 in tumorigenesis and tumor development, restoring the tumor suppressor function of p53 has been an essential strategy in developing p53 drugs for decades [14, 15]. However, most of these efforts failed. Very few p53 drugs have reached advanced clinical trial phases, and none have made it to National Medical Products Administration (NMPA) or Food and Drug Administration (FDA) approval for clinical use so far [14, 16]. Accordingly, there is an urgent need to elaborate on the new molecular mechanism of p53 in cancer to provide a fresh perspective for clinical trials of p53-related drugs.

The 5’ cap structure of mRNA plays a key role in mRNA stability and translation by protecting it from exonuclease degradation. Previous studies have shown that DCP1B is involved in mRNA degradation by removing the 5’ cap from mRNAs, a crucial step in regulated mRNA decay [17]. Usually, RNA capping enzymes are often highly expressed in tumors to support the high proliferation needs of tumor cells [18]. On the contrary, RNA decapping enzymes are crucial for RNA degradation and help maintain mRNA homeostasis [19]. Liu et al. reported that MOV10 can recruit the decapping enzyme DCP2 to mediate *LINE1* RNA decapping and facilitate the degradation of *LINE1* RNA [20]. In addition, several studies revealed the role of RNA decapping enzymes DCP1A and DCP2 in tumors and their association with the prognosis of cancer patients [21–26]. We identify that decapping mRNA 1B (*DCP1B*) as a transcriptional target of p53 in NSCLC cells. However, the function and underlying mechanisms of DCP1B in NSCLC remain unclear.

In this study, we found that DCP1B has decreased expression in NSCLC tumors, which is associated with an unfavorable prognosis in NSCLC patients. Mechanistic investigations revealed that p53 directly binds to the promoter of *DCP1B* and enhances its transcription. Additionally, DCP1B can facilitate the degradation of mitogen-activated protein kinase 4 (*MAPK4*), consequently leading to the inhibition of AKT activation independently of the PI3K pathway. Our results further demonstrated that knocking down DCP1B partially mitigates the tumor-inhibitory effect of p53 by increasing MAPK4 expression, and overexpression of MAPK4 stimulates the proliferation and migration of NSCLC cells partially by counteracting the effect of DCP1B. In summary, our study elucidates the function and mechanism of the p53-DCP1B-MAPK4 axis in suppressing NSCLC progression and increasing tumor sensitivity to PI3K blockade. Our findings also suggest that a combination of a p53 agonist and a PI3K inhibitor could significantly hinder the proliferation of NSCLC, offering a potential strategy for NSCLC treatment.

## Materials and methods

### Reagents, antibodies, and plasmids

Nutlin-3, 5-fluorouracil (5-Fu), Cisplatin, Etoposide, APG-115, Alpelisib, Actinomycin D (Act.D), and Dimethyl sulfoxide (DMSO) were purchased from MedChemExpress (MCE, Shanghai, China). Other reagents and kits, such as Cell Counting Kit-8, KOD-Plus-Mutagenesis Kit, and so on, were purchased from different companies, as detailed in Table S1. The antibodies against p21 (Catalog: 2947), DCP1B (Catalog: 13233), p-AKT (Catalog: 13038), AKT (Catalog: 9272), p-GSK3β (Catalog: 9336), and GSK3β (Catalog: 9315) were purchased from Cell Signaling Technology (CST). Other antibodies used included p53 (Santa Cruz, Catalog: sc-126), MAPK4 (Abcepta: Catalog: AP7298b), GAPDH (Proteintech, Catalog: 60004-1-Ig), Flag (Sigma-Aldrich, Catalog: F1804), HRP-conjugated affiniPure goat anti-rabbit IgG (Proteintech, Catalog: No. SA00001-2) and anti-mouse IgG (Proteintech, Catalog: No. SA00001-1) (Table S2). LentiCRISPRv2 (Addgene plasmid 52961) was purchased from Addgene (Cambridge, MA, USA). The gRNA sequences for p53 (Table S3) were designed on the website (http://www.e-crisp.org/E-CRISP/). The construction of sgp53 lentiviral vectors and Flag-p53 plasmid had been described previously. The plasmids encoding Flag-DCP1B and Flag-MAPK4 were purchased from Vigene Biosciences (Shandong, China). The cDNAs of *DCP1B* and *MAPK4* were subcloned into the vectors of Flag-pcDNA3.1 and Flag-pCDH, respectively, using the corresponding primers (Table S3). The specific sequences of shRNA targeting DCP1B and MAPK4 were obtained from Sigma-Aldrich Advanced Genomics and then cloned into the pLKO.1 vector (Table S3).

### Cell culture, lentiviral infection, and pcDNA3.1 plasmids and siRNA transfection

The human NSCLC cell lines A549, H460, H1395, H1299, Calu-1, PC-9, and H1975 and human embryonic kidney 293T (HEK293T) cell line were obtained from Cell Bank, Chinese Academy of Sciences (Shanghai, China) and Fudan University Shanghai Cancer Center (FUSCC). H1299, H1395, H1975, and H460 were cultured and maintained in RPMI medium with 10% fetal bovine serum and 1% penicillin–streptomycin. A549, Calu-1, PC-9, 293T were cultured and maintained in DMEM medium with 10% fetal bovine serum and 1% penicillin–streptomycin. All cell lines were cultured in a sterile incubator, which was set at 37 °C with 5% CO_2_. The sgRNA, PCDH, and shRNA plasmids, along with the packaging plasmids, psPAX2 and pMD2.G, were transfected into HEK293T cells, respectively. The lentivirus was collected 24–48 h after transfection and then used for infection. pcDNA3.1 plasmids and siRNAs were transfected into cells following the corresponding protocols. The siRNA obtained from GenePharma (Shanghai, China) sequences were listed in Table S4.

### RNA-sequencing

A549 cells were transfected with sgNC and sgp53 lentivirus and then were selected with p53 knockout to expand; those A549 cells with p53 knockout and the corresponding control cells were collected, total RNA was isolated using RNAiso Plus following the manufacturer’s protocol (Takara, Japan), and RNA-sequencing was provided by OEbiotech (shanghai, China). A549 cells transfected with siNC and siDCP1B for 48 h were collected, total RNA was isolated using RNAiso Plus following the manufacturer’s protocol (Takara, Japan), and RNA-sequencing was provided by OEbiotech (shanghai, China). The raw data generated have been deposited in the GSA human data repository respectively with the accession numbers HRA010596 and HRA010599.

### RT-qPCR and western blot

Total RNA was isolated using RNAiso Plus (Takara, Japan). Complementary DNAs (cDNAs) were synthesized from 0.2 to 0.5 μg RNA using Hiscript III qRT SuperMix (Vazyme). Quantitative PCR (qPCR) was conducted using SYBR qPCR Master Mix according to the manufacturer’s protocol (Vazyme). The relative expression levels of mRNAs were calculated using the comparative Ct method normalized to GAPDH. The primers for Quantitative PCR (qPCR) are listed in Table S5. Cell lysates were prepared in RIPA buffer, and protein concentrations were quantified using a Pierce BCA protein assay kit. An equal amount of protein (20–40 μg) was used in western blot analysis.

### Dual-luciferase reporter assay

The *DCP1B* promoter region was amplified from the genomic DNA of A549 cells and cloned into the pGL3-basic vector. Cells were seeded in 24-well plates and co-transfected with the plasmids encoding p53, p53-RE-WT or p53-RE-Mut, and Renilla luciferase. Luciferase activity was measured 48 h post-transfection according to the manufacturer’s instructions using a dual-luciferase reporter system (Promega, Madison, WI, USA).

### Chromatin immunoprecipitation assay

Chromatin immunoprecipitation (ChIP) was conducted using Magna ChIP A/G Chromatin Immunoprecipitation Reagent (Merck, Darmstadt, Germany). Briefly, cells were crosslinked with 1% formaldehyde and then terminated with glycine. Cells were lysed in ChIP lysis buffer supplemented with protease inhibitors. Chromatins were then sheared by sonication. The anti-p53 antibody or IgG, together with magnetic beads, was added to the chromatin solution, rotating at 4 °C overnight. The bound DNAs were purified and assessed by PCR. The primers for ChIP analysis are listed in Table S5.

### Cell proliferation, colony formation, and cell migration assays

For cell proliferation assays, cells were plated at a density of 1500–2000 cells per well on 96-well plates with five replicates 6–12 h after transfection. For drug-sensitivity and the combination index (CI) of 2 drugs assays, cells (4000 cells per well) were seeded in 96-well plates with five replicates overnight and then the cells of drug-sensitivity assays were treated with or without the indicated concentrations of drugs (IC_50_) for 0 h, 24 h, 47 h, 72 h, and 96 h respectively and the combination index (CI) of 2 drugs assays were treated with or without the indicated concentrations of drugs (Serial dilution) for 72 h. The Cell Counting Kit-8 (CCK-8, #40203ES92, Yeasen) was used for the cell viability assay according to the manufacturer’s instructions, and the absorbance at 450 nm (A450) was measured at the indicated times. For colony formation assays, cells were seeded into 6-well plates (800-1000 cells per well) and then cultured for 2 weeks. The colonies were fixed with methanol and stained with 0.2% crystal violet. Colonies were counted using the ImageJ software. For cell migration assays, 5–8 × 10^4^ cells were resuspended in a serum-free medium and then plated in the upper chamber (#353097, Corning), and a medium containing 20% FBS (700 mL) was added to the lower chamber. After incubation for 16 to 24 h, the cells in the upper chamber were fixed with methanol and stained with 0.2% crystal violet.

### Clinical samples

RNA-sequencing and whole-exome sequencing (WES) data of LUAD patients (*N* = 197) were obtained from our previous study [27]. NSCLC specimens (*N* = 408) were collected after surgery in FUSCC, and tissue microarrays (TMA) were produced by WEIAO BioTech (Shanghai, China) and then subjected to immunohistochemistry (IHC) analysis. The location of each specimen on the TMA and the clinicopathological data, including age, gender, tumor size, prognosis, etc., were recorded in detail. This research involving human data and human tissue was performed based on the Declaration of Helsinki and approved by the Ethics Committee of FUSCC, and written informed consent was obtained from all patients before surgery (reference number: 1612167-18).

### Immunohistochemistry

TMA were processed through deparaffinization, rehydration, and antigen retrieval. After overnight incubation with anti-DCP1B antibody at 4 °C, slides were treated with HRP-conjugated secondary antibody and DAB substrate following PBS washes, with hematoxylin counterstaining. DCP1B expression quantification was independently assessed by two blinded investigators using ImageScope software.

### RNA immunoprecipitation

H460 cells were harvested and suspended in RIP buffer (10 mM Tris, 150 mM NaCl, 1 mM Na_2_EDTA.2H_2_O, 3.5 mM SDS, 1 mM DTT, 1%NP-40, pH7.4). The cell lysate was then immunoprecipitated with anti-DCP1B magnetic beads (Cat. No. B26101, bimake, Shanghai, China) overnight at 4 °C. The beads were washed six times with RIP buffer, followed by RNA purification and RT-qPCR analysis. The primers for RIP-qPCR are listed in Table S5.

### RNA stability assay

To determine whether DCP1B overexpression affects the stability of *MAPK4* mRNA in H460 cells, we treated cells with 5 μM actinomycin D (Cat. No. HY-17559, MedChemExpress) at different time points as indicated. Cells were then harvested for RNA isolation and RT-qPCR analysis.

### Xenograft mouse models and treatment

Female BALB/c nude mice at 4–5 weeks old were used in the xenograft studies purchased from the Department of Laboratory Animal Center of FUSCC. A total of 5 × 10^6^ A549 cells with DCP1B overexpression or DCP1B downregulation and the corresponding blank control cells were injected into the subcutaneous. The combination effects of APG-115 with Alpelisib were tested in xenograft mouse models. BALB/c nude mice were injected with A549 cells subcutaneously and randomly divided into four groups for different treatments, including vehicle, APG-115, Alpelisib, and a combination of both. APG-115 (80 mg/kg) or Alpelisib (30 mg/kg) in a volume of 100 μl was administered intraperitoneally five times a week. Tumor growth was monitored with electronic digital calipers in two dimensions. Tumor volume was calculated according to the formula: volume = length × width × width × 0.52. Mice were killed by euthanasia, and tumors were harvested for future analyses. The animal protocols were in accordance with ethical guidelines and approved by the Animal Welfare Committee of FUSCC (reference number: FUSCC-IACUC-2022169, FUSCC-IACUC-2022260 and FUSCC-IACUC-2022327).

### Statistical analysis

All in vitro experiments were performed in biological triplicate. *p* values were obtained by two tailed *t*-test or analysis of variance (ANOVA) using GraphPad Prism 5.0. The Kaplan–Meier method was used to analyze the significant difference in patient survival. *p* < 0.05 was considered statistically significant. Asterisks denote statistical significance: *p < 0.05; **p < 0.01; ***p < 0.001.

## Results

### p53 transcriptionally activates DCP1B

**Fig. 1:**
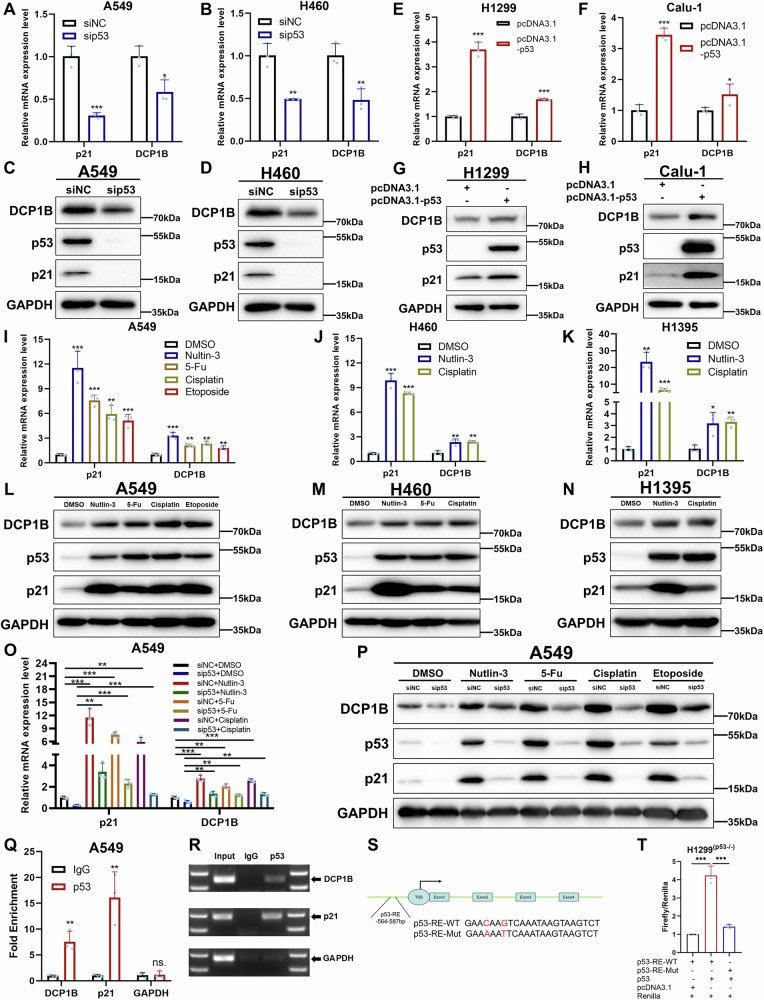
p53 transcriptionally activates DCP1B. **A**–**D** RT-qPCR and WB assays validating the expression levels of DCP1B in A549 and H460 cells transfected with siNC and sip53. **E**–**H** RT-qPCR and WB assays validating the expression levels of DCP1B in H1299 and Calu-1 cells transfected with Flag-NC and Flag-p53. **I**–**N** RT-qPCR and WB assays validating the expression levels of DCP1B in A549, H460, and H1395 cells treated with p53-inducing agents including Nutlin-3, Cisplatin, 5-Fluorouracil (5-FU), and Etoposide for 24 h. **O**, **P** After transfection with siNC and sip53 for 48 h, A549 cells were treated with DMSO and p53-inducing agents including Nutlin-3, Cisplatin, 5-Fluorouracil (5-FU), and Etoposide for 24 h and then subjected to RT-qPCR and WB assays validating the expression levels of DCP1B. **Q**, **R** CHIP-qPCR assay showing the bound *DCP1B* DNA elements. **S**, **T** Dual-luciferase reporter gene assay showing p53-responsive *DCP1B* DNA elements. Data in **A**, **B**, **E**, **F**, **I**–**K**, **O**, **Q** and **T** are represented as mean ± SD, *n* = 3.

In this study, by analyzing the RNA-sequencing data of A549 NSCLC cells with and without the knockout of p53 by CRISPR/Cas9, we confirmed that *DCP1B* was one of the p53-target genes (Fig. S1A–E). To validate this finding, we knocked down p53 by siRNA and found that the expression of DCP1B was significantly reduced at RNA and protein levels using RT-qPCR and Western Blot (WB) analyses in A549 and H460 NSCLC cells (Fig. 1A–D). Subsequently, we overexpressed p53 in H1299 and Calu-1 NSCLC cells and observed that the expression of DCP1B was markedly induced at mRNA and protein levels (Fig. 1E–H). Additionally, we activated p53 with different p53-inducing agents, including Nutlin-3, 5-FU, cisplatin, and etoposide in various NSCLC cells, and found that the expression of DCP1B was upregulated in p53-wide type NSCLC cells (A549, H460, H1395) (Fig. 1I–N) but not in p53-deficiency NSCLC cells (H1299, Calu-1) (Fig. S1F–I) and p53-mutation NSCLC cells (H1975, PC9) (Fig. S1J–Q). Furthermore, ablation of p53 by siRNA dramatically prevented the upregulation of DCP1B caused by p53-inducing agents (Fig. 1O, P). These results suggested that *DCP1B* might be transcriptionally regulated by p53. Therefore, chromatin immunoprecipitation (ChIP) qPCR assay was conducted and revealed that p53 can bind to the promoter region of *DCP1B* in A549 cells (Fig. 1Q, R). These results were consistent with a previous study reporting a potential p53 binding site on DCP1B promoter using the ChIP-on-chip approach [28]. Dual-luciferase reporter gene experiment demonstrated that overexpression of p53 can induce the luciferase activity driven by the promoter region of *DCP1B* (Fig. 1S, T). Together, these results demonstrate that *DCP1B* is a transcriptional target gene of p53.

### DCP1B acts as a tumor suppressor to inhibit NSCLC proliferation and migration

**Fig. 2:**
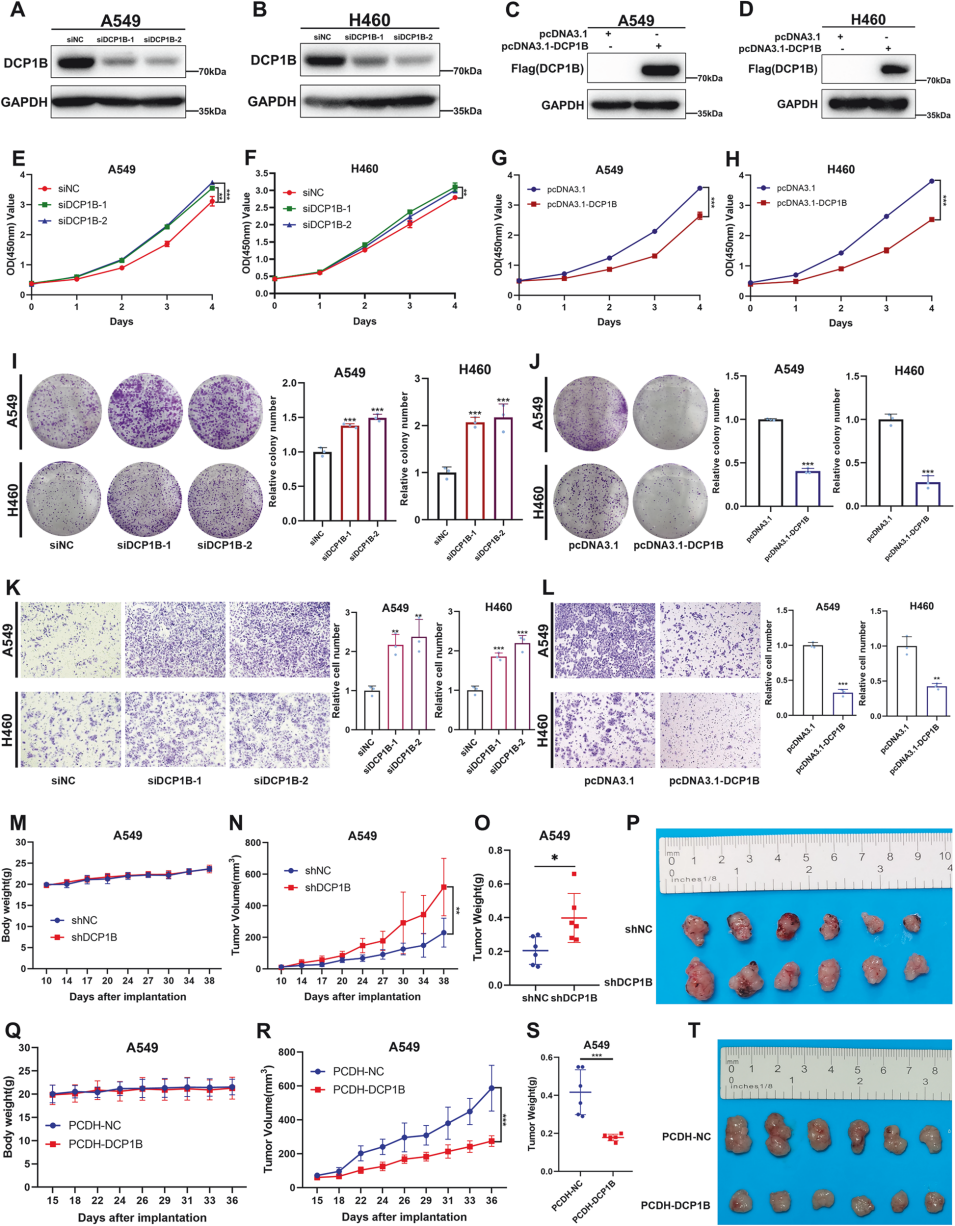
DCP1B acts as a tumor suppressor to inhibit NSCLC proliferation and migration. **A**–**D** Expression validation of DCP1B in A549 and H460 cells transfected with siNC and siDCP1B (#1, #2) or Flag-NC and Flag-DCP1B plasmids by WB. **E**–**L** A549 and H460 cells transfected with siNC and siDCP1B (#1, #2) or Flag-NC and Flag-DCP1B plasmids were then subjected to cell proliferation, colony formation, and cell migration assays, representative images and corresponding quantitative results of colony formation and cell migration assays are shown. Data are represented as mean ± SD, *n* = 3. **M**–**T** A549 cells stably expressing shNC and shDCP1B or pCDH-NC and pCDH-DCP1B were inoculated into BALB/c female nude mice. After 36–38 days of injection, mice were sacrificed and xenograft tumors were removed. Representative mice weight, tumor volume, tumor weight, and tumor images are shown. Data in **N**, **O**, **R** and **S** are represented as mean ± SD, *n* = 6.

Subsequently, we explored the biological functions of DCP1B in NSCLC cells. We knocked down or overexpressed DCP1B in wild-type p53 cells (A549, H460) and mutant p53 cells (H1299, H1975) respectively, and the expression levels of DCP1B in corresponding cell lines was validated by WB (Figs. 2A–D and S2A–D). CCK-8 and colony formation assays demonstrated that knockdown of DCP1B increased cell proliferation and colony formation, whereas opposite results were obtained following ectopic expression of DCP1B in these cells (Figs. 2E–J and S2E–J). Cell migration assay indicated that DCP1B knockdown promoted the migratory potential of NSCLC cells, whereas overexpression of DCP1B suppressed the migration of these cells (Figs. 2K, L and S2K, L). In addition, we established stable A549 cell lines that overexpress DCP1B shRNA or the DCP1B-encoding plasmid. These engineered cells were subcutaneously injected into BALB/c-nude mice. Our results showed that DCP1B deficiency significantly increased the xenograft tumor growth rate, resulting in larger tumor weight and size (Fig. 2M–P). On the contrary, DCP1B overexpression significantly inhibited the growth of xenograft tumors (Fig. 2Q–T). Taken together, these results demonstrate that DCP1B serves as a tumor suppressor in a p53-independent manner that inhibits the progression of NSCLC cells both in vitro and in vivo.

### Low levels of DCP1B are associated with an unfavorable prognosis in NSCLC patients

**Fig. 3:**
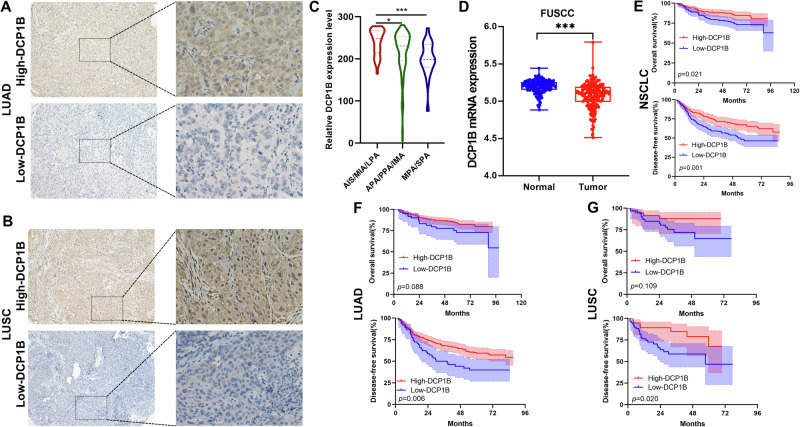
Low levels of DCP1B are associated with an unfavorable prognosis in NSCLC patients. **A**, **B** Immunohistochemical staining showing the representative samples with low and high expression of DCP1B in LUAD and LUSC patients from FUSCC TMA. **C**, **D** mRNA levels of *DCP1B* in LUAD tissues and adjacent normal tissues in the FUSCC RNA-seq database. **E**–**G** Kaplan–Meier analysis of the OS and DFS of NSCLC patients with high and low DCP1B expression.

Since DCP1B played an important role in suppressing the progression of NSCLC both in vitro and in vivo, we investigated the clinical significance of DCP1B using the databases of FUSCC. We performed immunohistochemistry (IHC) staining analysis of DCP1B expression using the microarrays of NSCLC (Fig. 3A, B) and found that the protein expression level of DCP1B was significantly higher in the lung adenocarcinoma (LUAD) subgroup of adenocarcinoma in situ (AIS)/minimally invasive adenocarcinoma (MIA)/lepidic predominant adenocarcinoma (LPA) than in the LUAD subgroups of acinar predominant adenocarcinoma (APA)/papillary predominant adenocarcinoma (PPA)/invasive mucinous adenocarcinoma (IMA) and micropapillary predominant adenocarcinoma (MPA)/solid predominant adenocarcinoma (SPA) (*p* < 0.001) (Table S6) and it decreased gradually with the increasing tumor grade (Fig. 3C), and the high expression of DCP1B was significantly associated with NSCLC patients with smaller tumor size (*p* = 0.034) (Table S6). Furthermore, our RNA-sequencing data of LUAD demonstrated that the mRNA expression level of *DCP1B* was decreased in LUAD tissues compared to normal tissues (Fig. 3D), and *DCP1B* was expressed at lower levels in *TP53*^Mut^ tumors than in *TP53*^WT^ tumors (Fig. S3A). Next, Kaplan–Meier survival analysis was conducted and showed that higher expression of DCP1B was associated with favorable overall survival (OS) and disease-free survival (DFS) of NSCLC patients (Fig. 3E). Subgroup analysis showed that patients with higher expression of DCP1B had a better prognosis in both LUAD and lung squamous cell carcinoma (LUSC) cohorts (Fig. 3F, G). In addition, higher expression of DCP1B correlated with better overall survival in *TP53*^WT^ patients, whereas its prognostic value was insignificant in *TP53*^Mut^ patients (Fig. S3B, C). Collectively, these results suggest that DCP1B is less expressed in NSCLC tissues, which predicts poor prognosis of NSCLC patients.

### p53 promotes the degradation of *MAPK4* mRNA partially through DCP1B

**Fig. 4:**
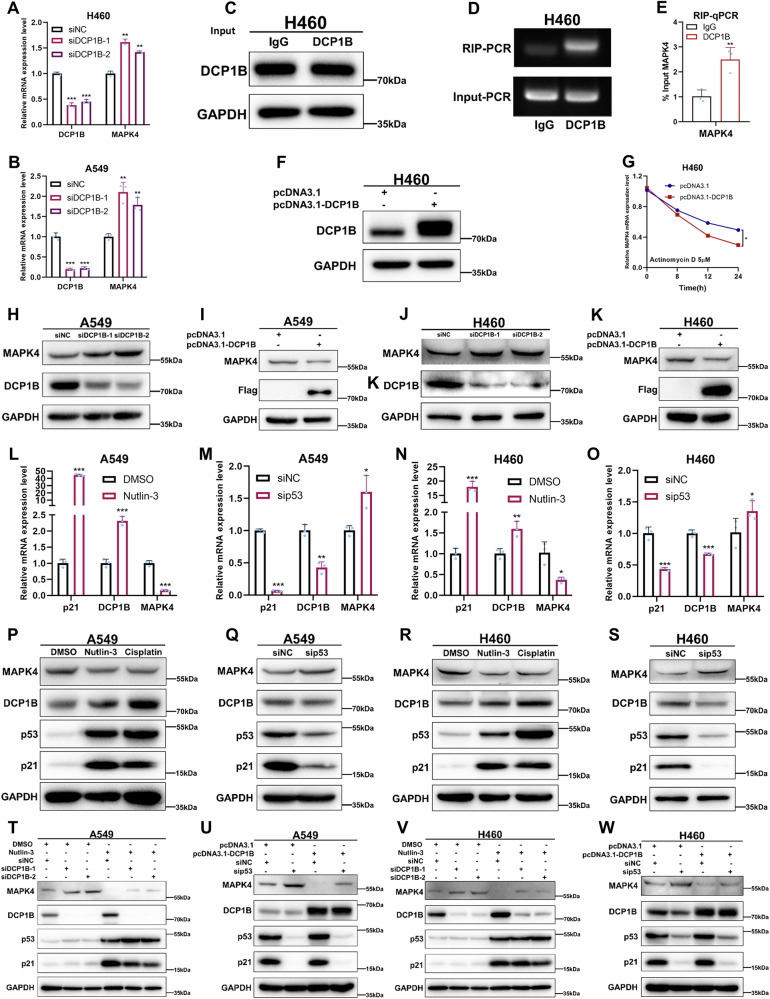
p53 promotes the degradation of MAPK4 mRNA partially through DCP1B. **A**, **B** RT-qPCR validating the expression levels of MAPK4 in A549 and H460 cells transfected with siNC and siDCP1B (#1, #2) according to the RNA-seq data. **C**–**G** RIP assay showing the interaction between DCP1B and *MAPK4* mRNA in H460 cells. RT-qPCR analysis assessing the RNA stability in H460 cells transfected with control or Flag-DCP1B treating with Act. D (5 μM) on the indicated time. **H**–**K** WB assay validating the expression levels of MAPK4 in A549 and H460 cells transfected with siNC and siDCP1B (#1, #2) or Flag-NC and Flag-DCP1B. **L**–**S** RT-qPCR and WB assays assessing the expression levels of MAPK4 in A549 and H460 treated with p53-inducing agents including Nutlin-3 and Cisplatin for 24 h or siNC and sip53. **T**–**W** WB assays demonstrating the expression levels of MAPK4 in A549 and H460 cells treating with siNC and siDCP1B (#1, #2) in combination with or without p53-inducing agent Nutlin-3, or transfected with siNC and sip53 alone in combination with or without Flag-DCP1B. Data in **A**, **B**, **E**, **G** and **L**–**O** are represented as mean ± SD, *n* = 3.

To investigate the underlying mechanism of DCP1B’s suppressive function in NSCLC, RNA sequencing was performed to reveal the potential downstream of DCP1B in A549 cells. Our results showed that 44 genes were downregulated and 34 genes were upregulated when excluding the genes with low expression levels after DCP1B knockdown (Fig. S4A, B). Through RT-qPCR analysis, *MAPK4* was confirmed to be a crucial gene regulated by DCP1B, and ablation of DCP1B markedly promoted *MAPK4* expression at the mRNA level in NSCLC cells (Fig. 4A, B). Since DCP1B possesses RNA-binding ability, we tested whether DCP1B could bind to *MAPK4* mRNA and regulate its stability by conducting RIP and RNA stability assays. RIP assay demonstrated that DCP1B can bind to *MAPK4* mRNAs (Fig. 4C–E). RNA stability assay identified that DCP1B overexpression significantly reduced *MAPK4* mRNA levels when DNA transcription was blocked by 5 μM actinomycin D (Act. D), indicating that DCP1B could regulate *MAPK4* mRNA stability (Fig. 4F, G). Consistently, knockdown of DCP1B increased the protein levels of MAPK4, while overexpression of DCP1B reduced MAPK4 protein levels (Fig. 4H–K). Furthermore, we tested whether p53 regulates the expression of MAPK4 and, if so, whether DCP1B is involved in this regulation. Our results showed that activation of p53 could inhibit the expression of MAPK4 at both mRNA and protein levels, whereas p53 depletion could elevate the expression of MAPK4 at both mRNA and protein levels (Fig. 4L–S), while these results were not observed in NSCLC cells that harbor deficiency p53 and mutant p53 (Fig. S4C–H). Remarkably, we found that knockdown of DCP1B partially reversed the decrease in MAPK4 levels in response to p53 activation, while overexpression of DCP1B partially reduced MAPK4 levels following p53 depletion (Fig. 4T–W). Collectively, these results demonstrate that p53 activation promoted the degradation of *MAPK4* mRNA partially via DCP1B.

### DCP1B suppresses NSCLC progression by inhibiting MAPK4

**Fig. 5:**
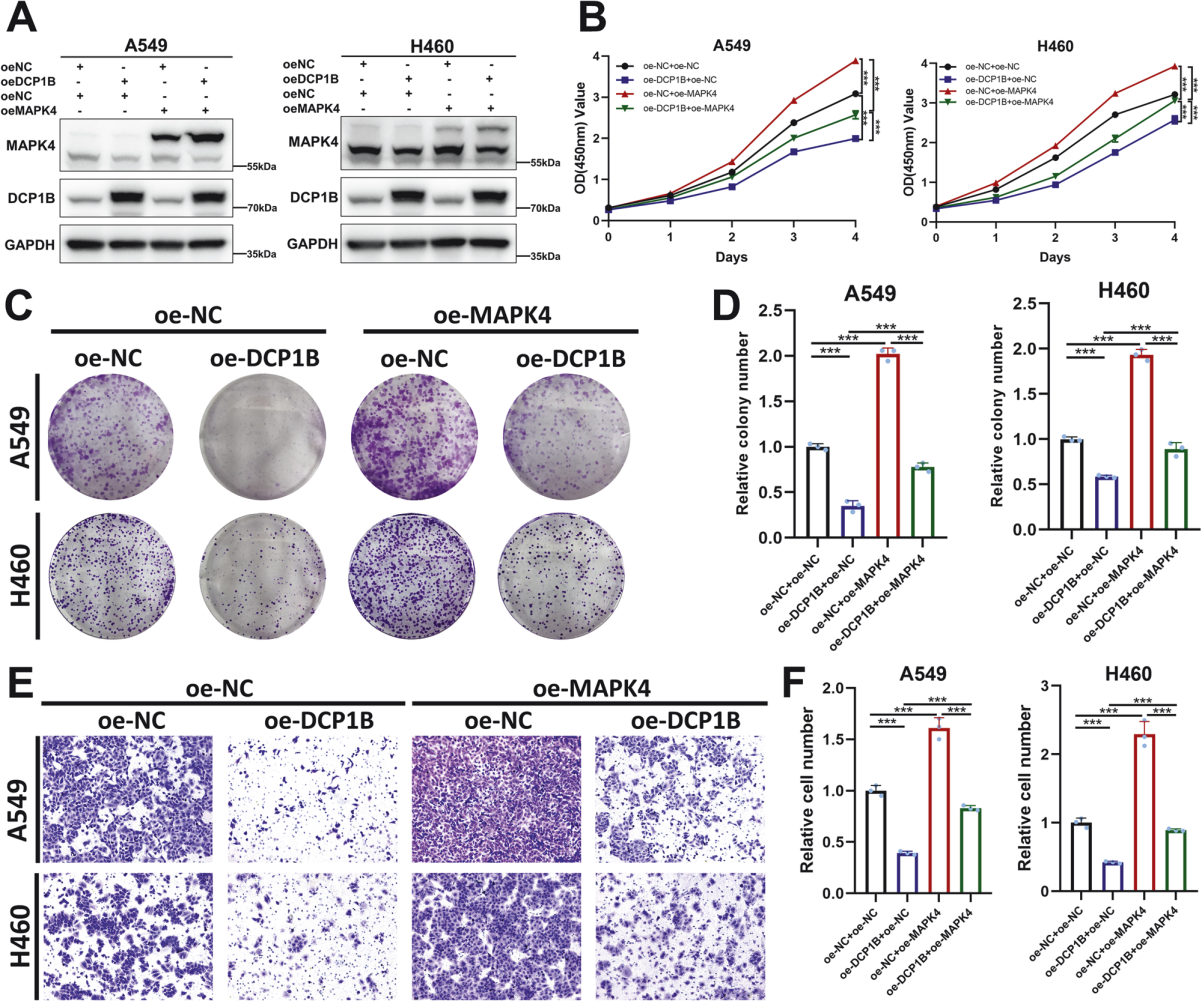
DCP1B suppresses NSCLC progression by inhibiting MAPK4. **A** WB assay showing the expression levels of DCP1B and MAPK4 in A549 and H460 cells transfected with Flag-DCP1B and Flag-MAPK4. **B**–**F** A549 and H460 cells transfected with Flag-DCP1B and Flag-MAPK4 plasmids were then subjected to cell proliferation, colony formation, and cell migration assays, representative images and corresponding quantitative results of colony formation and cell migration assays were shown. Data are represented as mean ± SD, *n* = 3.

*MAPK4* has been reported to be an oncogenic gene in triple-negative breast cancer (TNBC) and prostate cancer, and similar results were also found in NSCLC cells. We knocked down or overexpressed MAPK4 in A549 and H460 cells, respectively, and the expression levels of MAPK4 in corresponding cell lines was validated by WB (Fig. S5A, B, E, F). CCK-8 assay demonstrated that knockdown of MAPK4 reduced cell proliferation (Fig. S5C, D), whereas opposite results were obtained following ectopic expression of MAPK4 in these cells (Fig. S5G, H). Next, survival analysis was conducted in the Kaplan–Meier Plotter website and showed that increased expression of MAPK4 was associated with unfavorable overall survival (OS) and progression-free survival (PFS) of NSCLC patients (Fig. S5I, J). Subgroup analysis got the same results in both LUAD cohorts (Fig. S5K, L) and LUSC cohorts (Fig. S5M, N). Collectively, these results suggest that MAPK4 is an oncogenic factor in NSCLC. To explore whether DCP1B meditated NSCLC progression through MAPK4, we performed CCK-8, colony formation, and transwell assays by overexpressing both DCP1B- and MAPK4-encoding plasmids in different combinations (Fig. 5A). Our results showed that MAPK4 overexpression significantly impaired the inhibitory effect of DCP1B on cell proliferation and colony formation (Fig. 5B–D). Consistently, the ability of DCP1B to suppress the migration of NSCLC cells was partially attenuated by the overexpression of MAPK4 (Fig. 5E, F). Taken together, these results indicate that DCP1B suppresses NSCLC progression by reducing the levels of MAPK4.

### MAPK4 enhances AKT phosphorylation independent of PI3K in NSCLC

**Fig. 6:**
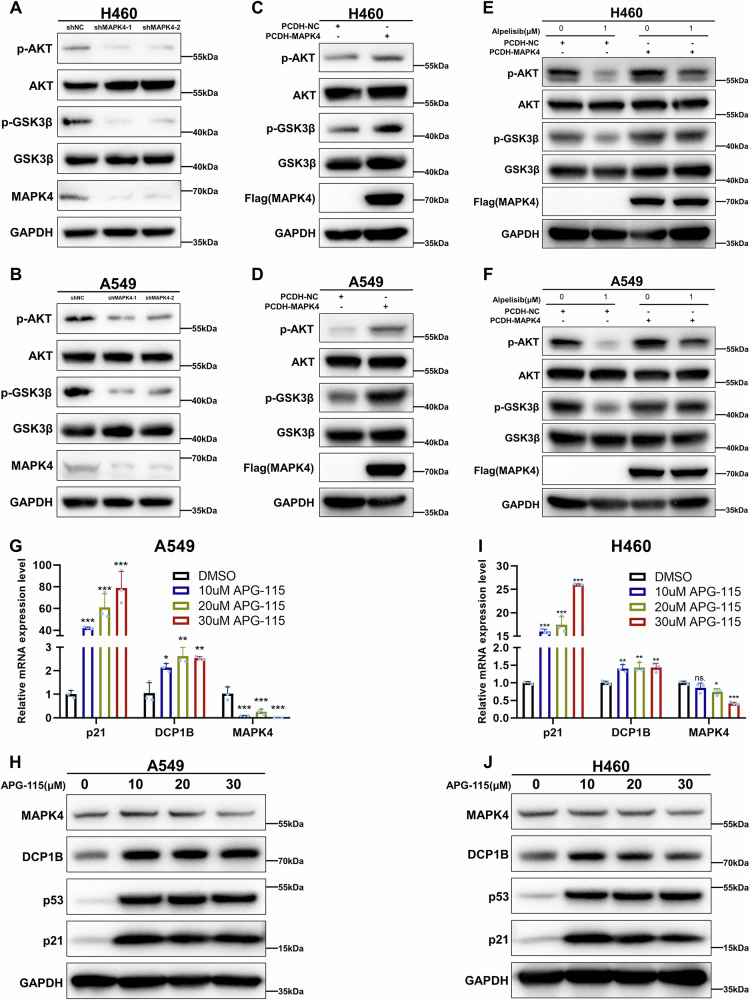
MAPK4 enhances AKT phosphorylation independent of PI3K in NSCLC. **A**, **B** WB assays assessing the expression levels of downstream proteins in A549 and H460 cells stably expressing shNC and shMAPK4. **C**, **D** WB assays assessing the expression levels of downstream proteins in A549 and H460 cells stably expressing PCDH and PCDH-MAPK4. **E**, **F** WB assays assessing the expression levels of downstream proteins in A549 and H460 cells stably expressing PCDH and PCDH-MAPK4 treated with or without PI3K inhibitor Alpelisib. **G**–**J** RT-Qpcr and WB assays validating the expression levels of MAPK4 in A549 and H460 treated with gradually increasing drug concentrations of p53-inducing agent APG-115 for 24 h. Data in **G** and **I** are represented as mean ± SD, *n* = 3.

According to previous studies, MAPK4 could bypass PI3K to activate AKT phosphorylation [29, 30]. Therefore, we further explored whether this result could be replicated in NSCLC cells. Our results showed that overexpression of MAPK4 could activate AKT phosphorylation, whereas knockdown of MAPK4 could inhibit AKT phosphorylation (Fig. 6A–D). Further experiments demonstrated that the overexpression of MAPK4 markedly counteracted the effect of the PI3K inhibitor Alpelisib, as evidenced by the partial restoration of the phosphorylation level of AKT (Fig. 6E, F). These results indicate that MAPK4 activates AKT, bypassing the PI3K pathway in NSCLC cells.

### The combination of a p53 agonist and a PI3K inhibitor effectively suppresses the growth of NSCLC

**Fig. 7:**
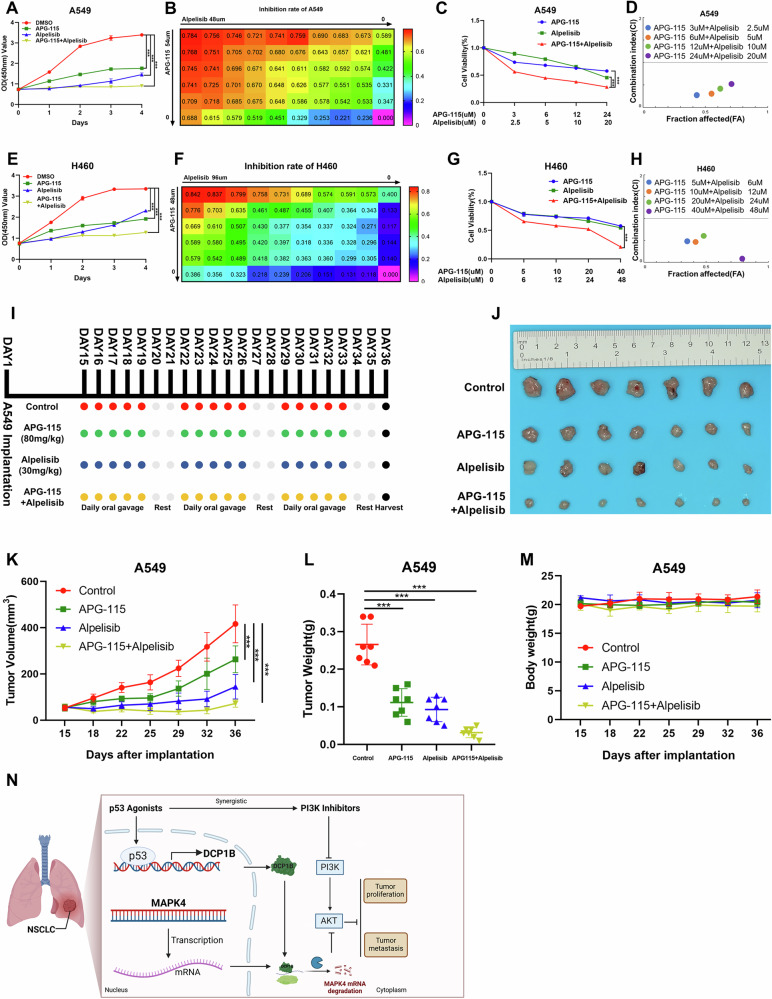
The combination of a p53 agonist and a PI3K inhibitor effectively suppresses the growth of NSCLC. **A**–**H** Drug-sensitivity and the combination index (CI) of 2 drugs assays showing the synergistic inhibition effect of p53 agonist and PI3K inhibitor in A549 and H460 cells in vitro. Data in **A**, **C**, **E** and **G** are represented as mean ± SD, *n* = 3. **I**–**M** A549 cells were inoculated into BALB/c female nude mice. After 14 days of injection, mice were randomly divided into four groups including the control group, APG-115 group, Alpelisib group, and the co-drug group. After 3 weeks, mice were sacrificed and xenograft tumors were removed. Representative mice weight, tumor volume, tumor weight, and tumor images are shown. **N** The proposed working model. Data in **K** and **L** are represented as mean ± SD, *n* = 7.

Since p53 inhibits the levels of MAPK4, we then investigated whether the combination of a p53 agonist and a PI3K inhibitor could synergistically suppress NSCLC. We employed the p53 agonist APG-115 in our study, as it is currently undergoing clinical trials for solid tumors with wild-type p53. We treated these NSCLC cells with increasing doses of APG-115. As expected, the expression of MAPK4 decreased gradually (Fig. 6G–J). Next, we tested the combined effect of APG-115 and Alpelisib in the treatment of NSCLC both in vitro and in vivo. First, we measured the combination index of APG-115 and Alpelisib and found that the two drugs present a synergistic activity to inhibit the proliferation of NSCLC (Fig. 7A–H). In addition, we tested the combination effect of APG-115 with Alpelisib in xenograft mouse models. BALB/c-nude mice were inoculated with A549 cells subcutaneously and randomly divided into four groups for different treatments, including vehicle, APG-115, Alpelisib, and the combination of APG-115 with Alpelisb. While the single-agent treatment significantly inhibited tumor growth in vivo, the combinational treatment suppressed the tumor growth to a greater extent, as evidenced by the tumor size, weight, and growth rate (Fig. 7I–L). The potential drug-related adverse effects were tolerable, as the average weight of the treated mice was similar to that of the control mice (Fig. 7M). These results demonstrate that the combination of APG-115 and Alpelisib can effectively suppress NSCLC in vitro and in vivo.

## Discussion

In this study, we have demonstrated that *DCP1B* is a tumor suppressor gene that can be transcriptionally activated by p53 in NSCLC cells. p53 activation increased the transcription of *DCP1B*, which in turn reduced the expression of MAPK4. Inhibition of MAPK4 downregulated the level of AKT phosphorylation independent of the PI3K pathway and improved tumor sensitivity to the PI3K inhibitor Alpelisib. Importantly, the p53 agonist APG-115 combined with Alpelisib could synergistically suppress NSCLC growth in vitro and in vivo, which provides a possible strategy for the treatment of NSCLC (Fig. 7N).

In human cancer, p53 activates the expression of a myriad of target genes as a transcription factor to regulate various biological processes [31]. In this study, we identified that *DCP1B* is a bona fide target gene of p53. DCP1B protein is localized in the cytoplasm, serving a crucial function in the degradation of mRNAs. It plays a significant role in both normal mRNA turnover and nonsense-mediated mRNA decay processes. Chen et al. recently conducted a significant investigation into the crucial roles of DCP1A and DCP1B in cancer and gene expression regulation [32]. They reported that the expression levels of DCP1A were reduced in various cancers including LUAD and LUSC, and the higher expression level of DCP1A was associated with a longer progression-free interval. In contrast, DCP1B exhibited distinct expression patterns across tumor types, with its prognostic implications varying depending on tumor subtypes. Notably, the expression pattern and prognostic implication of DCP1B in NSCLC remain unexplored. In our study, we initially found that DCP1B served as a tumor suppressor in NSCLC and high expression of DCP1B was associated with a favorable prognosis in NSCLC patients. In FUSCC cohorts, DCP1B is decreased in tumor specimens, and the lower expression of DCP1B is associated with a worse prognosis of NSCLC patients. Furthermore, we found that the expression of DCP1B was significantly higher in *TP53*^WT^ patients than that in *TP53*^Mut^ patients, as mutant p53s were unable to activate the expression of DCP1B. Interestingly, we could not observe a significant prognostic impact of DCP1B in *TP53*^Mut^ patients. This might be due to the following reasons. First, based on our previous studies [27, 33, 34], the presence of p53 mutations is frequently associated with tumor progression, including alterations in tumor heterogeneity, changes in tumor size, lymph node and distant metastasis, which may potentially obscure the prognostic impact of DCP1B. Second, a small sample size of patients in *TP53*^Mut^ cohort (*N* = 38) may also result in the insignificant prognostic value. Therefore, a larger sample size is needed for further analysis of the prognostic value of DCP1B in *TP53*^Mut^ NSCLC patients. Functionally, the ablation of DCP1B promotes, whereas the ectopic DCP1B suppresses, the progression of NSCLC regardless of whether p53 is mutated or not. Collectively, our study uncovers that DCP1B plays a p53-indepentdent tumor suppressive function in NSCLC for the first time.

To investigate the underlying mechanism by which DCP1B suppresses NSCLC, RNA sequencing was conducted after knocking down DCP1B in A549 cells, resulting in the identification of *MAPK4* as a key downstream gene that modulates AKT activity. Chen et al. reported that DCP1A and DCP1B served as the interacting proteins of DCP2 and increased the mRNA-binding affinity of DCP2, while we found that DCP1B could directly bind to the MAPK4 mRNA and affect its stability. Our results revealed that DCP1B promoted the turnover of *MAPK4* mRNA, and the expression level of DCP1B was inversely correlated with MAPK4 expression in NSCLC. Interestingly, the activation of p53 led to a decrease in the mRNA and protein levels of MAPK4 by activating DCP1B. Further experiments showed that ablation of DCP1B could partly reduce the impact of p53 activation on MAPK4 expression. Conversely, ectopic expression of DCP1B could partly counteract the effect of p53 depletion on MAPK4 expression. Additionally, overexpression of MAPK4 could enhance the proliferation of NSCLC cells and partially alleviate the inhibitory effect of DCP1B on NSCLC progression. Hence, our study unveils a novel role and provides mechanistic insights into the p53-DCP1B-MAPK4 axis in NSCLC.

As one of the mitogen-activated protein kinases, MAPK4 has been shown to play a role in promoting cancer progression in tumors. In 2019, Yang et al. reported that MAPK4 can bypass PI3K to activate AKT, and blocking MAPK4 can enhance the inhibitory effect of PI3K inhibitor on tumors [30]. The oncogenic role of MAPK4 in prostate cancer and triple-negative breast cancer was subsequently reported [29, 35]. In addition, Tian et al. reported that MAPK4 knockdown could promote the sensitivity of cervical cancer to radiotherapy and PARP1 inhibitors in 2020 [36]. Consistently, our results showed that overexpression of MAPK4 activates AKT, bypassing PI3K in NSCLC cells. Thus, these findings suggest that blocking MAPK4 may enhance the sensitivity of NSCLC to PI3K inhibitors.

Since p53 represses MAPK4 expression by activating DCP1B, we tested whether the p53 agonist APG-115 and the PI3K inhibitor Alpelisib have a synergistic effect in inhibiting the proliferation of NSCLC cells. In vitro experiments showed that the combination of APG-115 and Alpelisib can significantly inhibit the proliferation of NSCLC, and the two drugs have a synergistic effect. Subsequently, the combination of the two drugs was tested in vivo. The results showed that APG-115 combined with Alpelisib could significantly inhibit the growth of xenograft tumors and exhibited a more profound tumor-inhibitory effect than either APG-115 or Alpelisib used alone.

In conclusion, this study uncovers that *DCP1B* is a p53 target gene and serves as a tumor suppressor in NSCLC. Mechanistically, DCP1B facilitates the degradation of *MAPK4* mRNA, bypassing the PI3K pathway and leading to the inhibition of AKT activity. Also, our findings demonstrate that APG-115 and Alpelisib synergistically suppress NSCLC both in vitro and in vivo, suggesting a promising combination strategy for the treatment of NSCLC.

## Supplementary information

Supplementary Figures

Supplementary Tables

Uncropped WB

## Data Availability

The data generated in this study are available upon request from the corresponding author.

## References

[1] Bray F, Laversanne M, Sung H, Ferlay J, Siegel RL, Soerjomataram I, et al. Global cancer statistics 2022: GLOBOCAN estimates of incidence and mortality worldwide for 36 cancers in 185 countries. CA Cancer J Clin. 2024;74:229–63. 38572751 10.3322/caac.21834

[2] Planchard D, Jänne PA, Cheng Y, Yang JC, Yanagitani N, Kim SW, et al. Osimertinib with or without chemotherapy in EGFR-mutated advanced NSCLC. N Engl J Med. 2023;389:1935–48. 37937763 10.1056/NEJMoa2306434

[3] Zhou C, Tang KJ, Cho BC, Liu B, Paz-Ares L, Cheng S, et al. Amivantamab plus chemotherapy in NSCLC with EGFR exon 20 insertions. N Engl J Med. 2023;389:2039–51. 37870976 10.1056/NEJMoa2306441

[4] de Langen AJ, Johnson ML, Mazieres J, Dingemans AC, Mountzios G, Pless M, et al. Sotorasib versus docetaxel for previously treated non-small-cell lung cancer with KRAS(G12C) mutation: a randomised, open-label, phase 3 trial. Lancet. 2023;401:733–46. 36764316 10.1016/S0140-6736(23)00221-0

[5] Reck M, Carbone DP, Garassino M, Barlesi F. Targeting KRAS in non-small-cell lung cancer: recent progress and new approaches. Ann Oncol. 2021;32:1101–10. 34089836 10.1016/j.annonc.2021.06.001

[6] Tan AC, Tan DSW. Targeted therapies for lung cancer patients with oncogenic driver molecular alterations. J Clin Oncol. 2022;40:611–25. 34985916 10.1200/JCO.21.01626

[7] Liu WJ, Du Y, Wen R, Yang M, Xu J. Drug resistance to targeted therapeutic strategies in non-small cell lung cancer. Pharmacol Ther. 2020;206:107438. 31715289 10.1016/j.pharmthera.2019.107438

[8] Cooper AJ, Sequist LV, Lin JJ. Third-generation EGFR and ALK inhibitors: mechanisms of resistance and management. Nat Rev Clin Oncol. 2022;19:499–514. 35534623 10.1038/s41571-022-00639-9 PMC9621058

[9] Fu K, Xie F, Wang F, Fu L. Therapeutic strategies for EGFR-mutated non-small cell lung cancer patients with osimertinib resistance. J Hematol Oncol. 2022;15:173. 36482474 10.1186/s13045-022-01391-4 PMC9733018

[10] Kastenhuber ER, Lowe SW. Putting p53 in context. Cell. 2017;170:1062–78. 28886379 10.1016/j.cell.2017.08.028 PMC5743327

[11] Hernández Borrero LJ, El-Deiry WS. Tumor suppressor p53: biology, signaling pathways, and therapeutic targeting. Biochim Biophys Acta Rev Cancer. 2021;1876:188556. 33932560 10.1016/j.bbcan.2021.188556 PMC8730328

[12] Liu Y, Su Z, Tavana O, Gu W. Understanding the complexity of p53 in a new era of tumor suppression. Cancer Cell. 2024;42:946–67. 38729160 10.1016/j.ccell.2024.04.009 PMC11190820

[13] Vaddavalli PL, Schumacher B. The p53 network: cellular and systemic DNA damage responses in cancer and aging. Trends Genet. 2022;38:598–612. 35346511 10.1016/j.tig.2022.02.010

[14] Hassin O, Oren M. Drugging p53 in cancer: one protein, many targets. Nat Rev Drug Discov. 2023;22:127-144. 10.1038/s41573-022-00571-8 PMC9549847 36216888

[15] Tuval A, Strandgren C, Heldin A, Palomar-Siles M, Wiman KG. Pharmacological reactivation of p53 in the era of precision anticancer medicine. Nat Rev Clin Oncol. 2024;21:106–20. 38102383 10.1038/s41571-023-00842-2

[16] Peuget S, Zhou X, Selivanova G. Translating p53-based therapies for cancer into the clinic. Nat Rev Cancer. 2024;24:192–215. 38287107 10.1038/s41568-023-00658-3

[17] Vidya E, Duchaine TF. Eukaryotic mRNA decapping activation. Front Genet. 2022;13:832547. 35401681 10.3389/fgene.2022.832547 PMC8984151

[18] Ramanathan A, Robb GB, Chan SH. mRNA capping: biological functions and applications. Nucleic Acids Res. 2016;44:7511–26. 27317694 10.1093/nar/gkw551 PMC5027499

[19] Franks TM, Lykke-Andersen J. The control of mRNA decapping and P-body formation. Mol Cell. 2008;32:605–15. 19061636 10.1016/j.molcel.2008.11.001 PMC2630519

[20] Liu Q, Yi D, Ding J, Mao Y, Wang S, Ma L, et al. MOV10 recruits DCP2 to decap human LINE-1 RNA by forming large cytoplasmic granules with phase separation properties. EMBO Rep. 2023;24:e56512. 37437058 10.15252/embr.202256512 PMC10481665

[21] Mei Y, Lv Q, Tan Z, Zhang Z, Ji Y, Chen S, et al. Decapping enzyme 2 is a novel immune-related biomarker that predicts poor prognosis in glioma. Biotechnol Genet Eng Rev. 2024;40:4262–4283. 10.1080/02648725.2023.2209409 37191010

[22] Wu H, Zhang J, Bai Y, Zhang S, Zhang Z, Tong W, et al. DCP1A is an unfavorable prognostic-related enhancer RNA in hepatocellular carcinoma. Aging. 2021;13:23020–35. 34609335 10.18632/aging.203593 PMC8544297

[23] Ruan T, Zhang Y, Liu W, Li Y, Wang D, Du Z, et al. Expression of DCP1a in gastric cancer and its biological function and mechanism in chemotherapy resistance in gastric cancer cells. Dig Liver Dis. 2020;52:1351–8. 32646734 10.1016/j.dld.2020.06.031

[24] Wu C, Zhu X, Tao K, Liu W, Ruan T, Wan W, et al. MALAT1 promotes the colorectal cancer malignancy by increasing DCP1A expression and miR203 downregulation. Mol Carcinog. 2018;57:1421–31. 29964337 10.1002/mc.22868

[25] Wu C, Liu W, Ruan T, Zhu X, Tao K, Zhang W. Overexpression of mRNA-decapping enzyme 1a affects survival rate in colorectal carcinoma. Oncol Lett. 2018;16:1095–100. 29963186 10.3892/ol.2018.8730 PMC6019971

[26] Tang Y, Xie C, Zhang Y, Qin Y, Zhang W. Overexpression of mRNA-decapping enzyme 1a predicts disease-specific survival in malignant melanoma. Melanoma Res. 2018;28:30–6. 29076924 10.1097/CMR.0000000000000406

[27] Chen H, Carrot-Zhang J, Zhao Y, Hu H, Freeman SS, Yu S, et al. Genomic and immune profiling of pre-invasive lung adenocarcinoma. Nat Commun. 2019;10:5472. 31784532 10.1038/s41467-019-13460-3 PMC6884501

[28] Smeenk L, van Heeringen SJ, Koeppel M, van Driel MA, Bartels SJ, Akkers RC, et al. Characterization of genome-wide p53-binding sites upon stress response. Nucleic Acids Res. 2008;36:3639–54. 18474530 10.1093/nar/gkn232 PMC2441782

[29] Wang W, Han D, Cai Q, Shen T, Dong B, Lewis MT, et al. MAPK4 promotes triple negative breast cancer growth and reduces tumor sensitivity to PI3K blockade. Nat Commun. 2022;13:245. 35017531 10.1038/s41467-021-27921-1 PMC8752662

[30] Wang W, Shen T, Dong B, Creighton CJ, Meng Y, Zhou W, et al. MAPK4 overexpression promotes tumor progression via noncanonical activation of AKT/mTOR signaling. J Clin Invest. 2019;129:1015–29. 30688659 10.1172/JCI97712 PMC6391107

[31] Menendez D, Nguyen TA, Freudenberg JM, Mathew VJ, Anderson CW, Jothi R, et al. Diverse stresses dramatically alter genome-wide p53 binding and transactivation landscape in human cancer cells. Nucleic Acids Res. 2013;41:7286–301. 23775793 10.1093/nar/gkt504 PMC3753631

[32] Chen TW, Liao HW, Noble M, Siao JY, Cheng YH, Chiang WC, et al. Human DCP1 is crucial for mRNA decapping and possesses paralog-specific gene regulating functions. eLife. 2024;13:RP94811. 10.7554/eLife.94811 PMC11530239 39485278

[33] Zhao Y, Pan Y, Cheng C, Zheng D, Zhang Y, Gao Z, et al. EGFR-mutant lung adenocarcinoma harboring co-mutational tumor suppressor genes predicts poor prognosis. J Cancer Res Clin Oncol. 2020;146:1781–9. 32361787 10.1007/s00432-020-03237-3 PMC11804650

[34] Zhang Y, Ma Y, Li Y, Shen X, Yu Y, Pan Y, et al. Comparative analysis of co-occurring mutations of specific tumor suppressor genes in lung adenocarcinoma between Asian and Caucasian populations. J Cancer Res Clin Oncol. 2019;145:747–57. 30673871 10.1007/s00432-018-02828-5 PMC11810360

[35] Shen T, Wang W, Zhou W, Coleman I, Cai Q, Dong B, et al. MAPK4 promotes prostate cancer by concerted activation of androgen receptor and AKT. J Clin Invest. 2021;131:e135465. 10.1172/JCI135465 PMC7880415 33586682

[36] Tian S, Lou L, Tian M, Lu G, Tian J, Chen X. MAPK4 deletion enhances radiation effects and triggers synergistic lethality with simultaneous PARP1 inhibition in cervical cancer. J Exp Clin Cancer Res. 2020;39:143. 32711558 10.1186/s13046-020-01644-5 PMC7382858

