# Supplementary material for: p53 transcriptionally activates DCP1B to suppress tumor progression and enhance tumor sensitivity to PI3K blockade in non-small cell lung cancer
Source: Cell Death Differ. 2025 Apr 9;32(9):1722–33. doi: 10.1038/s41418-025-01501-y (PMC12432164; doi:10.1038/s41418-025-01501-y)
Supplement: Supplementary file 1 — Supplementary Figures [file 41418_2025_1501_MOESM1_ESM.docx]

**Figure S1.**


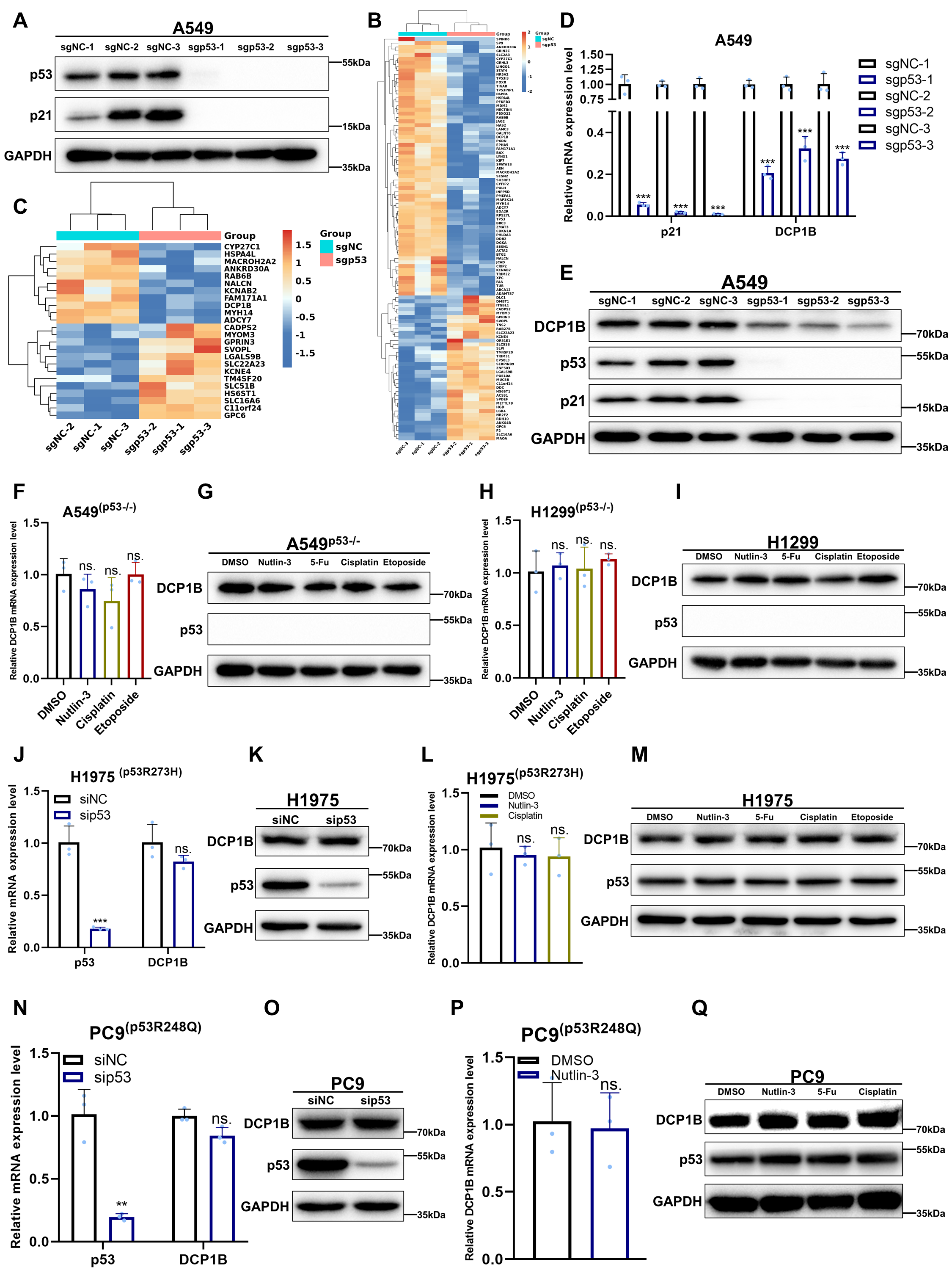


**The level of DCP1B is regulated by wild-type p53 but not mutant p53, Related to Figure 1. A-C**, Heatmaps of the RNA-seq results showing the upregulated and downregulated genes in A549 cells transfected with sgNC and sgp53 lentiviral. **D** and **E**, RT-Qpcr and WB assays validating the expression levels of DCP1B in the corresponding duplicate samples of the RNA-seq. **F-I**, RT-qPCR and WB assays validating the expression levels of DCP1B in A549 (p53^-/-^) and H1299 cells treated with p53-inducing agents including Nutlin-3, Cisplatin, 5-Fluorouracil (5-FU), and Etoposide for 24 hours. **J-Q**, RT-qPCR and WB assays validating the expression levels of DCP1B in H1975 (p53^R172H^) and PC9 (p53^R248Q^) cells treated with p53-inducing agents including Nutlin-3, Cisplatin, 5-Fluorouracil (5-FU), and Etoposide for 24 hours or transfected with siNC and sip53. Data in (D), (F), (H), (J), (L), (N) and (P)are represented as mean ± SD, *n* = 3.

**Figure S2.**


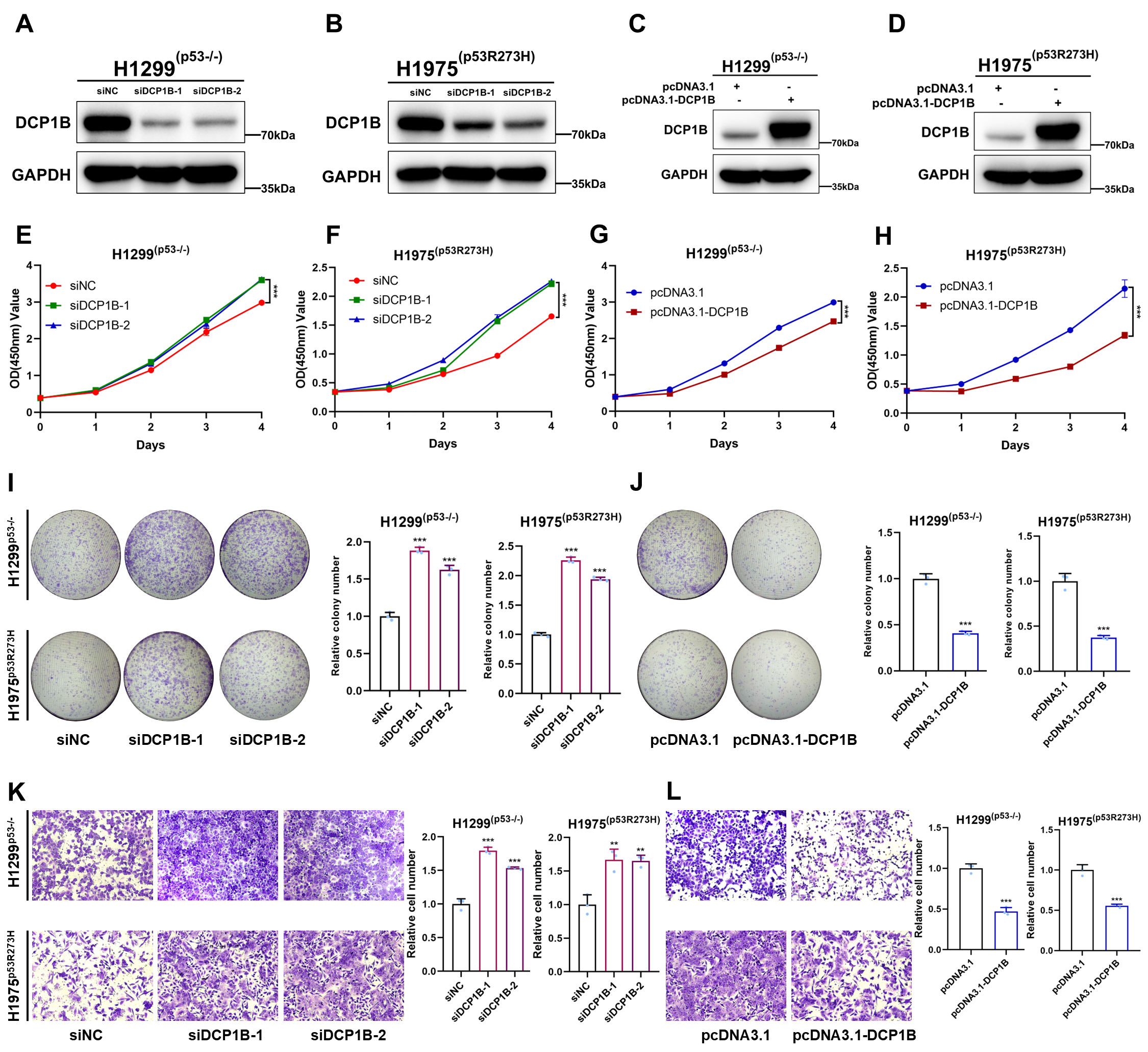
**DCP1B is a p53-independent tumor suppressor in NSCLC, Related to Figure 2.** **A-D**, Expression validation of DCP1B in H1299 and H1975 cells transfected with siNC and siDCP1B (#1, #2) or Flag-NC and Flag-DCP1B plasmids by WB. **E-L**, H1299 and H1975 cells transfected with siNC and siDCP1B (#1, #2) or Flag-NC and Flag-DCP1B plasmids were then subjected to cell proliferation, colony formation, and cell migration assays, representative images and corresponding quantitative results of colony formation and cell migration assays are shown. Data are represented as mean ± SD, n = 3.


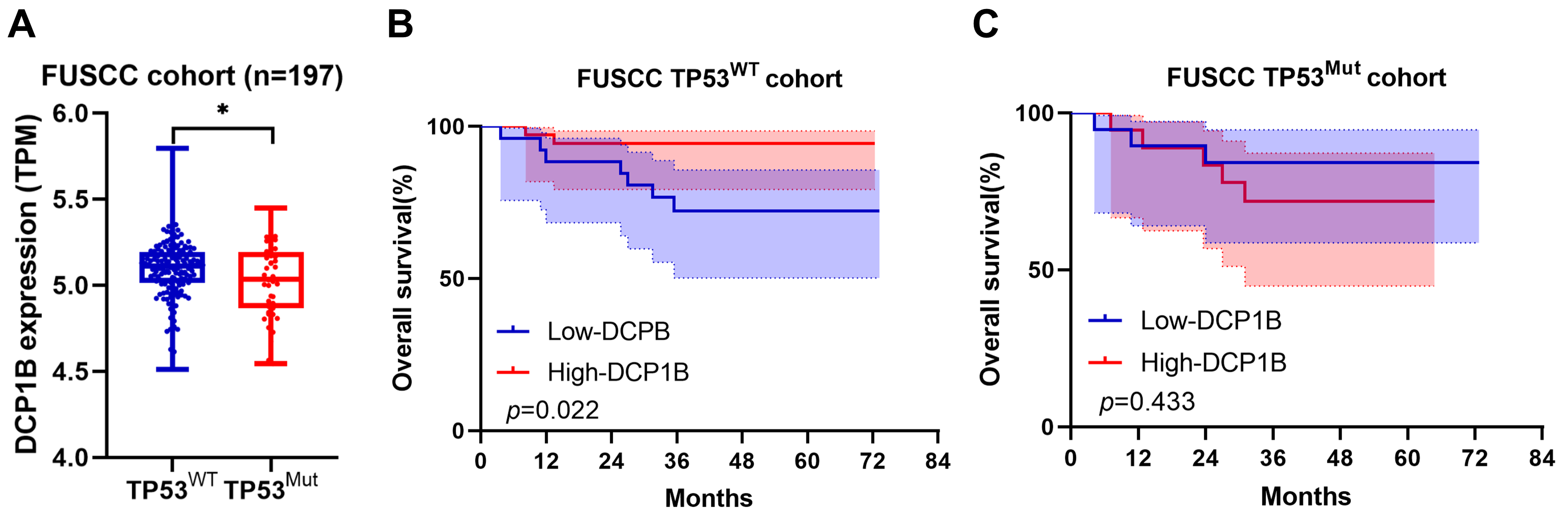
**Figure S3.**

**The expression level and prognostic significance of DCP1B exhibit distinct patterns in patients with wild-type *TP53* compared to those with mutant *TP53*, Related to Figure 3. A**, The expression level of DCP1B in FUSCC *TP53*^WT^ and *TP53*^Mut^ cohort. **B**, Kaplan–Meier analysis of OS in LUAD patients with high and low DCP1B expression in FUSCC *TP53*^WT^ cohort. **C**, Kaplan–Meier analysis of OS in LUAD patients with high and low DCP1B expression in FUSCC *TP53*^Mut^ cohort.

**Figure S4.**


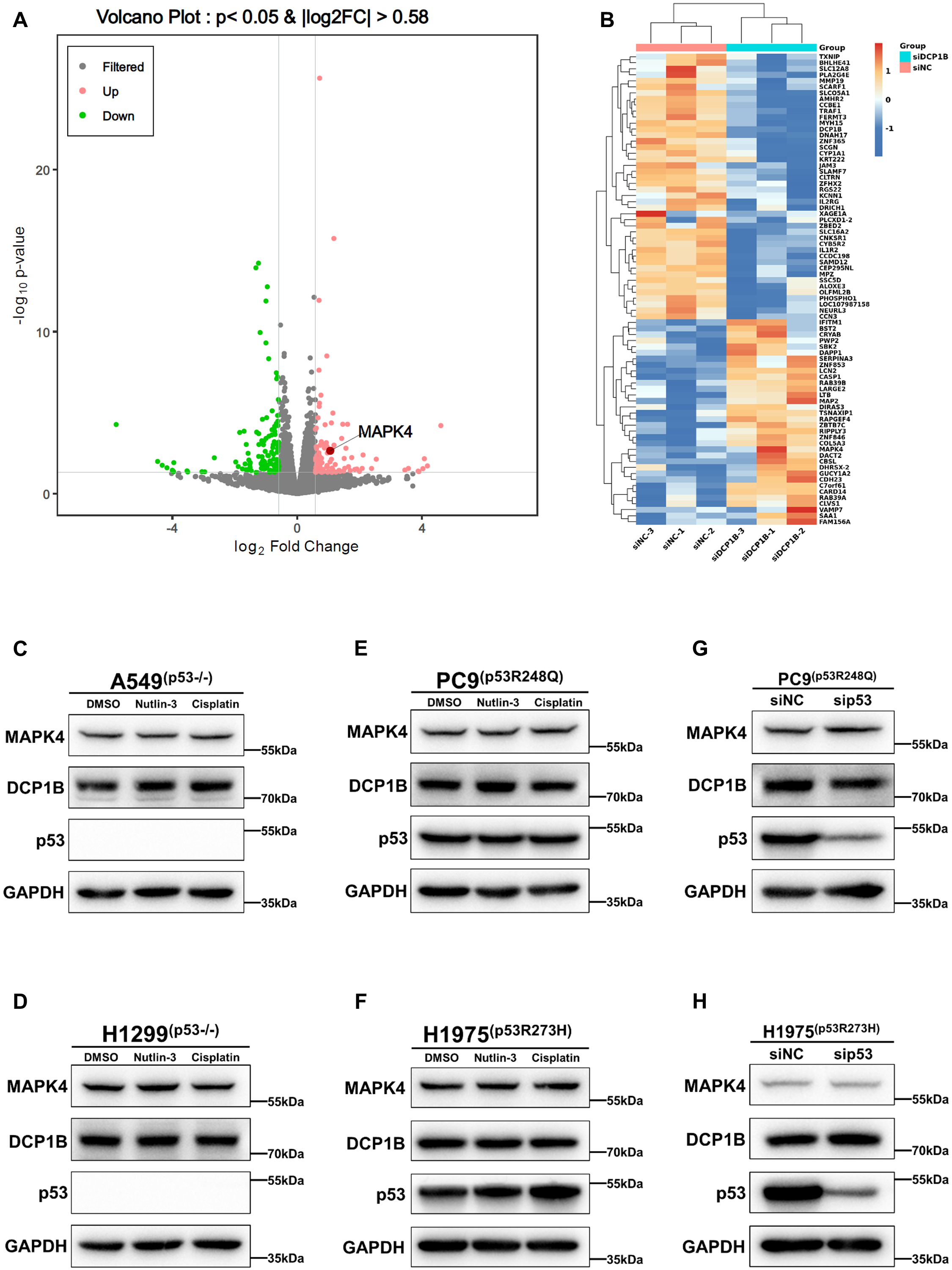


**MAPK4 is the downstream of the p53-DCP1B axis, Related to Figures 4 and 5. A** and **B**, Volcano plot and heatmaps of the RNA-seq results showing the upregulated and downregulated genes in A549 cells transfected with siNC and siDCP1B. **C-H**, WB assays validating the expression levels of MAPK4 in A549 (p53^-/-^), H1299, H1975 (p53^R172H^) and PC9 (p53^R248Q^) cells treated with p53-inducing agents including Nutlin-3, Cisplatin, 5-Fluorouracil (5-FU), and Etoposide for 24 hours or transfected with siNC and sip53.

**Figure S5.**


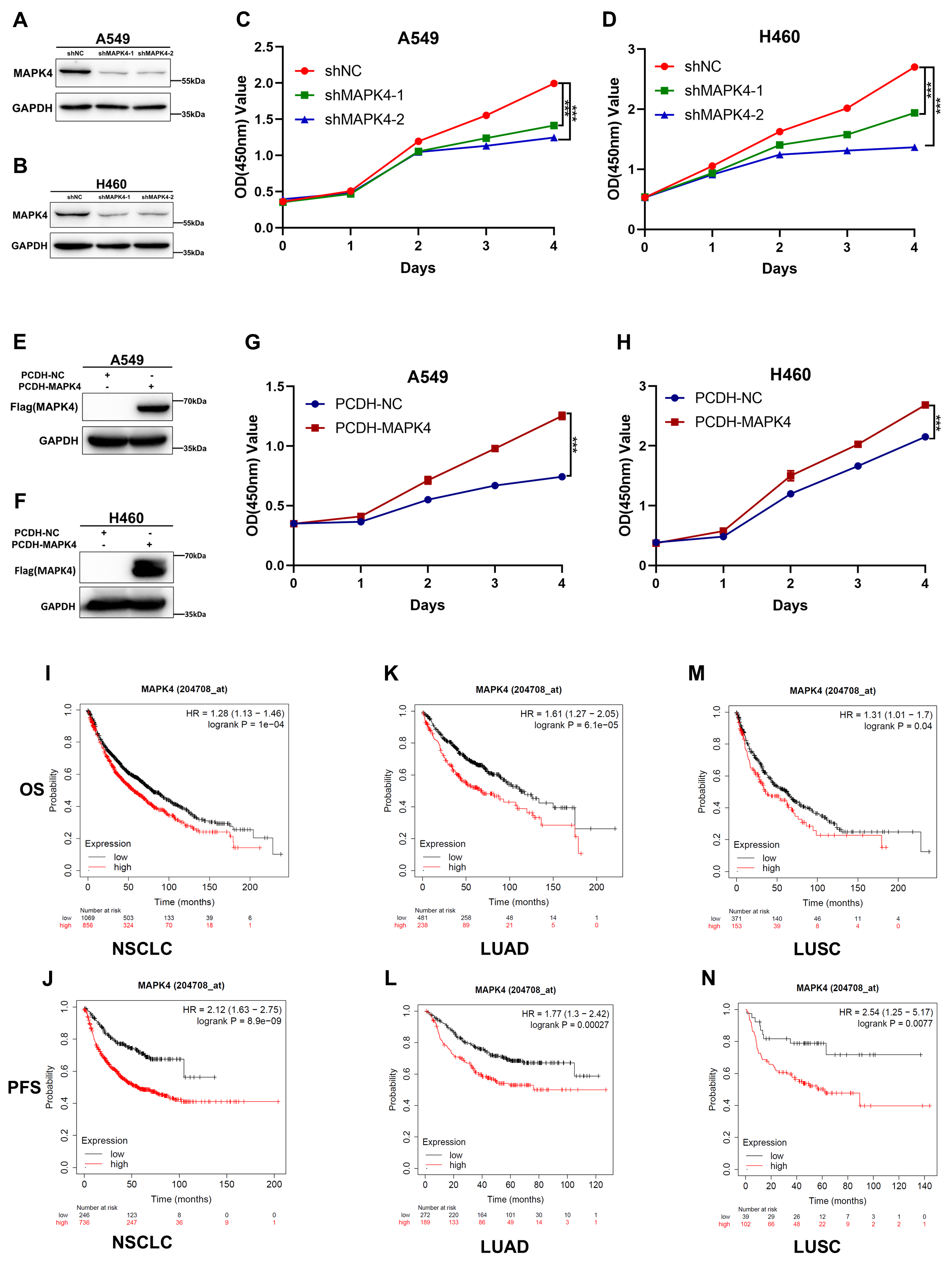


**MAPK4 is a tumor oncogenic factor in NSCLC, Related to Figures 5 and 6. A-D**, CCK-8 assays demonstrating the function of cell proliferation in A549 and H460 cells stably expressing shNC and shMAPK4(#1, #2). **E-H**, CCK-8 assays demonstrating the function of cell proliferation in A549 and H460 cells stably expressing PCDH and PCDH-MAPK4(#1, #2). Data are represented as mean ± SD, *n*=3. **I-N**, Kaplan–Meier analysis of the OS and PFS of NSCLC patients with high and low MAPK4 expression.
