# Supplementary material for: p53 transcriptionally activates DCP1B to suppress tumor progression and enhance tumor sensitivity to PI3K blockade in non-small cell lung cancer
Source: Cell Death Differ. 2025 Apr 9;32(9):1722–33. doi: 10.1038/s41418-025-01501-y (PMC12432164; doi:10.1038/s41418-025-01501-y)
Supplement: Supplementary file 2 — Supplementary Tables [file 41418_2025_1501_MOESM2_ESM.docx]

**Table S1. Reagents**

| **Reagents** | **Source** |
| --- | --- |
| Dimethyl sulfoxide, DMSO | MedChemExpress |
| Nutlin-3 | MedChemExpress |
| 5-Fluorouracil | MedChemExpress |
| Cisplatin | MedChemExpress |
| Etoposide | MedChemExpress |
| APG-115 | MedChemExpress |
| Alpelisib | MedChemExpress |
| Actinomycin D (Act.D) | MedChemExpress |
| RNAiso Plus（Trizol） | TaKaRa |
| ChamQ Universal SYBR qPCR Master Mix | Vazyme |
| Hieff trans® Liposomal Transfection Reagent | Yeasen |
| KOD-Plus-Mutagenesis Kit | TOYOBO |
| Cell Counting Kit-8 | Dojindo |
| Dual-luciferase Reporter Assay System | Promega |
| Magna ChIPTM A/G Chromatin  Immunoprecipitation Reagent | Merck |
| Hiscript III qRT SuperMix | Vazyme |

**Table S2. Antibodies**

| **Antibodies** | **Vendors** | **Catalog number** |
| --- | --- | --- |
| p53 (DO-1) | Santa Cruz | sc-126 |
| p21 | Cell Signaling Technology | 2947 |
| GAPDH | Proteintech | 60004-1-Ig |
| DCP1B | Cell Signaling Technology | 13233 |
| Flag tag | Sigma | F1804 |
| p-AKT | Cell Signaling Technology | 13038 |
| AKT | Cell Signaling Technology | 9272 |
| p-GSK3β | Cell Signaling Technology | 9336 |
| GSK3β | Cell Signaling Technology | 9315 |
| MAPK4 | Abcepta | AP7298b |
| Goat anti-Rabbit IgG antibody (HRP) | Proteintech | No.SA00001-2 |
| Goat anti-Mouse IgG antibody (HRP) | Proteintech | No. SA00001-1 |

**Table S3. Primers for plasmids construction.**

| **Primers** | **Sequences (5’-3’)** |
| --- | --- |
| p53-gRNA-F1 | CACCGGCATGGGCGGCATGAACCGG |
| p53-gRNA-R1 | AAACCCGGTTCATGCCGCCCATGCC |
| p53-gRNA-F2 | CACCGGGAGTCTTCCAGTGTGATGA |
| p53-gRNA-R2 | AAACTCATCACACTGGAAGACTCCC |
| p53-gRNA-F3 | CACCGGATCAAGATCATTCGGCGCC |
| p53-gRNA-R3 | AAACGGCGCCGAATGATCTTGATCC |
| pcDNA3.1-DCP1B-F | ATACGCGGATCCATGGCAGCCGTGGCGGCA |
| pcDNA3.1-DCP1B-R | GCGCCGCTCGAGTCACATAGTCTTTTTCATGGCTGC |
| pCDH-DCP1B-F | ATACGCTCTAGAATGGCAGCCGTGGCG |
| pCDH-DCP1B-R | CCGTTCGAATTAGTGGTGGTGGTGGTGGTGCT |
| PCDH-MAPK4-F | CGCTCTAGAGAGGATCCGGTACCGAGGAG |
| PCDH-MAPK4-R | CCG TTCGAATCGACGAATTTATCGTCGTCAT |
| sh-DCP1B-F1 | CCGGCCTAACTCAGTATGAACAGTTCTCGAGAACTGTTCATACTGAGTTAGGTTTTTG |
| sh-DCP1B-R1 | AATTCAAAAACCTAACTCAGTATGAACAGTTCTCGAGAACTGTTCATACTGAGTTAGG |
| sh-DCP1B-F2 | CCGGCCTCATTCAGAATGATGACAACTCGAGTTGTCATCATTCTGAATGAGGTTTTTG |
| sh-DCP1B-R2 | AATTCAAAAACCTCATTCAGAATGATGACAACTCGAGTTGTCATCATTCTGAATGAGG |
| sh-MAPK4-F1 | CCGGACTACACCAAAGCCATCGACACTCGAGTGTCGATGGCTTTGGTGTAGTTTTTTG |
| sh-MAPK4-R1 | AATTCAAAAAACTACACCAAAGCCATCGACACTCGAGTGTCGATGGCTTTGGTGTAGT |
| sh-MAPK4-F2 | CCGGAGTGAACAGTGAAGCCATCGACTCGAGTCGATGGCTTCACTGTTCACTTTTTTG |
| sh-MAPK4-R2 | AATTCAAAAAAGTGAACAGTGAAGCCATCGACTCGAGTCGATGGCTTCACTGTTCACT |

**Table S4. Primers for siRNAs**

| **siRNA** | **Sequences (5’-3’)** |
| --- | --- |
| siNC | UUCUCCGAACGUGUCACGUTT |
| sip53 | GUAAUCUACUGGGACGGAATT |
| siDCP1B-1 | GAAGUAGACAUUUUACGAATT |
| siDCP1B-2 | GUGUGACCCUAGUACACCATT |
| siDCP1B-3 | GGAUCAACAAGACACCCAATT |

**Table S5. Primers for RT-qPCR.**

| Primers | **Sequences (5’-3’)** |
| --- | --- |
| GAPDH-F | GGAGCGAGATCCCTCCAAAAT |
| GAPDH-R | GGCTGTTGTCATACTTCTCATGG |
| p53-F | CCCAAGCAATGGATGATTTGA |
| p53-R | GGCATTCTGGGAGCTTCATCT |
| p21-F | CTGGACTGTTTTCTCTCGGCTC |
| p21-R | TGTATATTCAGCATTGTGGGAGGA |
| DCP1B-F | CTCTGTACACCTTCGGCCAT |
| DCP1B-R | TCCATAGATGGACAATCTGGCA |
| CHIP-DCP1B-F | GCCCCTGCCTTGATTGGGTT |
| CHIP-DCP1B-R | ACCCTTTCAGGGGATCTGCA |
| CHIP-p21-F | GCTCCCTCATGGGCAAACTCACT |
| CHIP-p21-R | TGGCTGGTCTACCTGGCTCCTCT |
| CHIP-GAPDH-F | TACTAGCGGTTTTACGGGCG |
| CHIP-GAPDH-R | TCGAACAGGAGGAGCAGAGAGCGA |
| MAPK4-F | GCTGAGAAGGGTGACTGCAT |
| MAPK4-R | CCGAGCACCTCGTACACTTT |
| RIP-MAPK4-F | TGAGAAGGGTGACTGCATCG |
| RIP-MAPK4-R | ACCAAACCATTGACACCGAAG |

**Table S6. Relationship between DCP1B expression and clinicopathologic characteristics in NSCLC patients, Related to Figure 3.**

| **Characteristic** | **NSCLC Patients, N (%)** | | ***P* Value** |
| --- | --- | --- | --- |
|  | **Low-DCP1B**  **N=202(49.5%)** | **High-DCP1B**  **N=206(50.5%)** |  |
| **Age (years)** | | | 0.075 |
| ≤60 | 93(45.1%) | 113(54.9%) |  |
| >60 | 109(54.0%) | 93(46.0%) |  |
| **Gender** | | | **0.016** |
| Male | 125(54.8%) | 103(45.2%) |  |
| Female | 77(42.8%) | 103(57.2%) |  |
| **Smoking history** | | | **0.002** |
| Ever | 101(58.4%) | 72(41.6%) |  |
| Never | 101(43.0%) | 134(57.0%) |  |
| **Tumor size** | | | **0.034** |
| ≤3cm | 121(45.7%) | 144(54.3%) |  |
| >3cm | 81(56.6%) | 62(43.4%) |  |
| **N stage** | | | 0.163 |
| Ever | 126(47.0%) | 142(53.0%) |  |
| Never | 76(54.3%) | 64(45.7%) |  |
| **p-TNM stage** | | | 0.061 |
| Ⅰ | 104(45.4%) | 125(54.6%) |  |
| Ⅱ/Ⅲ | 98(54.7%) | 81(45.3%) |  |
| **Histological type** | | | **<0.001** |
| LUAD | 156(50.5%) | 153(49.5%) |  |
| AIS/MIA/LPA | 10(30.3%) | 23(69.7%) |  |
| APA/PPA/IMA | 114(48.1%) | 123(51.9%) |  |
| MPA/SPA | 32(82.1%) | 7(17.9%) |  |
| LUSC | 46(46.5%) | 53(53.5%) |  |
| **VPI** | | | 0.100 |
| Absent | 134(46.9%) | 152(53.1%) |  |
| Present | 68(55.7%) | 54(44.3%) |  |
| **LVI** | | | 0.749 |
| Absent | 171(49.9%) | 172(50.1%) |  |
| Present | 31(47.7%) | 34(52.3%) |  |
