# Supplementary material for: p53 transcriptionally activates DCP1B to suppress tumor progression and enhance tumor sensitivity to PI3K blockade in non-small cell lung cancer
Source: Cell Death Differ. 2025 Apr 9;32(9):1722–33. doi: 10.1038/s41418-025-01501-y (PMC12432164; doi:10.1038/s41418-025-01501-y)
Supplement: Supplementary file 3 — Uncropped WB [file 41418_2025_1501_MOESM3_ESM.pptx]

## Slide 1
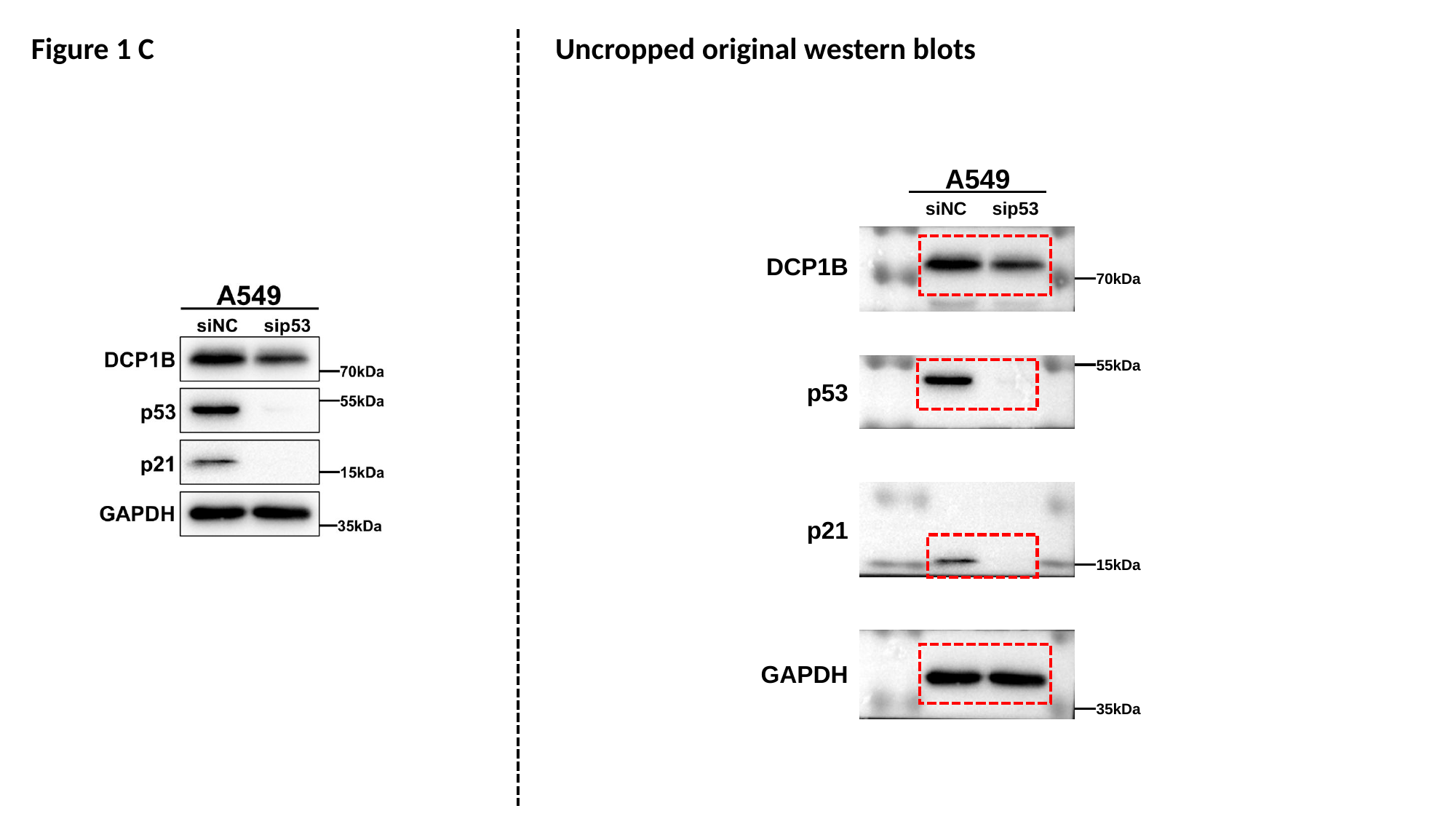

Figure 1 C
Uncropped original western blots
A549
 siNC sip53
DCP1B
70kDa
55kDa
p53
p21
15kDa
GAPDH
35kDa

## Slide 2
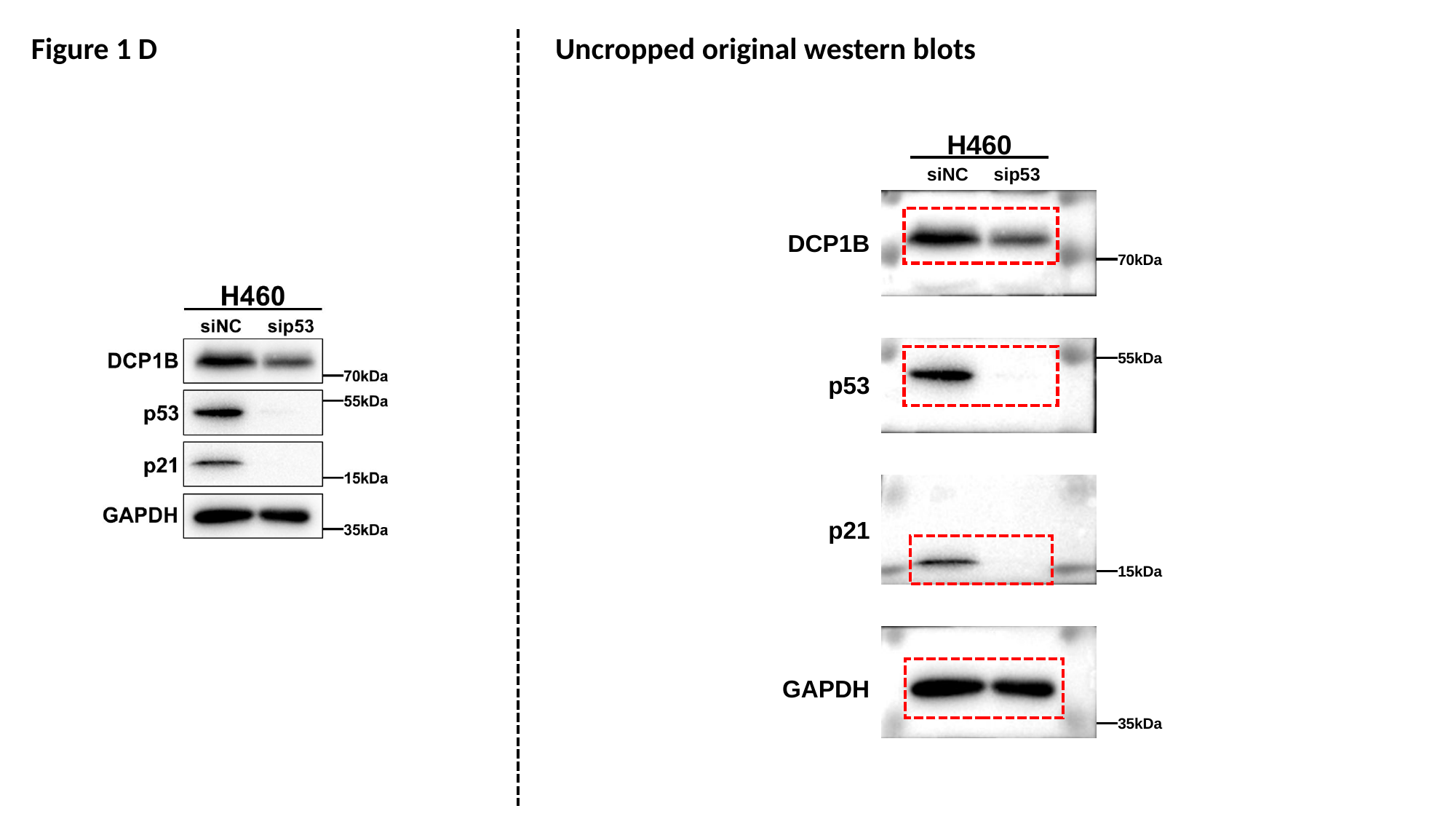

Figure 1 D
Uncropped original western blots
H460
 siNC sip53
DCP1B
70kDa
55kDa
p53
p21
15kDa
GAPDH
35kDa

## Slide 3
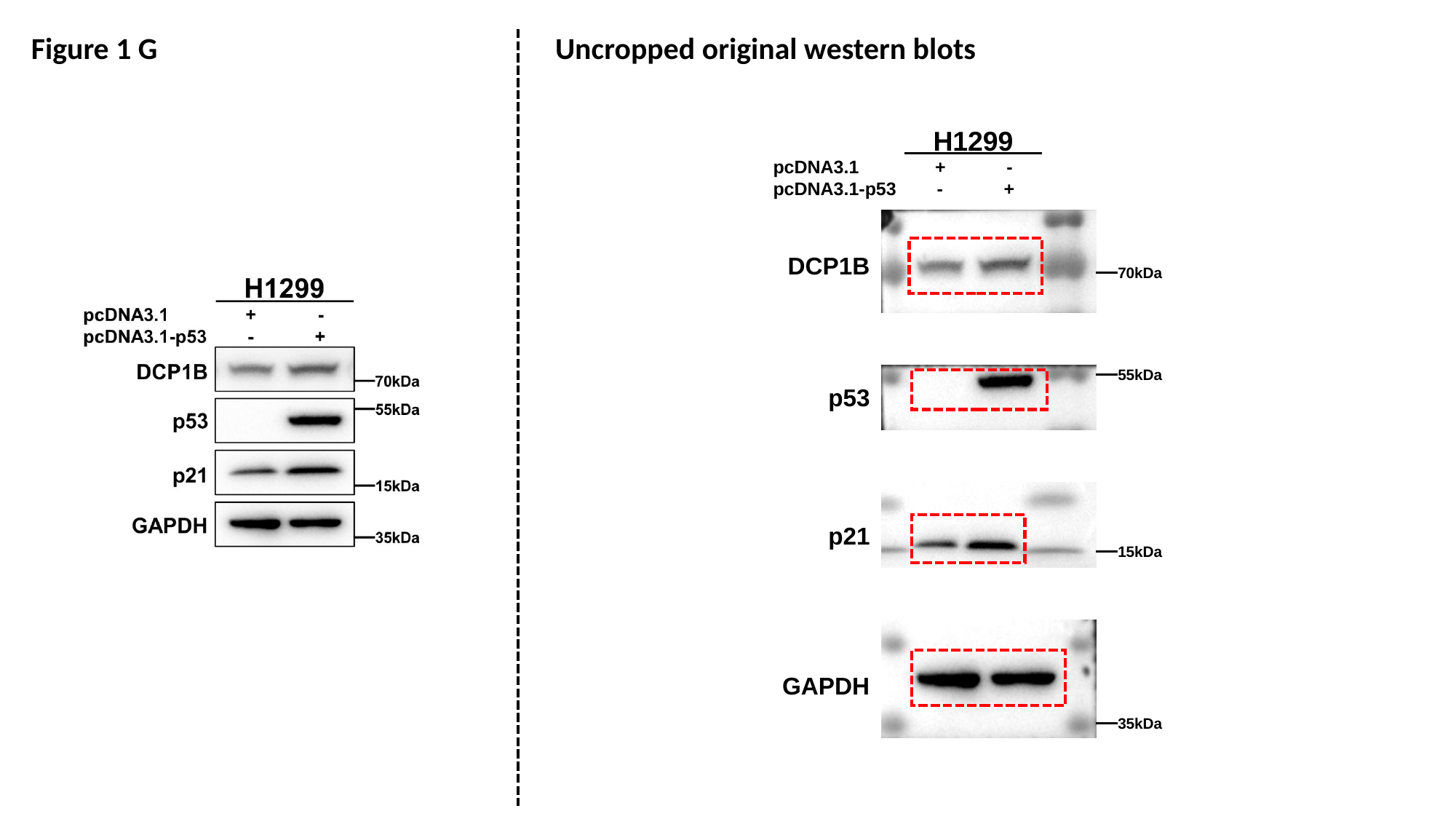

Figure 1 G
Uncropped original western blots
H1299
pcDNA3.1 + -
pcDNA3.1-p53 - +
DCP1B
70kDa
55kDa
p53
p21
15kDa
GAPDH
35kDa

## Slide 4
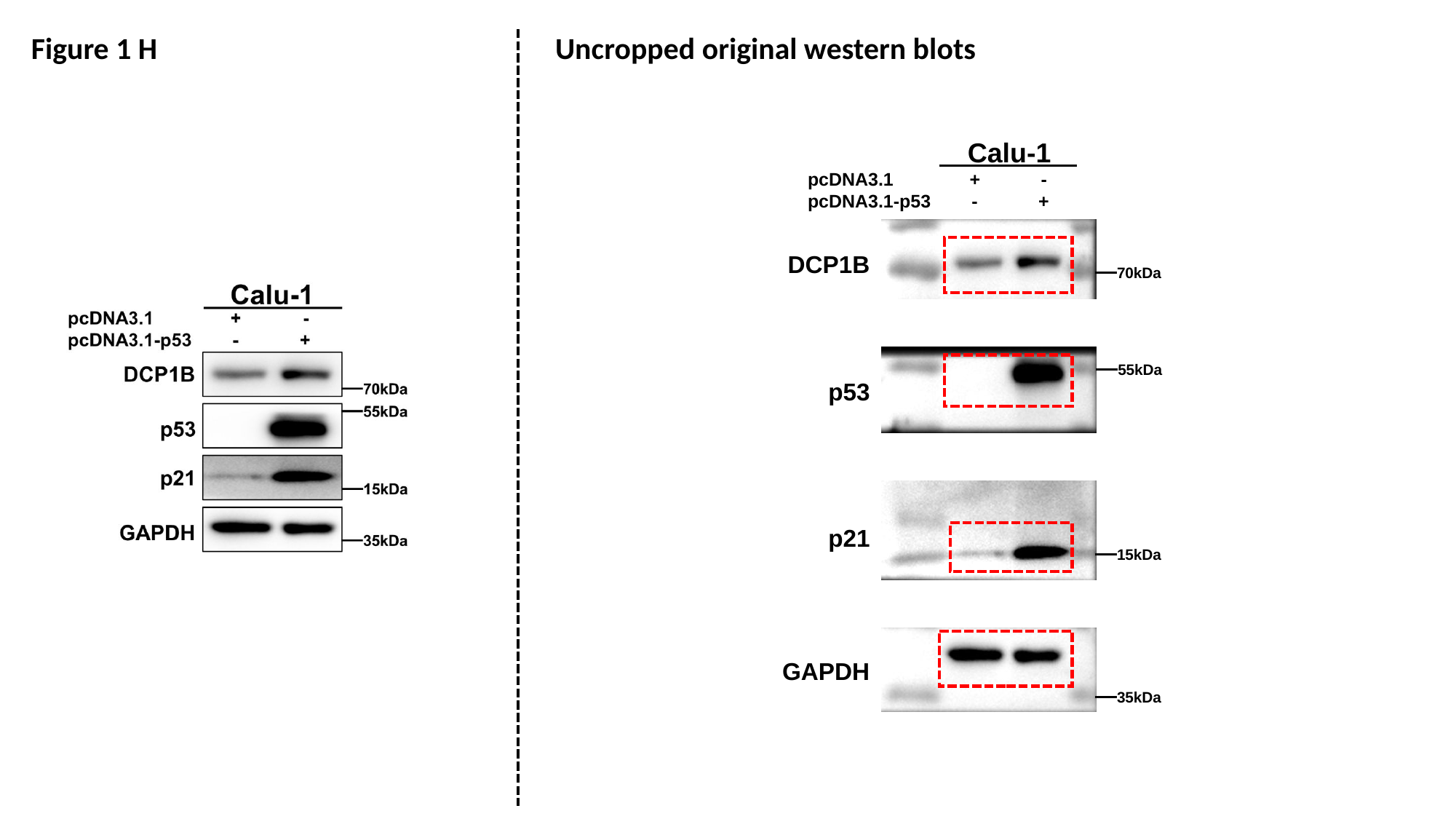

Figure 1 H
Uncropped original western blots
Calu-1
pcDNA3.1 + -
pcDNA3.1-p53 - +
DCP1B
70kDa
55kDa
p53
p21
15kDa
GAPDH
35kDa

## Slide 5
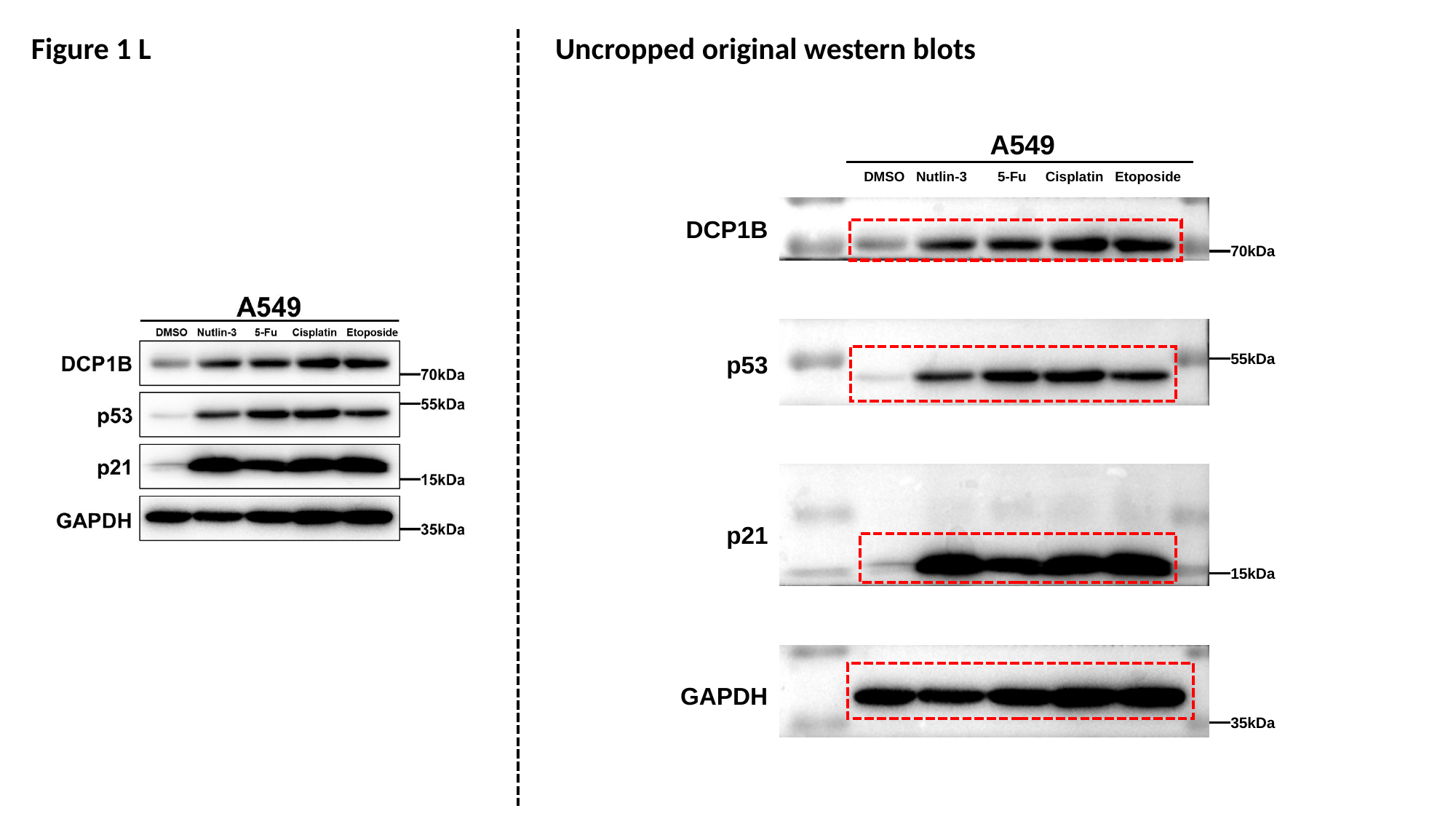

Figure 1 L
Uncropped original western blots
A549
DMSO Nutlin-3 5-Fu Cisplatin Etoposide
DCP1B
70kDa
55kDa
p53
p21
15kDa
GAPDH
35kDa

## Slide 6
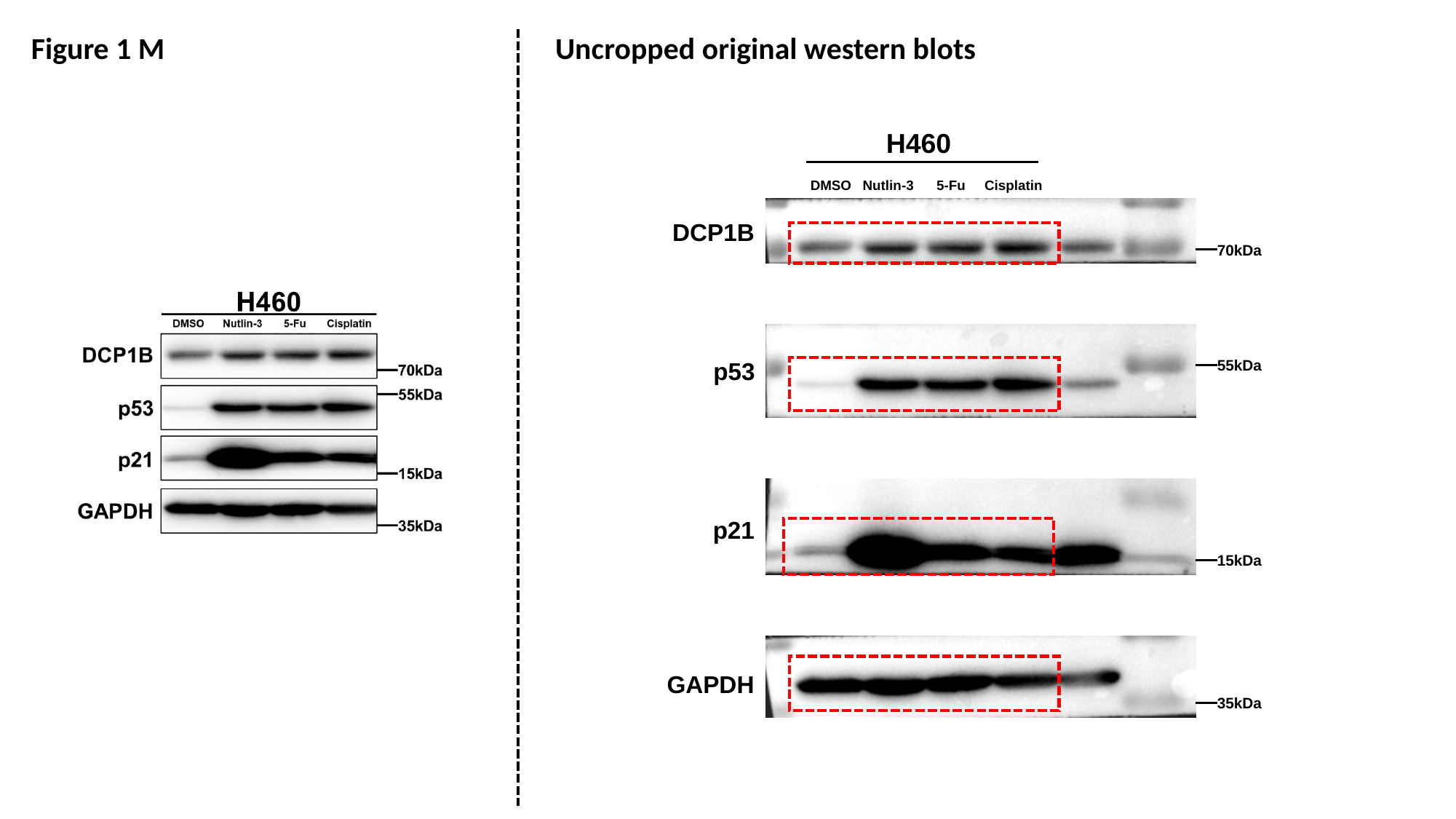

Figure 1 M
Uncropped original western blots
H460
DMSO Nutlin-3 5-Fu Cisplatin
DCP1B
70kDa
55kDa
p53
p21
15kDa
GAPDH
35kDa

## Slide 7
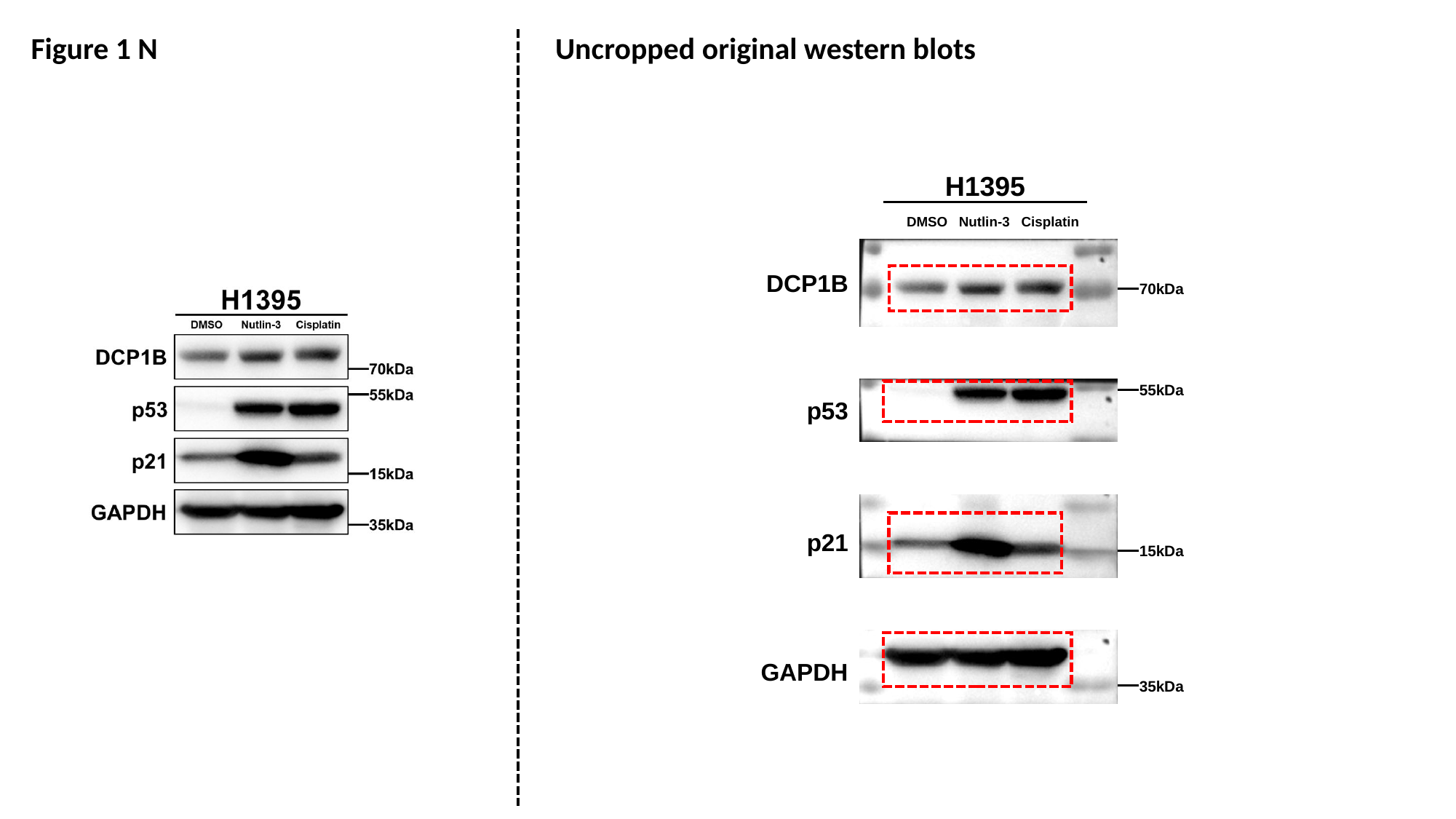

Figure 1 N
Uncropped original western blots
H1395
DMSO Nutlin-3 Cisplatin
DCP1B
70kDa
55kDa
p53
p21
15kDa
GAPDH
35kDa

## Slide 8
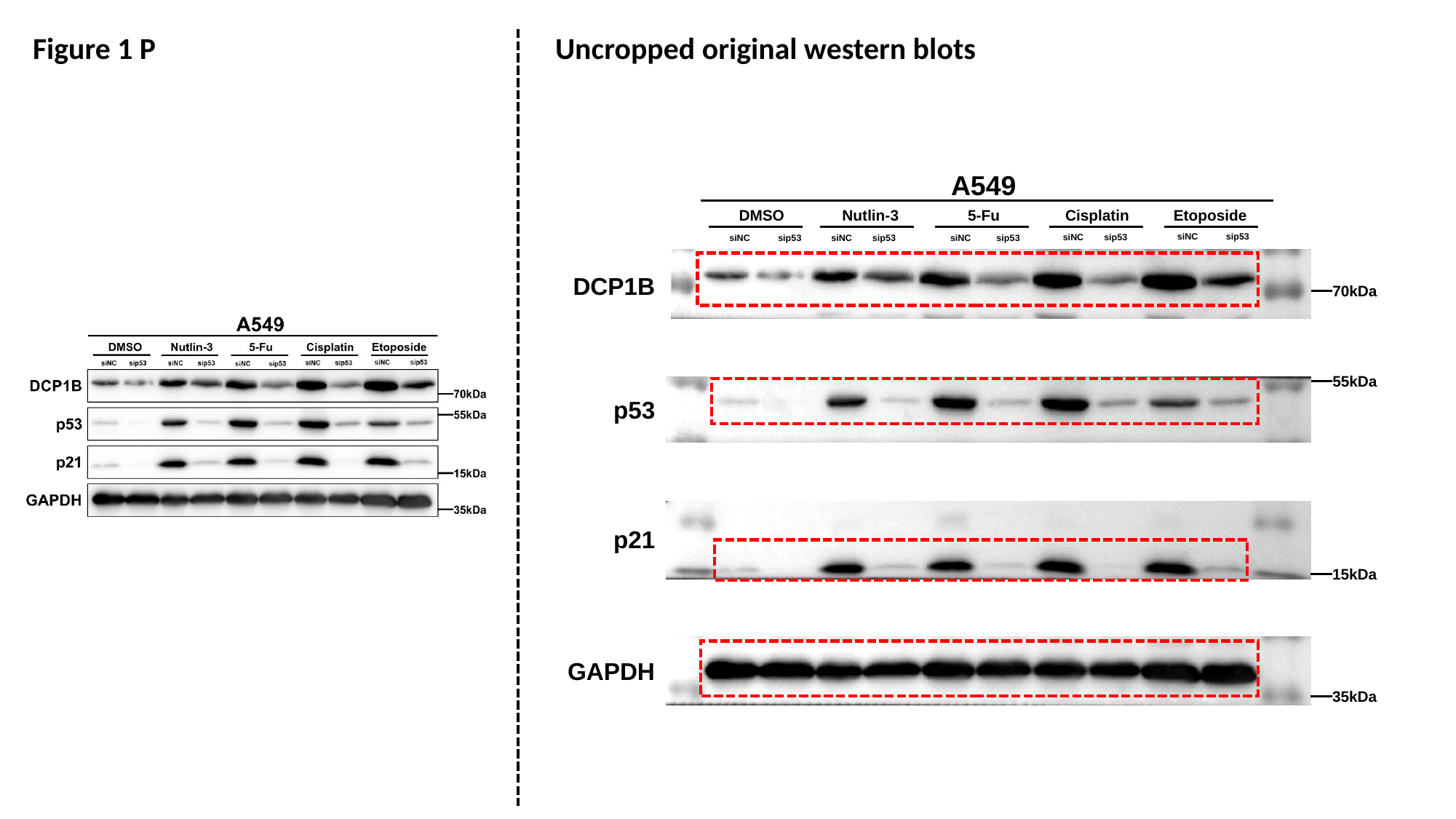

Figure 1 P
Uncropped original western blots
A549
Etoposide
DMSO
Nutlin-3
5-Fu
Cisplatin
 siNC sip53
 siNC sip53
 siNC sip53
 siNC sip53
 siNC sip53
DCP1B
70kDa
55kDa
p53
p21
15kDa
GAPDH
35kDa

## Slide 9
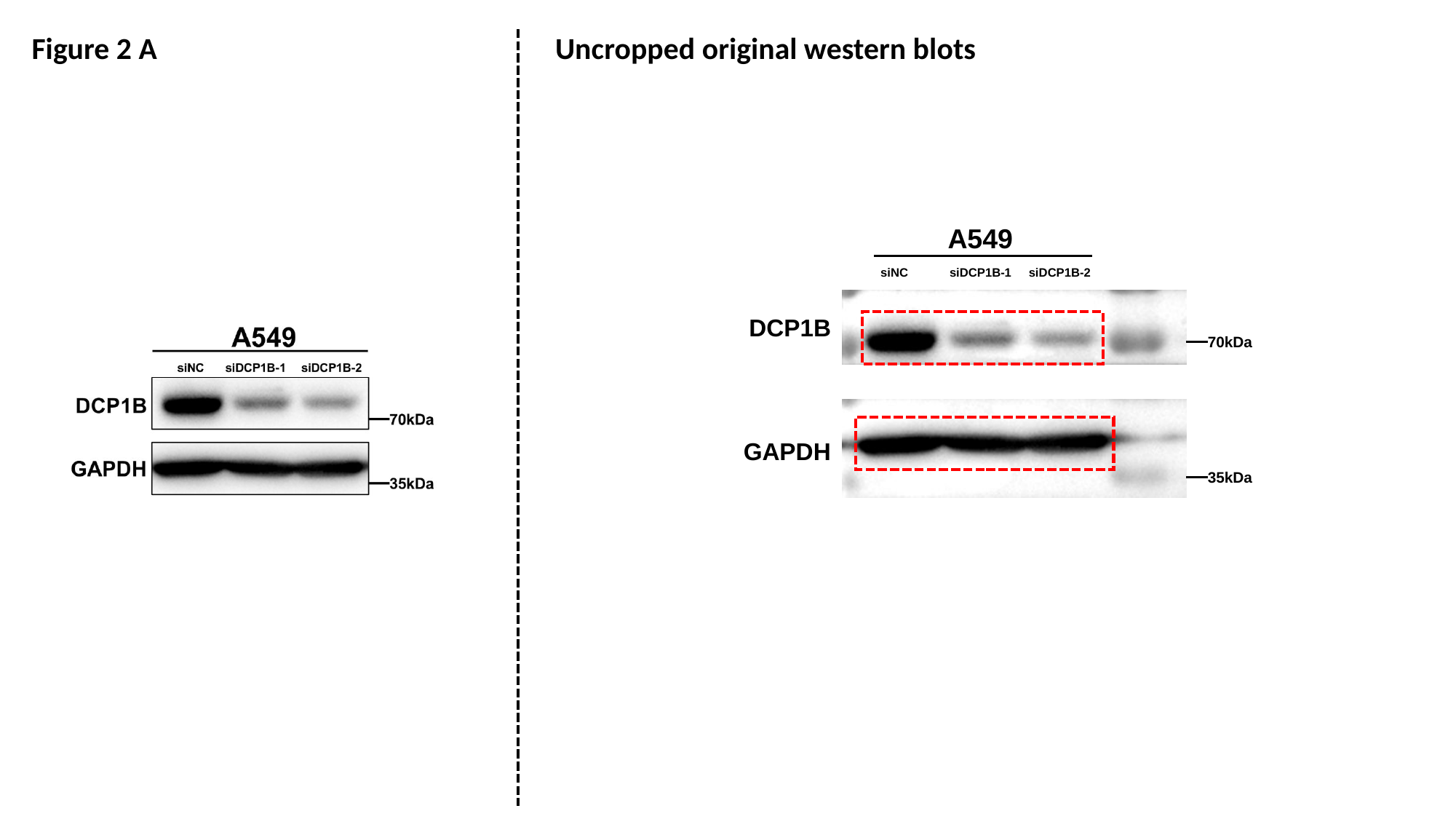

Figure 2 A
Uncropped original western blots
A549
 siNC siDCP1B-1 siDCP1B-2
DCP1B
70kDa
GAPDH
35kDa

## Slide 10
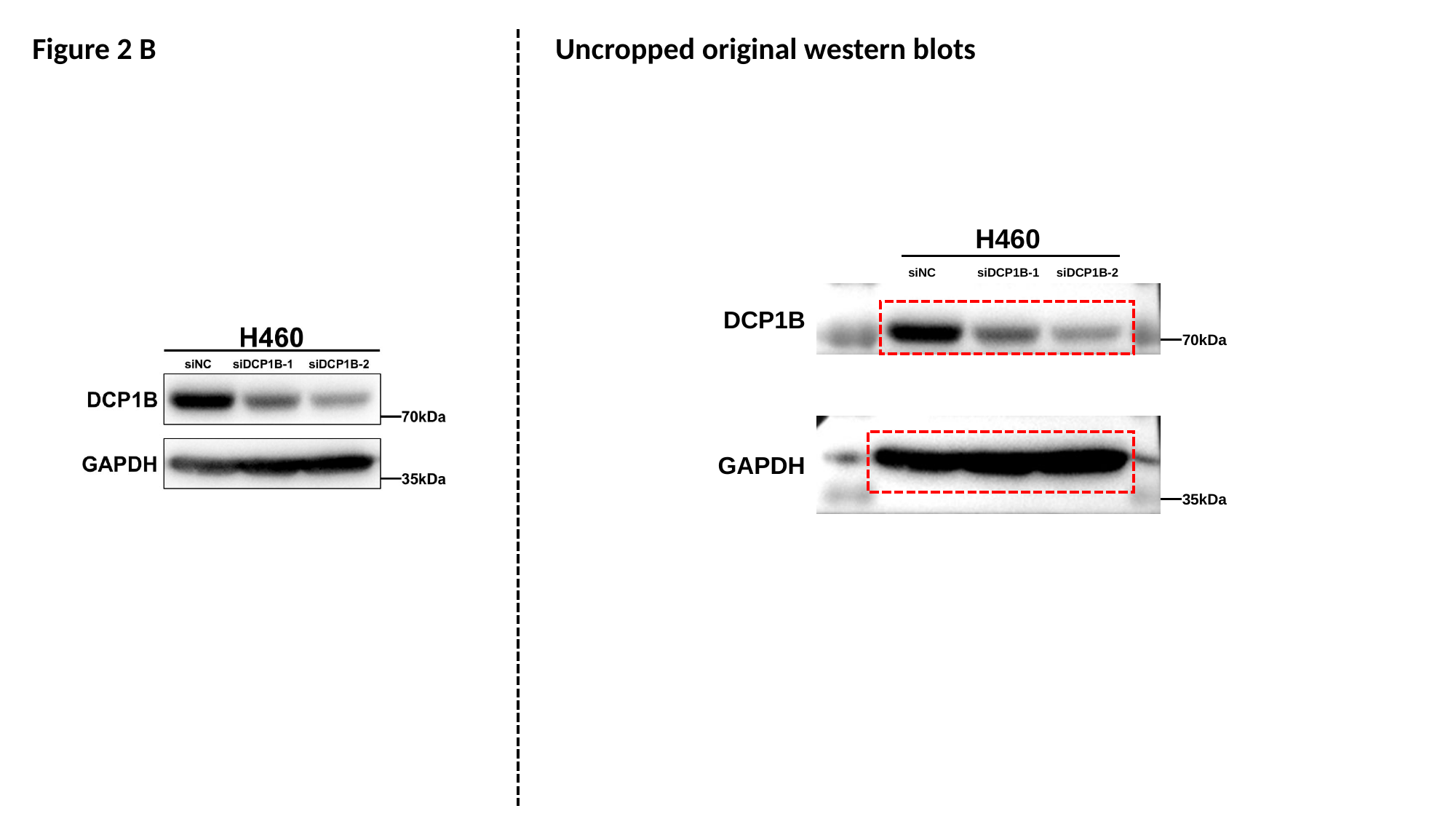

Figure 2 B
Uncropped original western blots
H460
 siNC siDCP1B-1 siDCP1B-2
DCP1B
70kDa
GAPDH
35kDa

## Slide 11
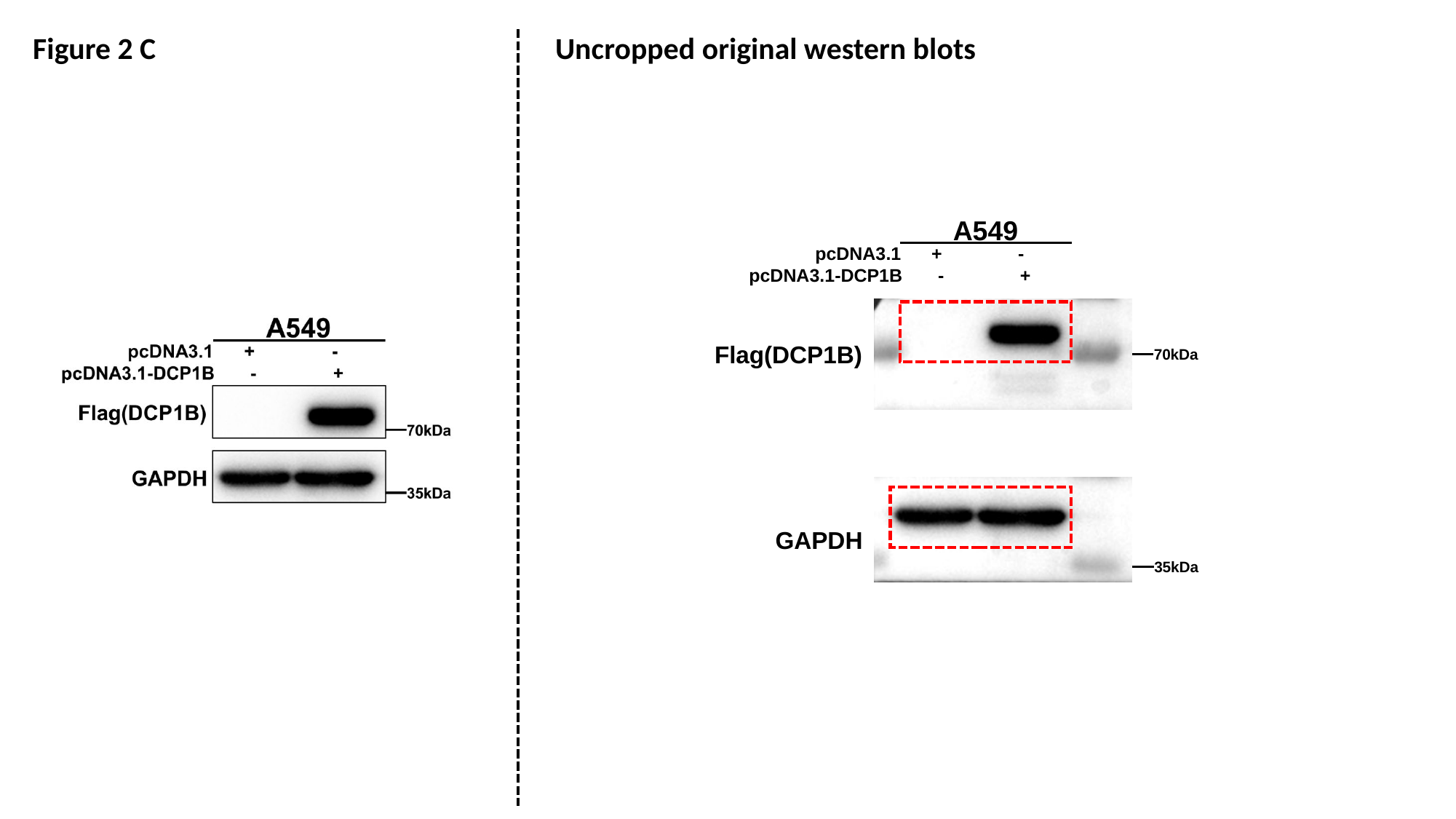

Figure 2 C
Uncropped original western blots
A549
 pcDNA3.1 + -
pcDNA3.1-DCP1B - +
Flag(DCP1B)
70kDa
GAPDH
35kDa

## Slide 12
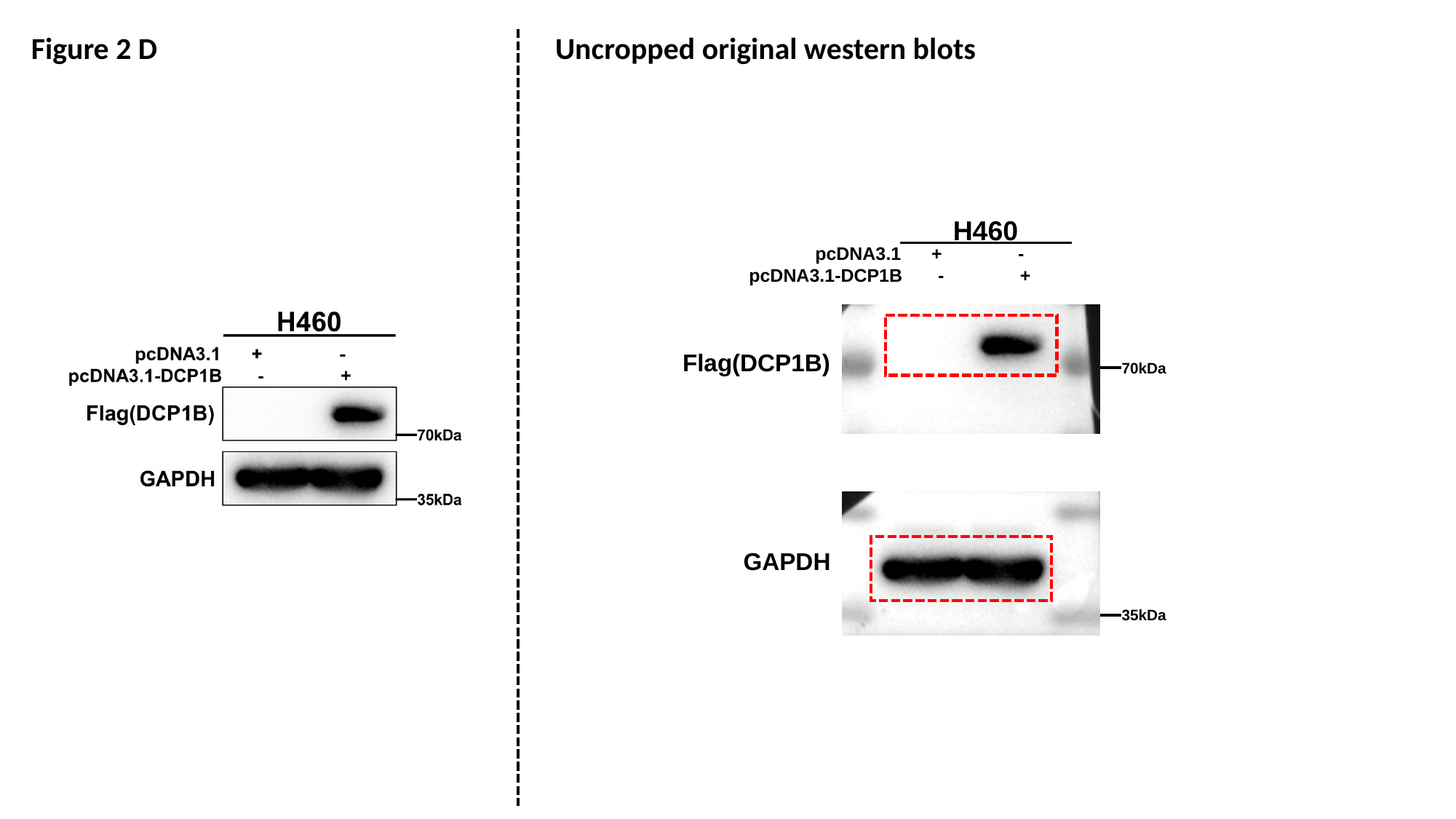

Figure 2 D
Uncropped original western blots
H460
 pcDNA3.1 + -
pcDNA3.1-DCP1B - +
Flag(DCP1B)
70kDa
GAPDH
35kDa

## Slide 13
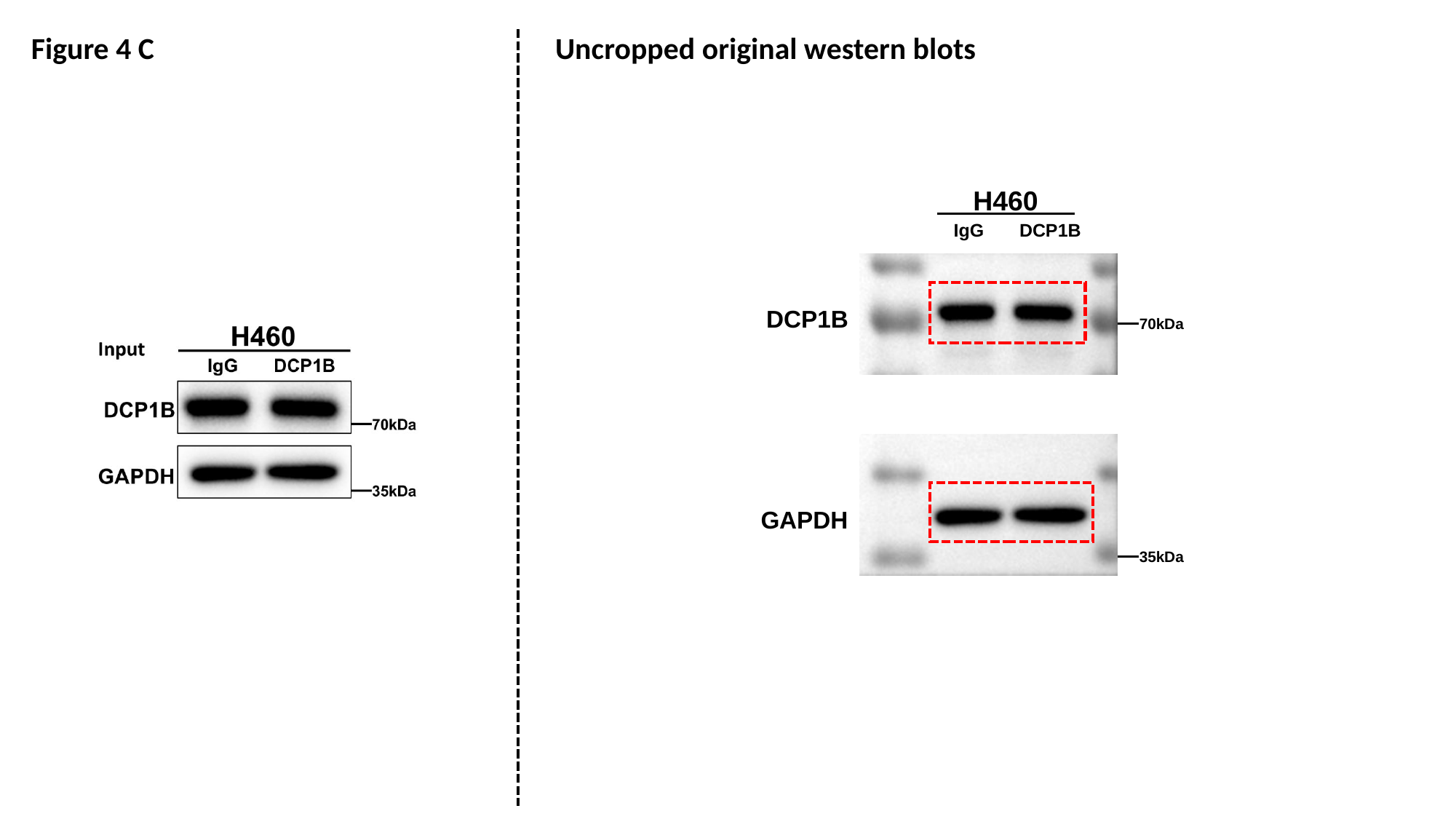

Figure 4 C
Uncropped original western blots
H460
 IgG DCP1B
DCP1B
70kDa
GAPDH
35kDa

## Slide 14
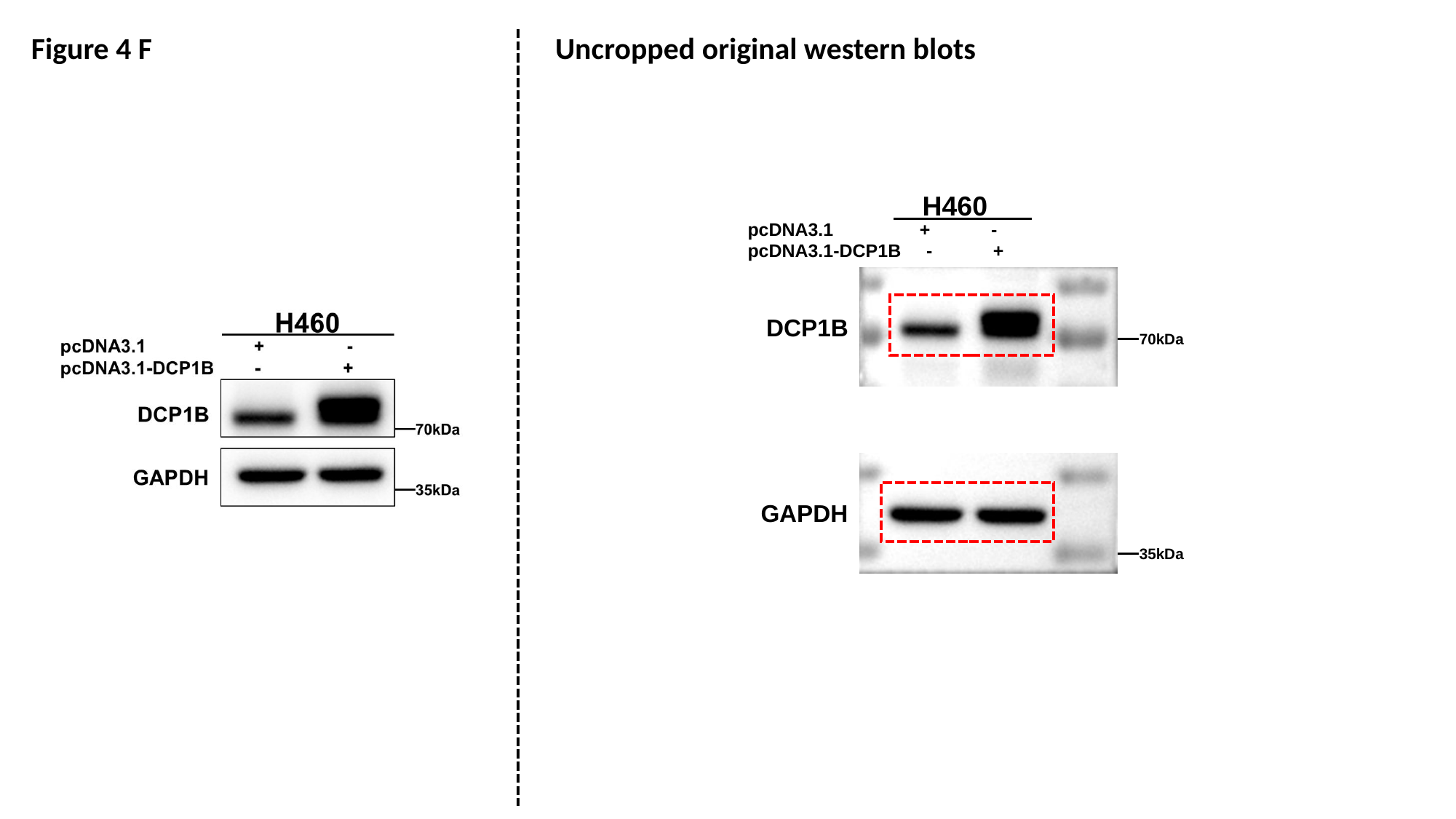

Figure 4 F
Uncropped original western blots
H460
pcDNA3.1 + -
pcDNA3.1-DCP1B - +
DCP1B
70kDa
GAPDH
35kDa

## Slide 15
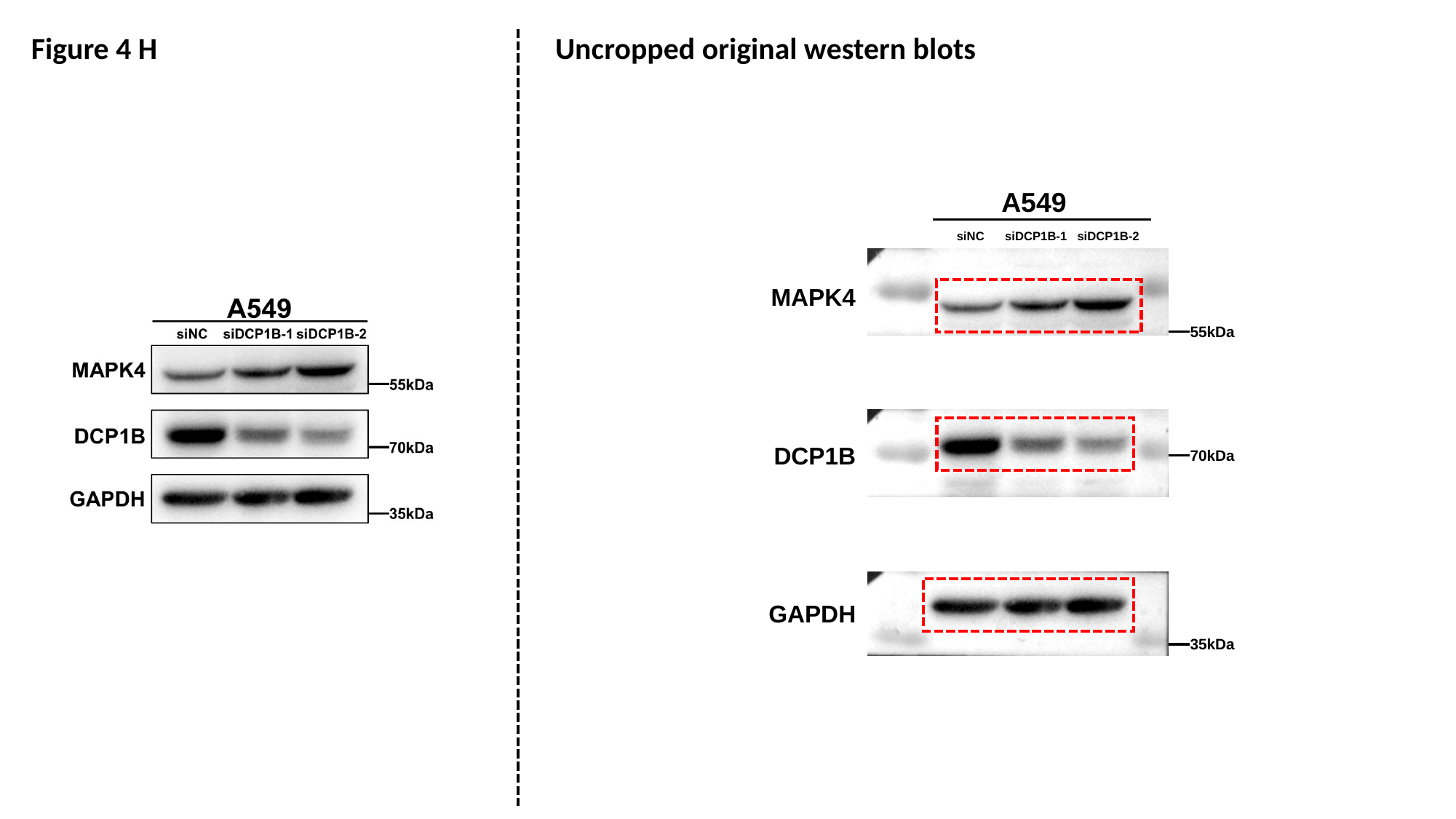

Figure 4 H
Uncropped original western blots
A549
 siNC siDCP1B-1 siDCP1B-2
MAPK4
55kDa
DCP1B
70kDa
GAPDH
35kDa

## Slide 16
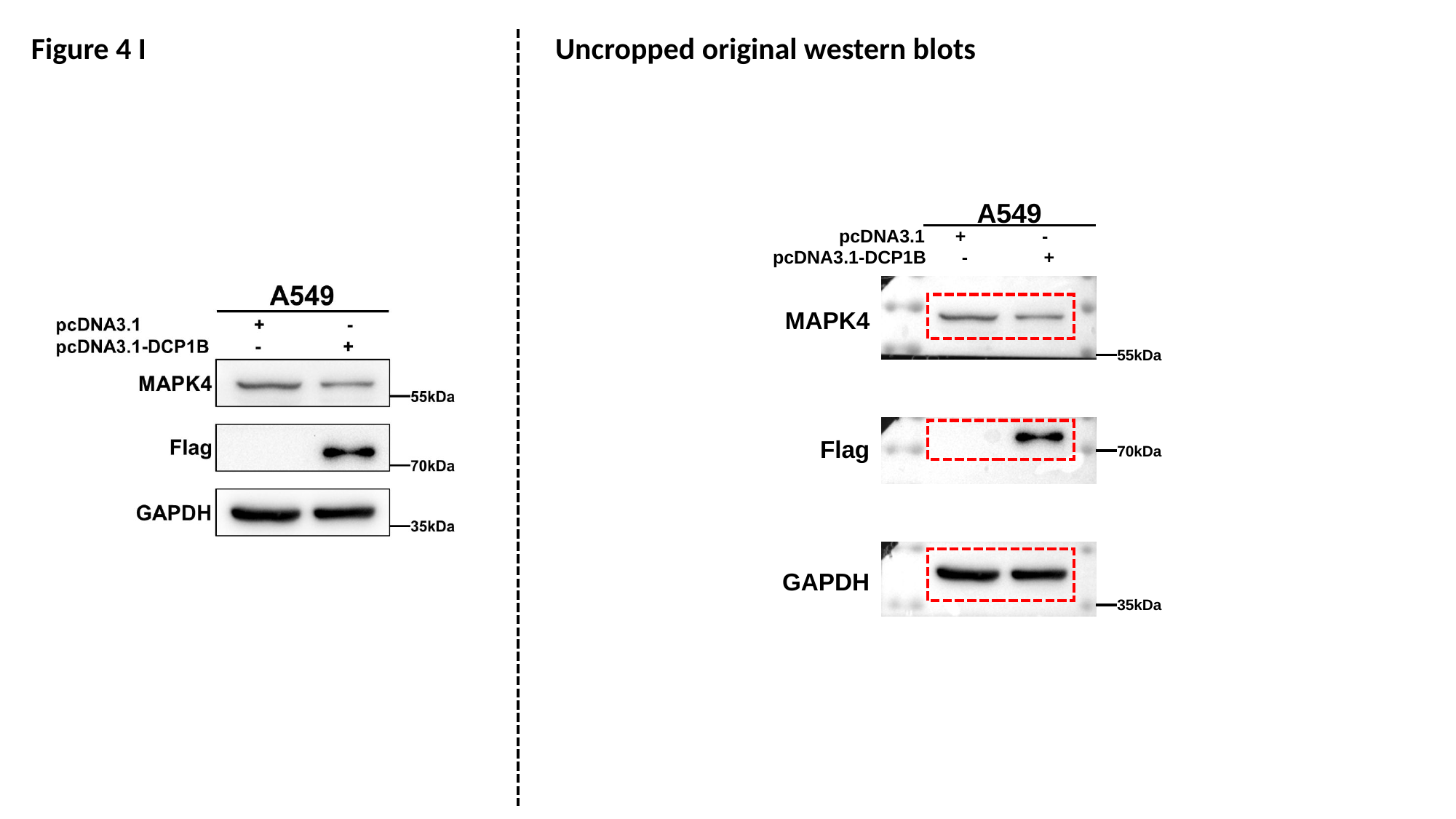

Figure 4 I
Uncropped original western blots
A549
 pcDNA3.1 + -
pcDNA3.1-DCP1B - +
MAPK4
55kDa
Flag
70kDa
GAPDH
35kDa

## Slide 17
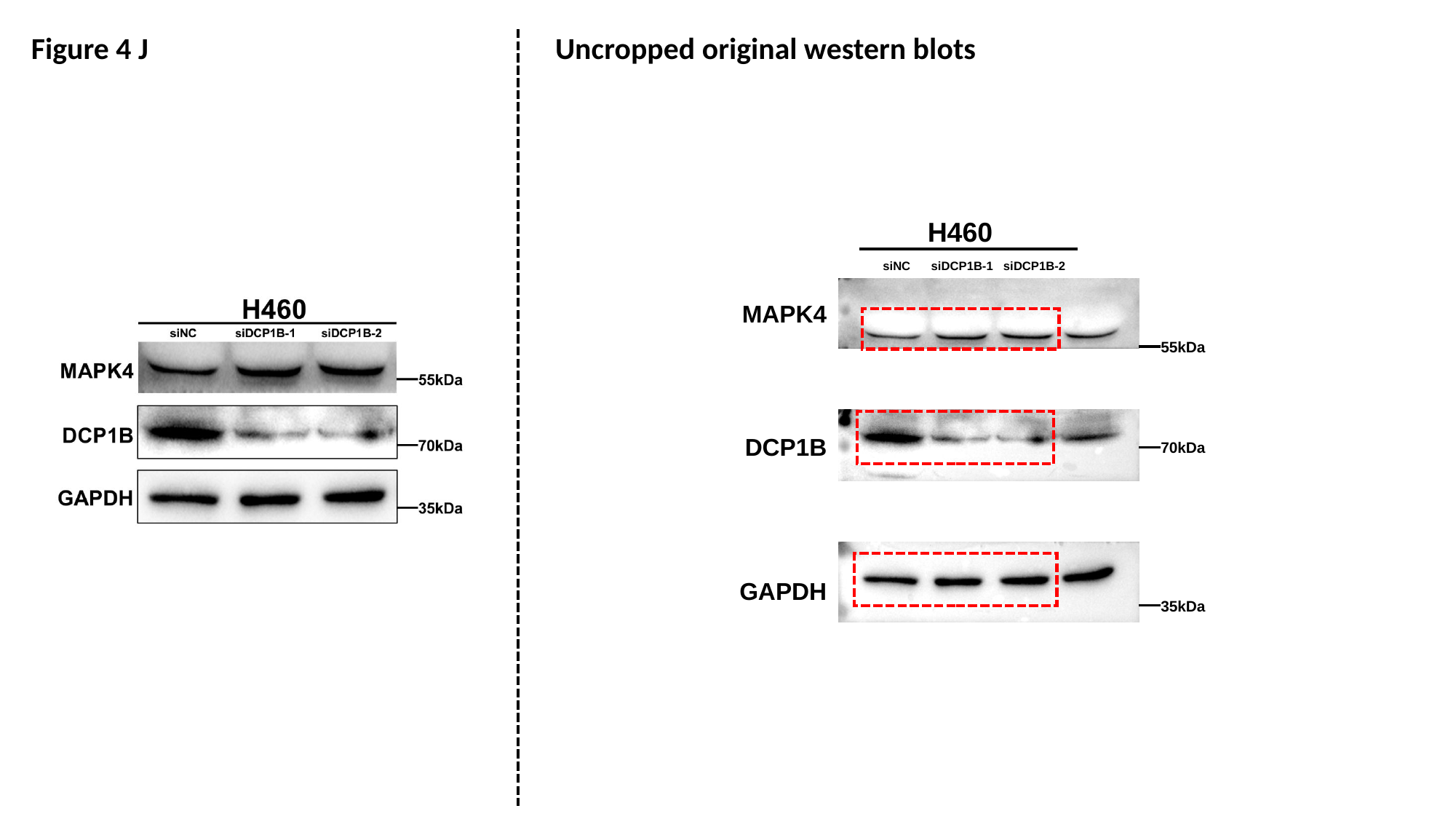

Figure 4 J
Uncropped original western blots
H460
 siNC siDCP1B-1 siDCP1B-2
MAPK4
55kDa
DCP1B
70kDa
GAPDH
35kDa

## Slide 18
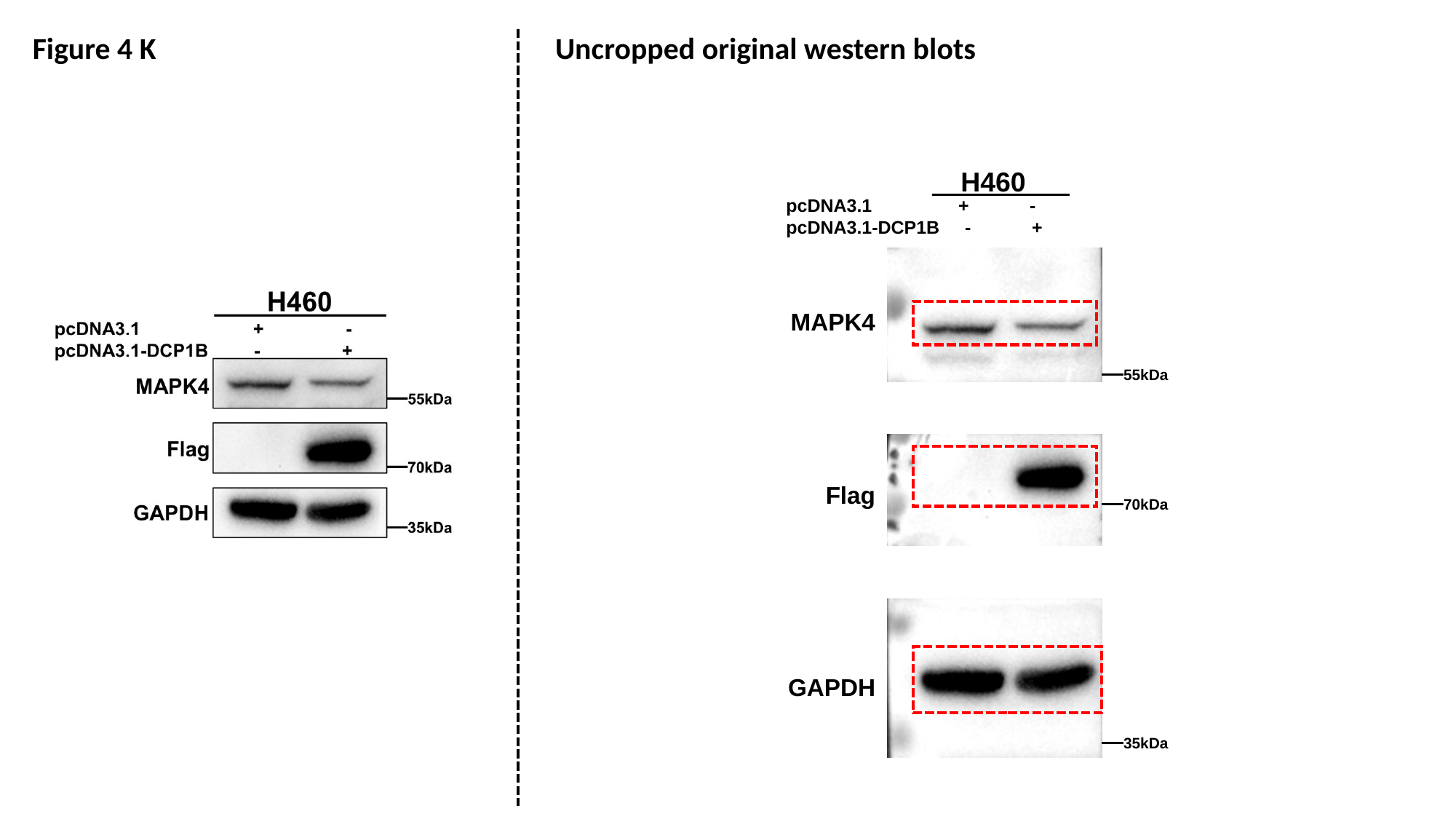

Figure 4 K
Uncropped original western blots
H460
pcDNA3.1 + -
pcDNA3.1-DCP1B - +
MAPK4
55kDa
Flag
70kDa
GAPDH
35kDa

## Slide 19
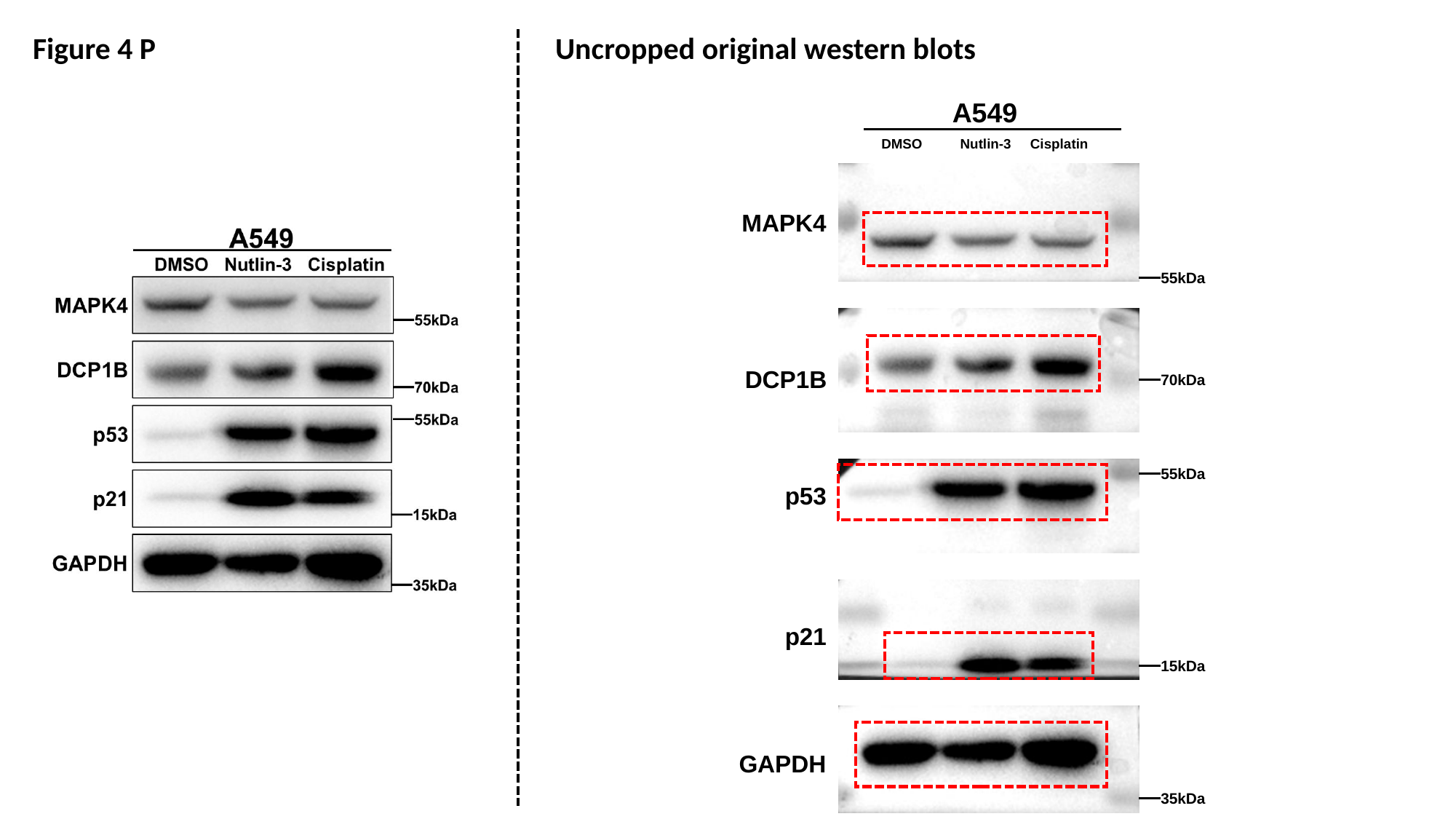

Figure 4 P
Uncropped original western blots
A549
DMSO Nutlin-3 Cisplatin
MAPK4
55kDa
DCP1B
70kDa
55kDa
p53
p21
15kDa
GAPDH
35kDa

## Slide 20
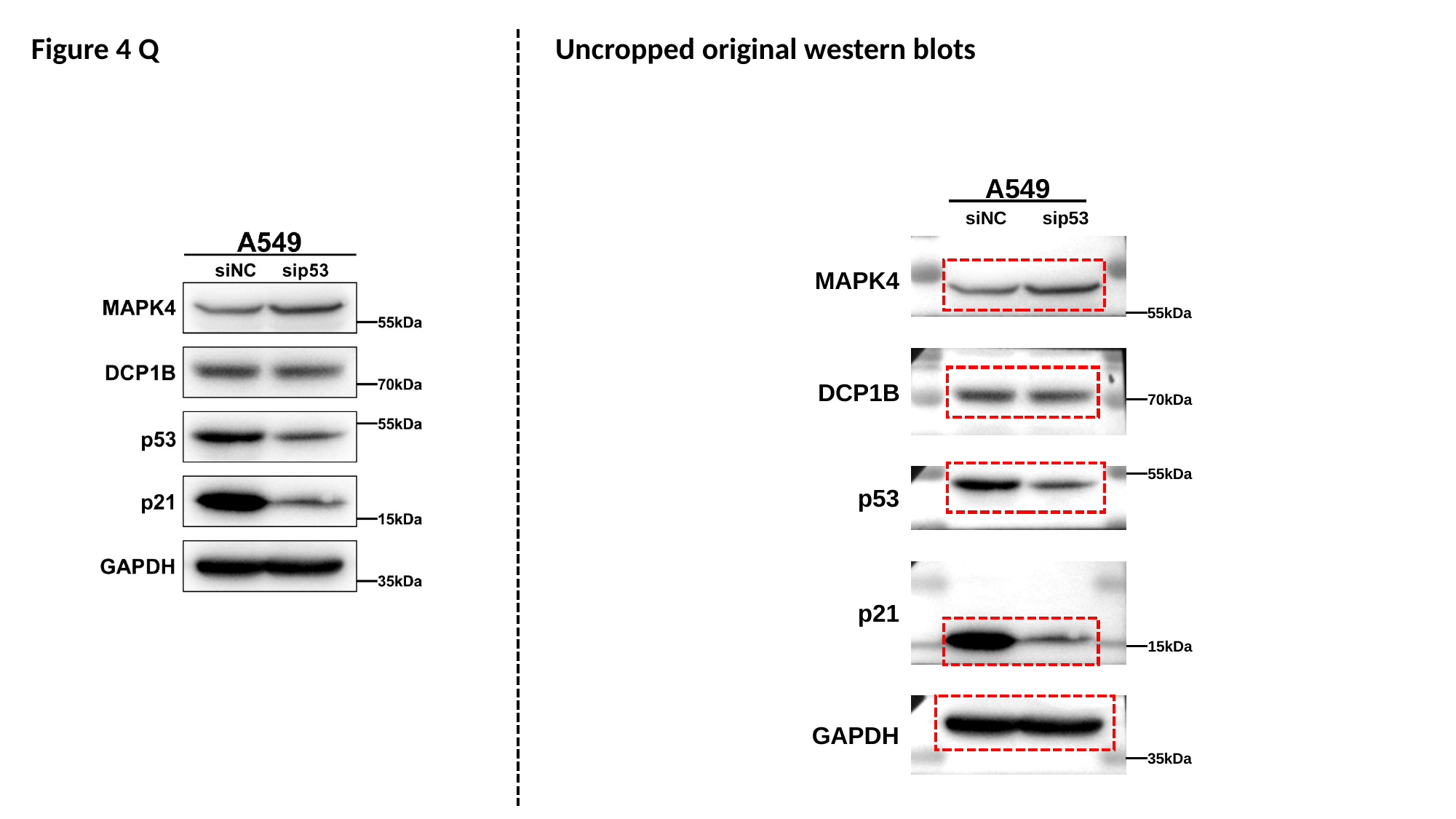

Figure 4 Q
Uncropped original western blots
A549
 siNC sip53
MAPK4
55kDa
DCP1B
70kDa
55kDa
p53
p21
15kDa
GAPDH
35kDa

## Slide 21
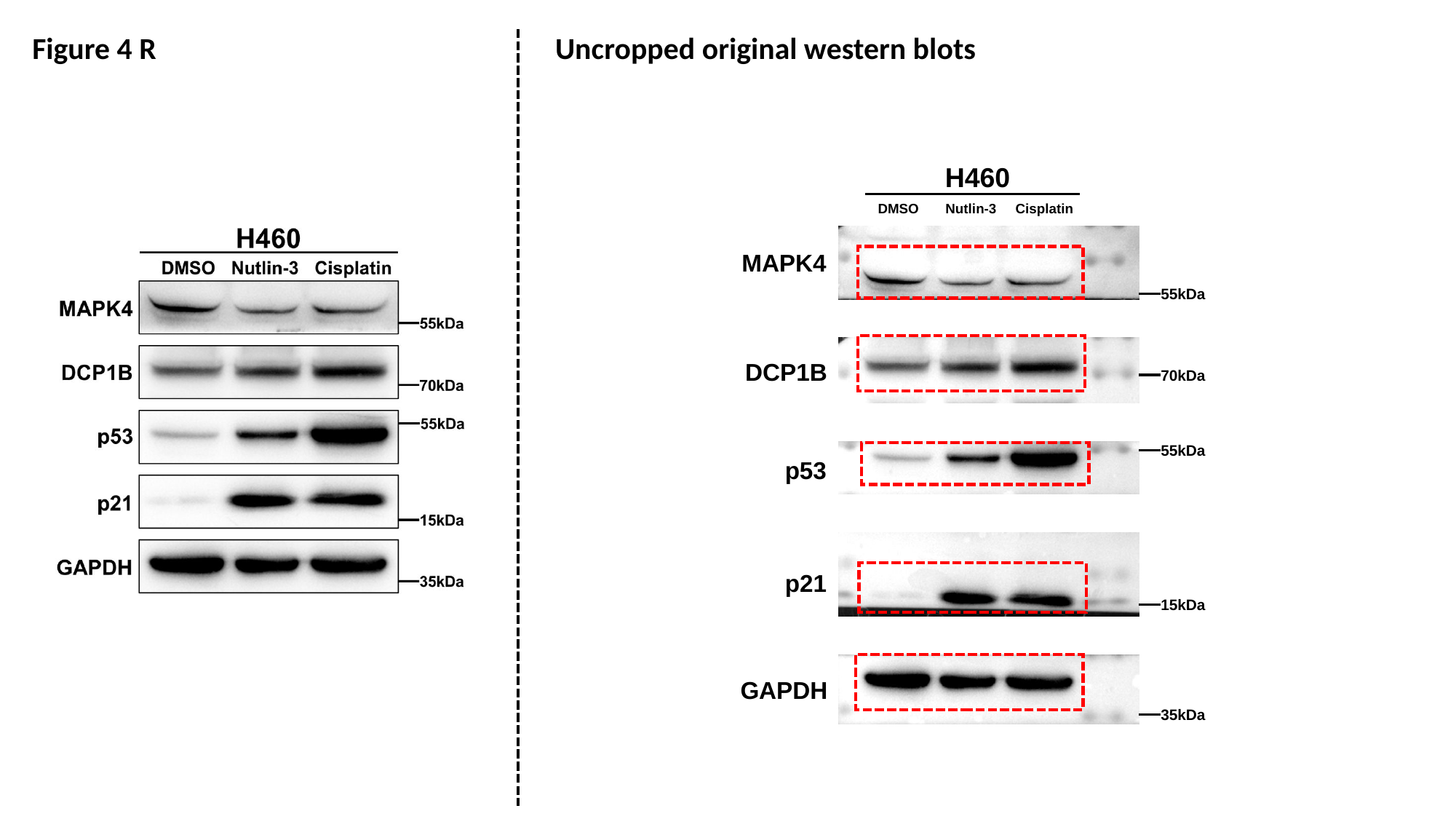

Figure 4 R
Uncropped original western blots
H460
 DMSO Nutlin-3 Cisplatin
MAPK4
55kDa
DCP1B
70kDa
55kDa
p53
p21
15kDa
GAPDH
35kDa

## Slide 22
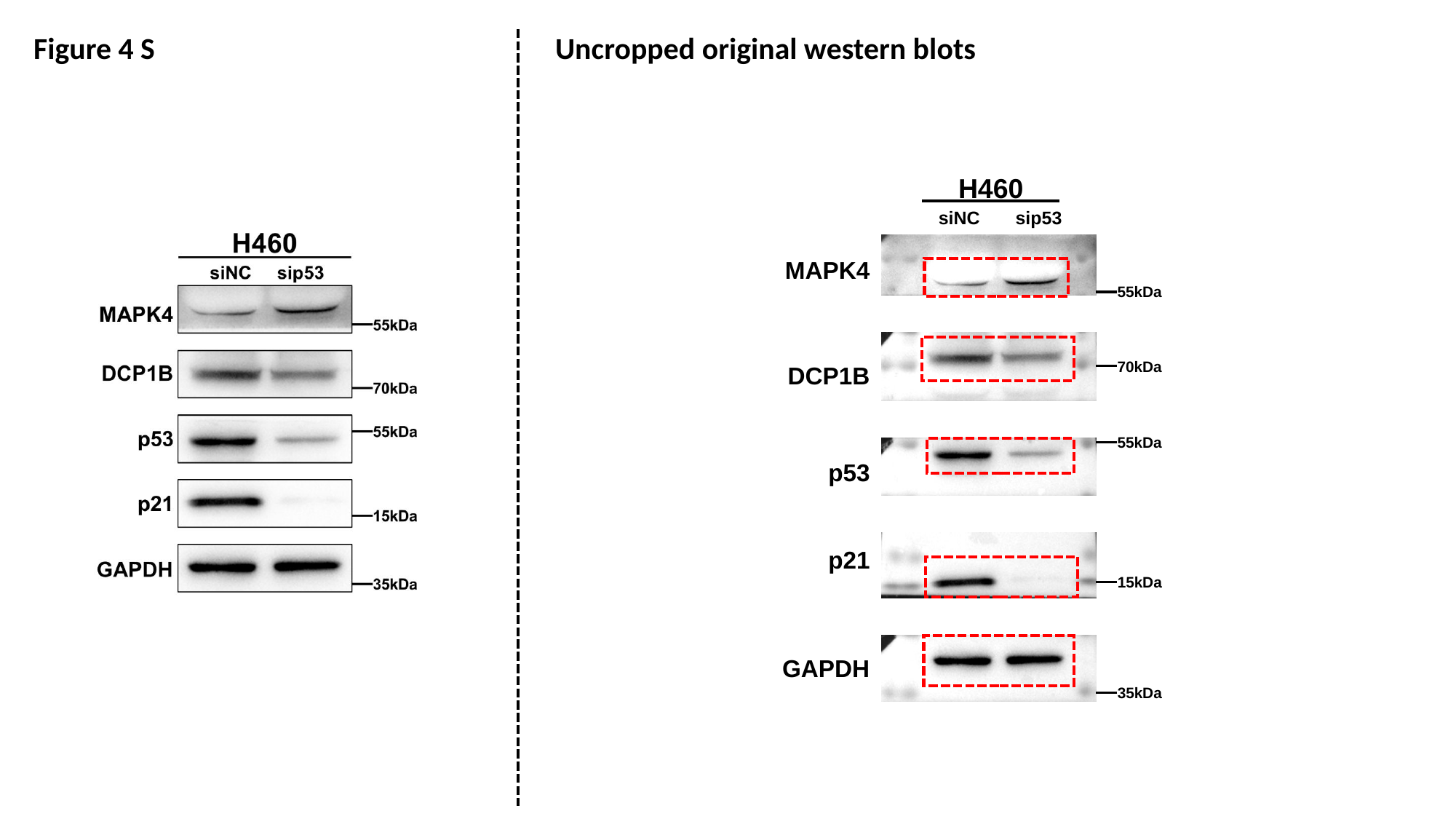

Figure 4 S
Uncropped original western blots
H460
 siNC sip53
MAPK4
55kDa
70kDa
DCP1B
55kDa
p53
p21
15kDa
GAPDH
35kDa

## Slide 23
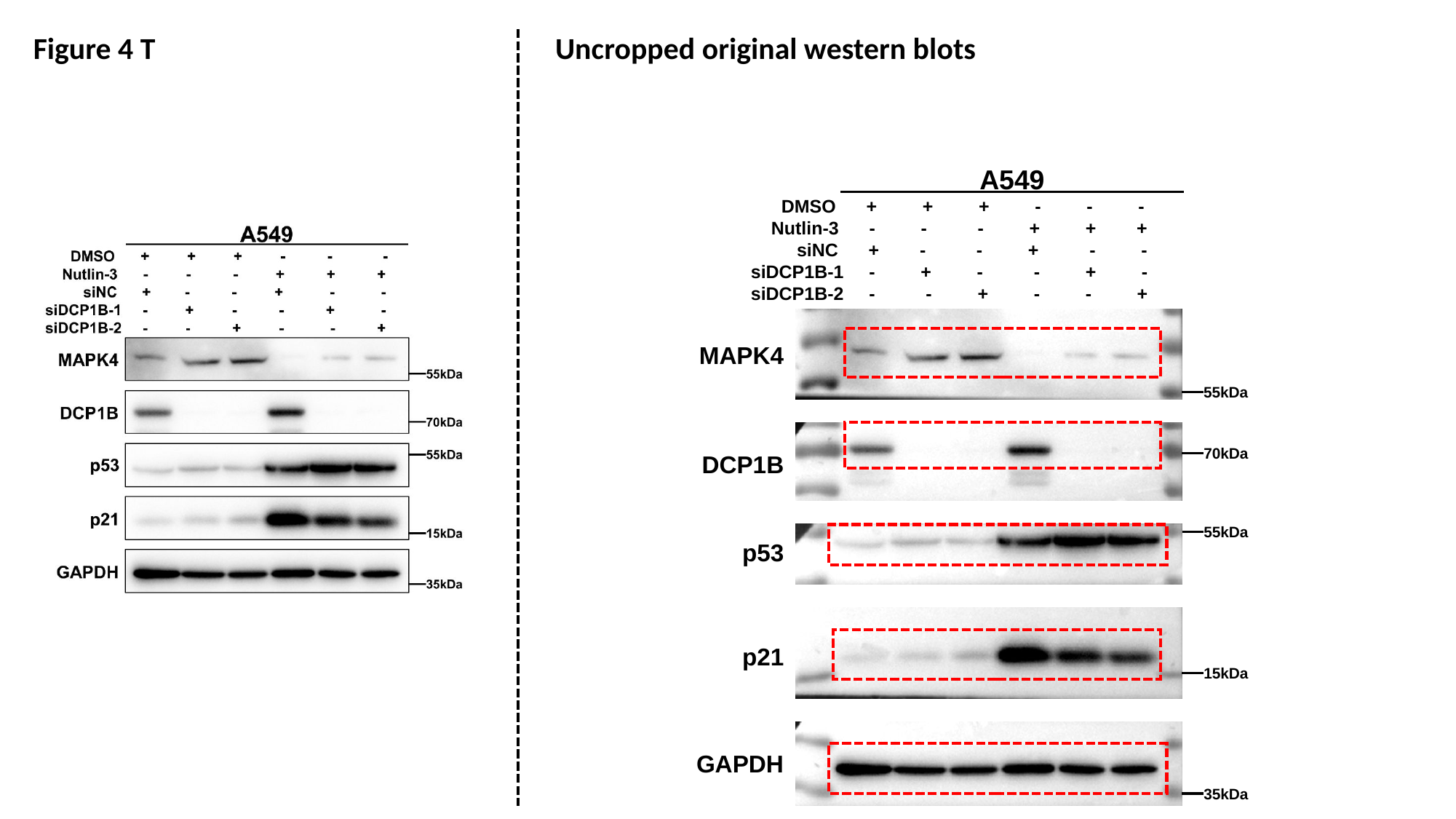

Figure 4 T
Uncropped original western blots
A549
 DMSO + + + - - -
 Nutlin-3 - - - + + +
 siNC + - - + - -
siDCP1B-1 - + - - + -
siDCP1B-2 - - + - - +
MAPK4
55kDa
70kDa
DCP1B
55kDa
p53
p21
15kDa
GAPDH
35kDa

## Slide 24
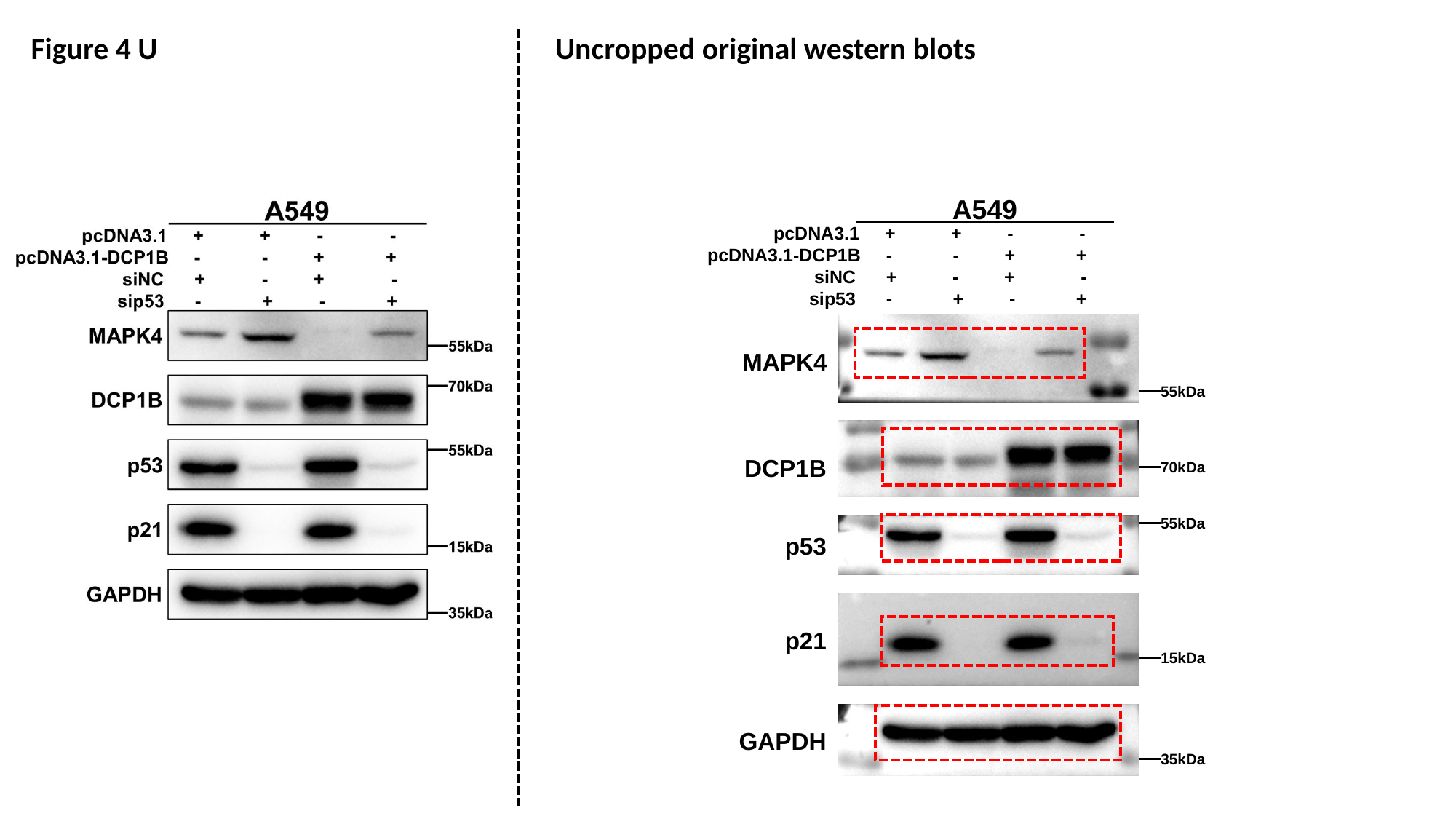

Figure 4 U
Uncropped original western blots
A549
 pcDNA3.1 + + - -
pcDNA3.1-DCP1B - - + +
 siNC + - + -
 sip53 - + - +
MAPK4
55kDa
DCP1B
70kDa
55kDa
p53
p21
15kDa
GAPDH
35kDa

## Slide 25
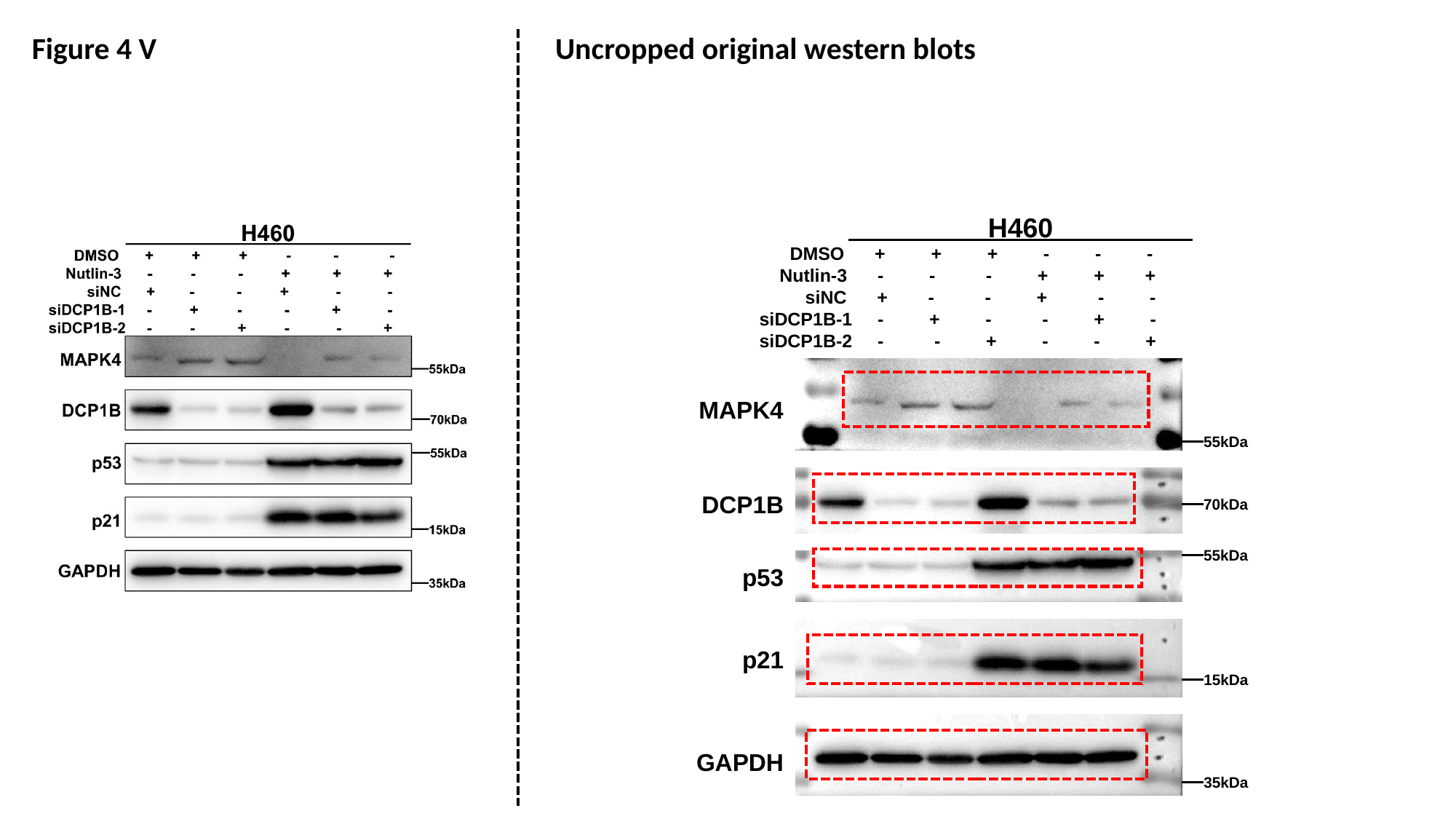

Figure 4 V
Uncropped original western blots
H460
 DMSO + + + - - -
 Nutlin-3 - - - + + +
 siNC + - - + - -
siDCP1B-1 - + - - + -
siDCP1B-2 - - + - - +
MAPK4
55kDa
DCP1B
70kDa
55kDa
p53
p21
15kDa
GAPDH
35kDa

## Slide 26
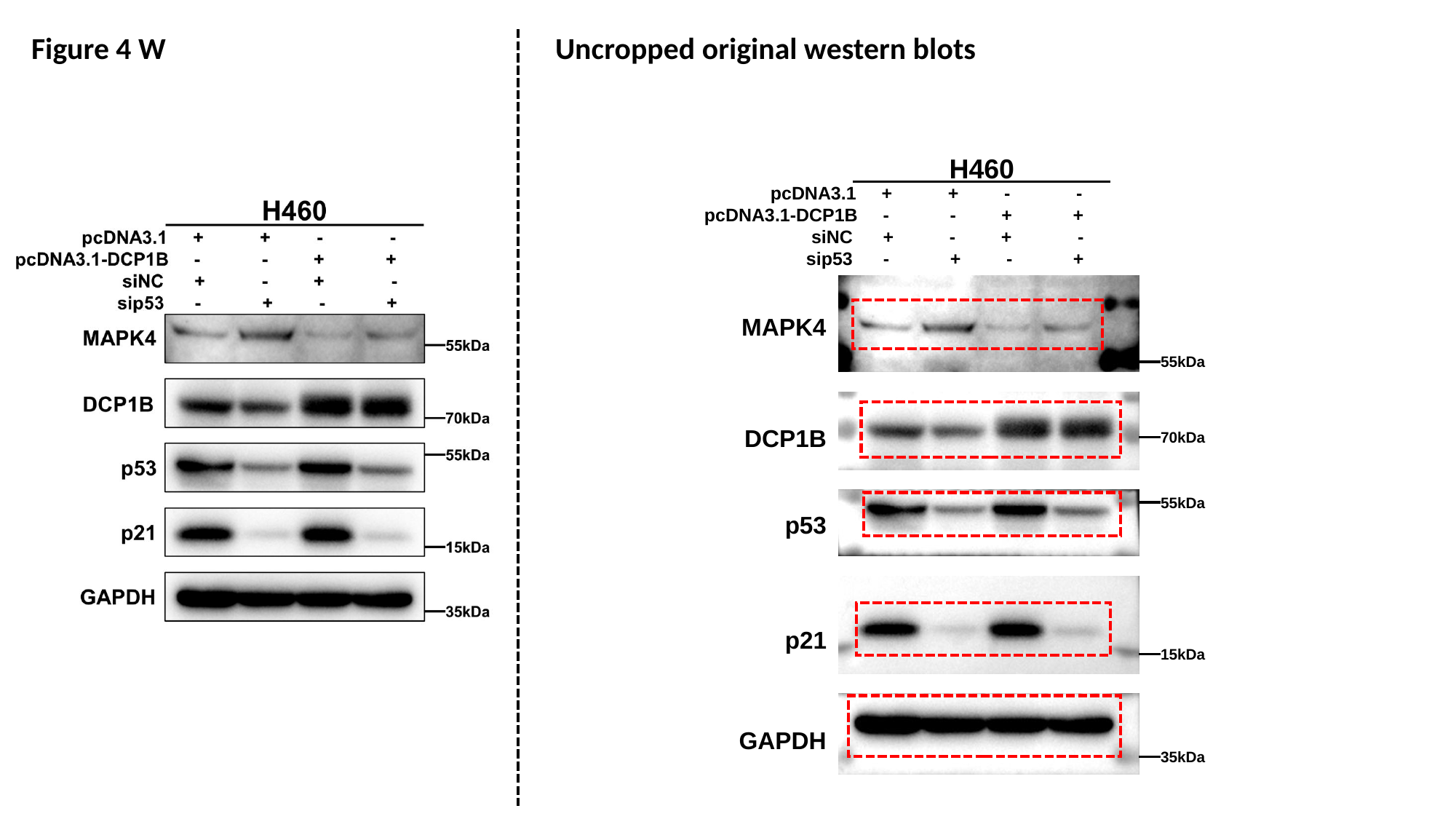

Figure 4 W
Uncropped original western blots
H460
 pcDNA3.1 + + - -
pcDNA3.1-DCP1B - - + +
 siNC + - + -
 sip53 - + - +
MAPK4
55kDa
DCP1B
70kDa
55kDa
p53
p21
15kDa
GAPDH
35kDa

## Slide 27
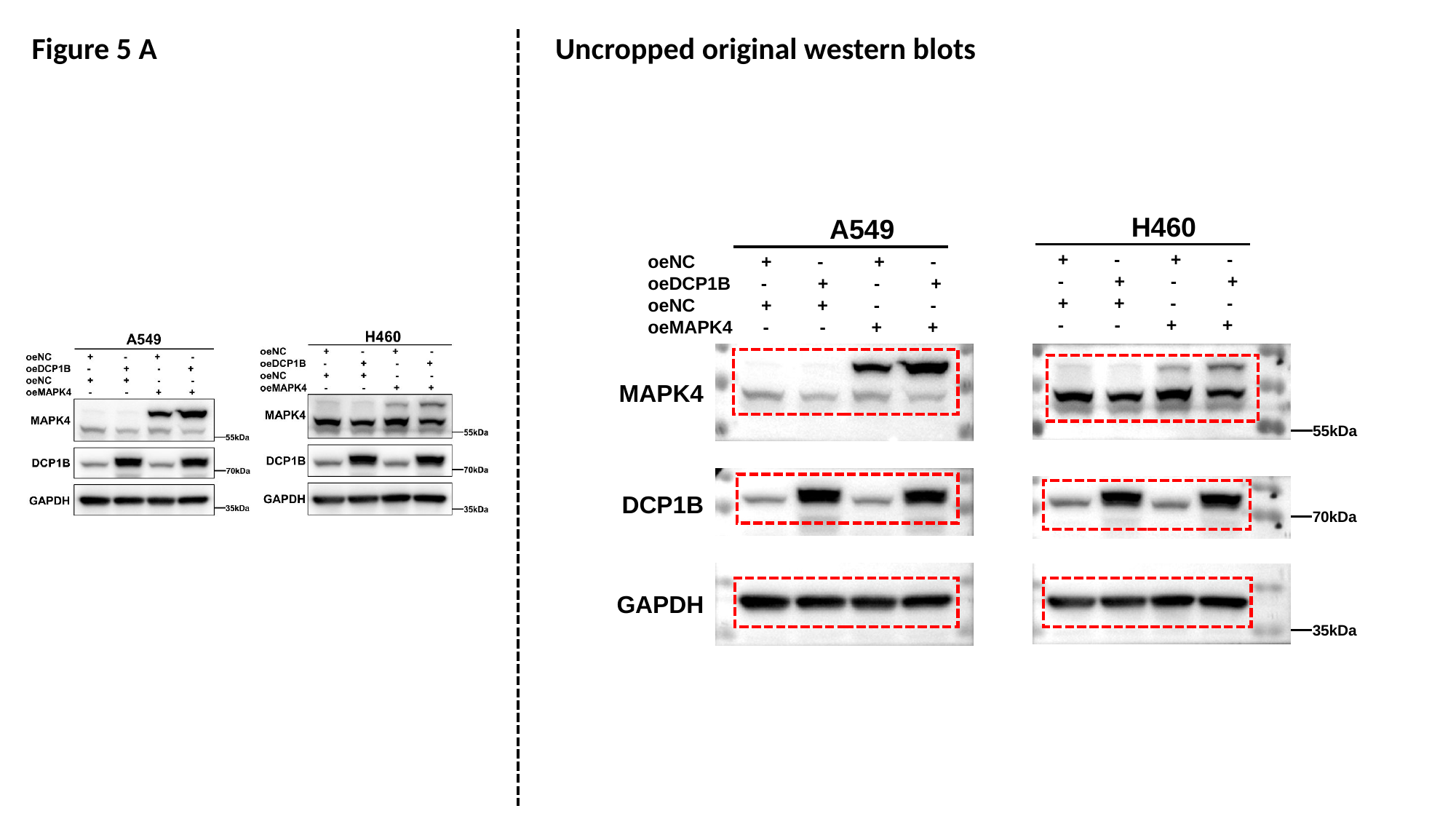

Figure 5 A
Uncropped original western blots
H460
A549
+ - + -
- + - +
+ + - -
- - + +
oeNC + - + -
oeDCP1B - + - +
oeNC + + - -
oeMAPK4 - - + +
MAPK4
55kDa
DCP1B
70kDa
GAPDH
35kDa

## Slide 28
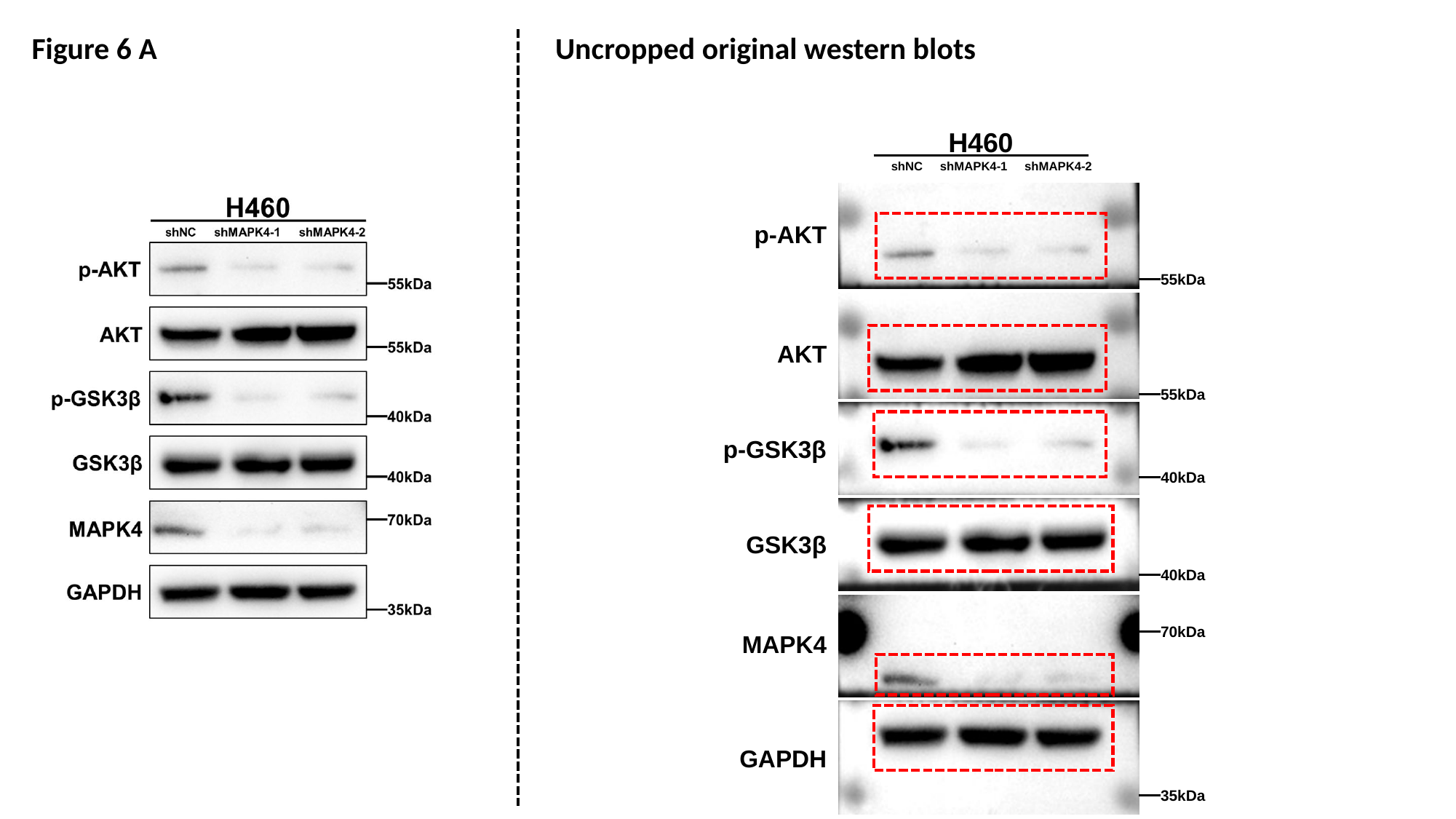

Figure 6 A
Uncropped original western blots
H460
 shNC shMAPK4-1 shMAPK4-2
p-AKT
55kDa
AKT
55kDa
p-GSK3β
40kDa
GSK3β
40kDa
70kDa
MAPK4
GAPDH
35kDa

## Slide 29
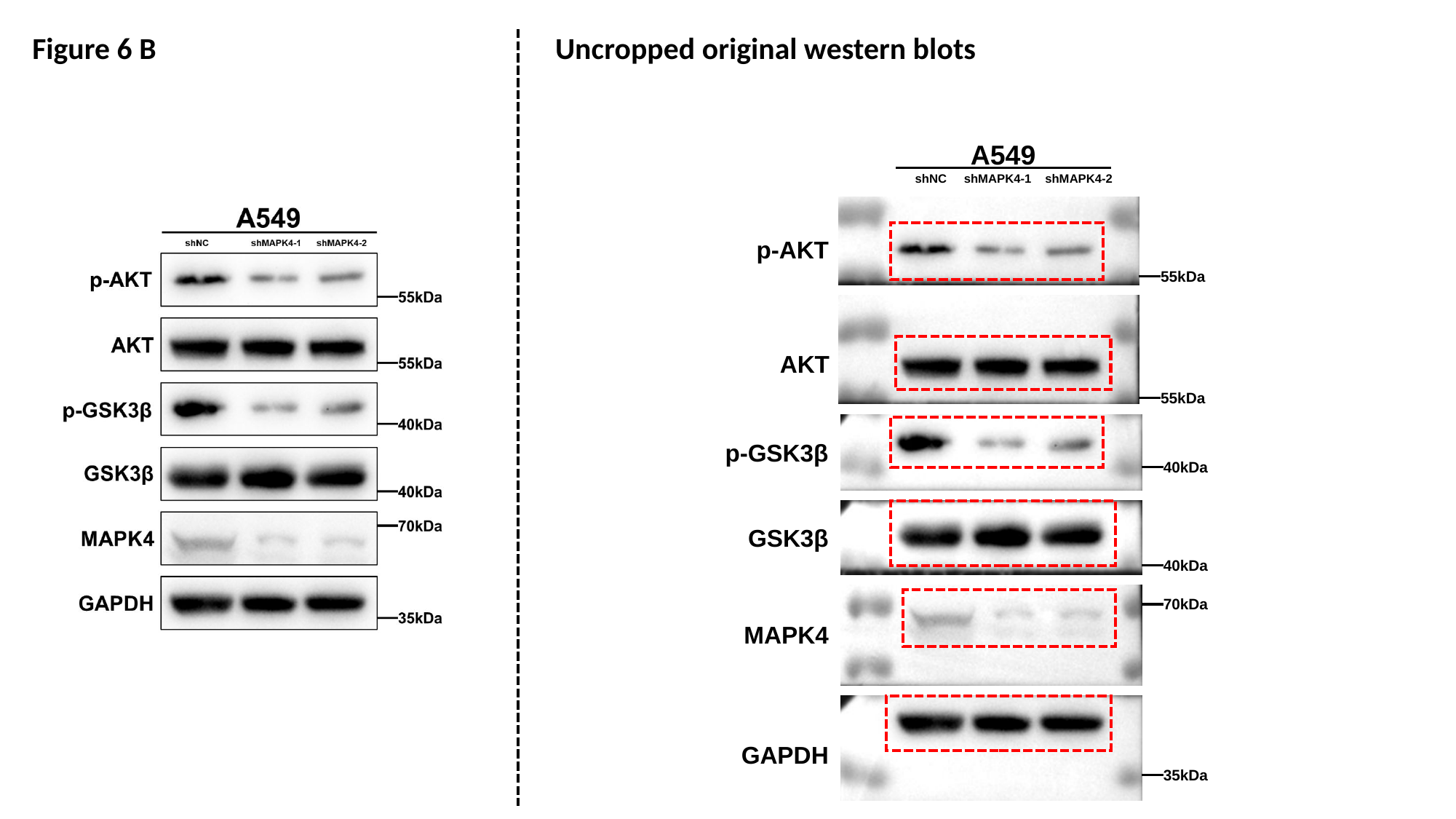

Figure 6 B
Uncropped original western blots
A549
 shNC shMAPK4-1 shMAPK4-2
p-AKT
55kDa
AKT
55kDa
p-GSK3β
40kDa
GSK3β
40kDa
70kDa
MAPK4
GAPDH
35kDa

## Slide 30
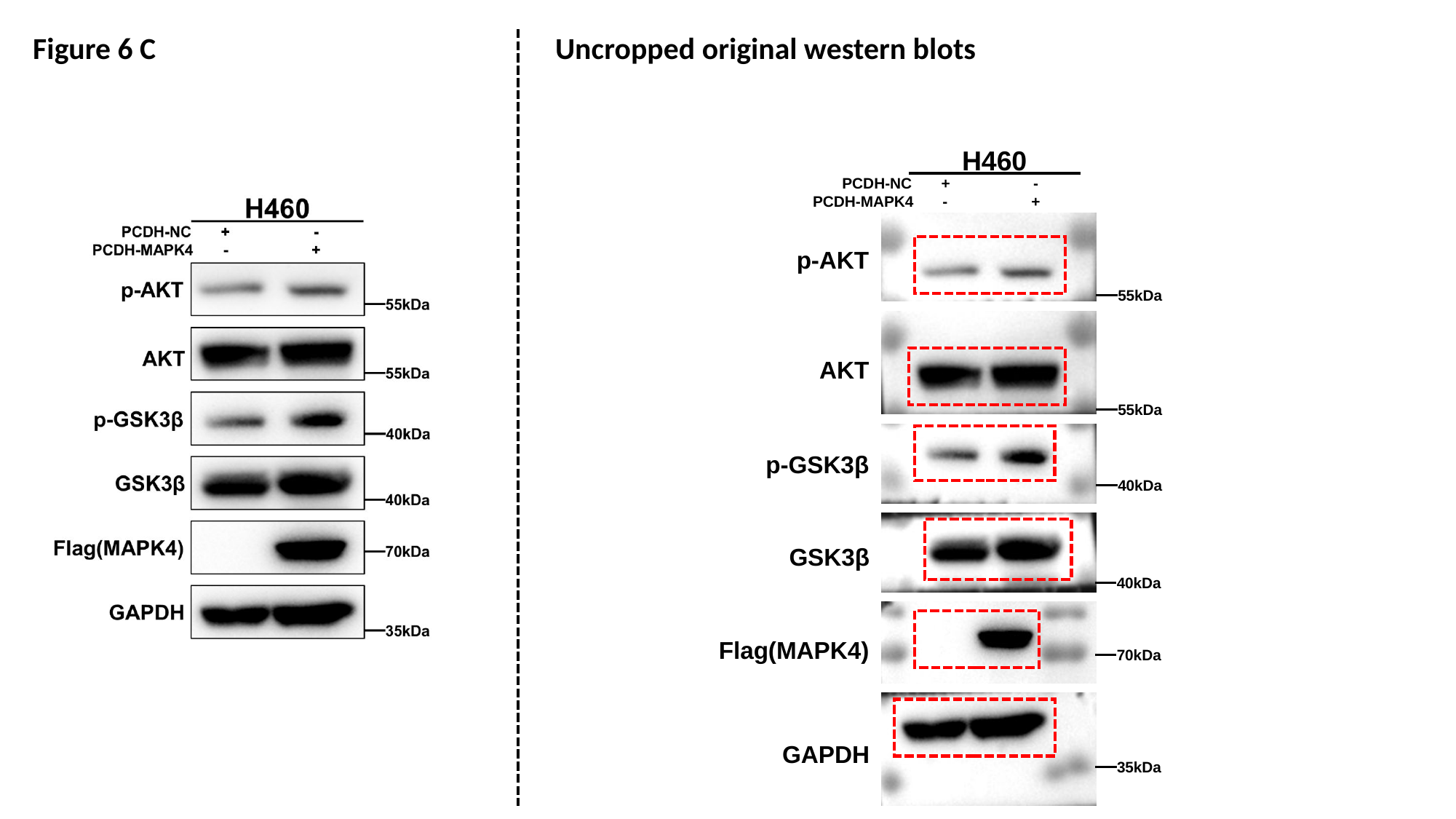

Figure 6 C
Uncropped original western blots
H460
 PCDH-NC + -
PCDH-MAPK4 - +
p-AKT
55kDa
AKT
55kDa
p-GSK3β
40kDa
GSK3β
40kDa
Flag(MAPK4)
70kDa
GAPDH
35kDa

## Slide 31
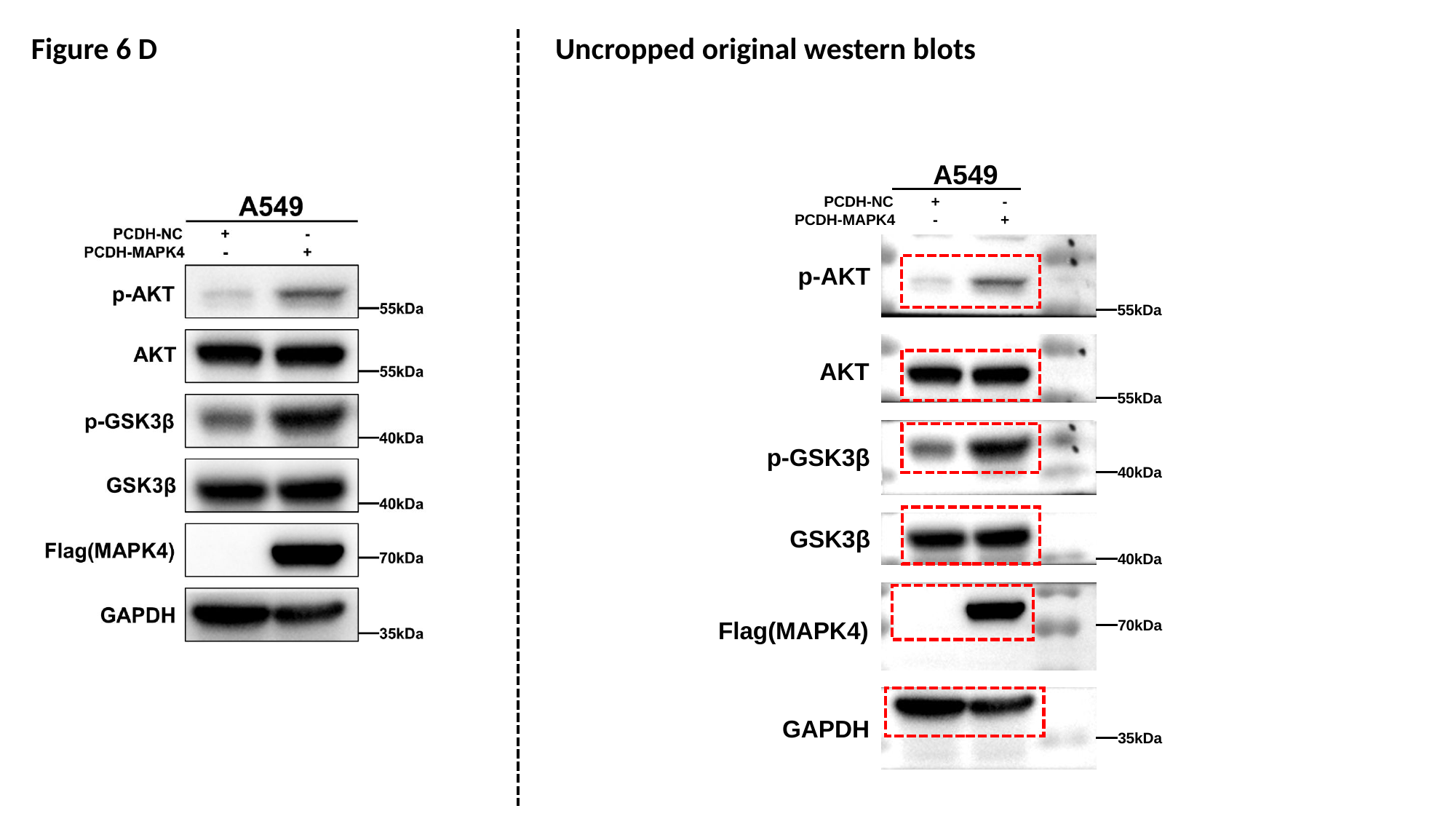

Figure 6 D
Uncropped original western blots
A549
 PCDH-NC + -
PCDH-MAPK4 - +
p-AKT
55kDa
AKT
55kDa
p-GSK3β
40kDa
GSK3β
40kDa
Flag(MAPK4)
70kDa
GAPDH
35kDa

## Slide 32
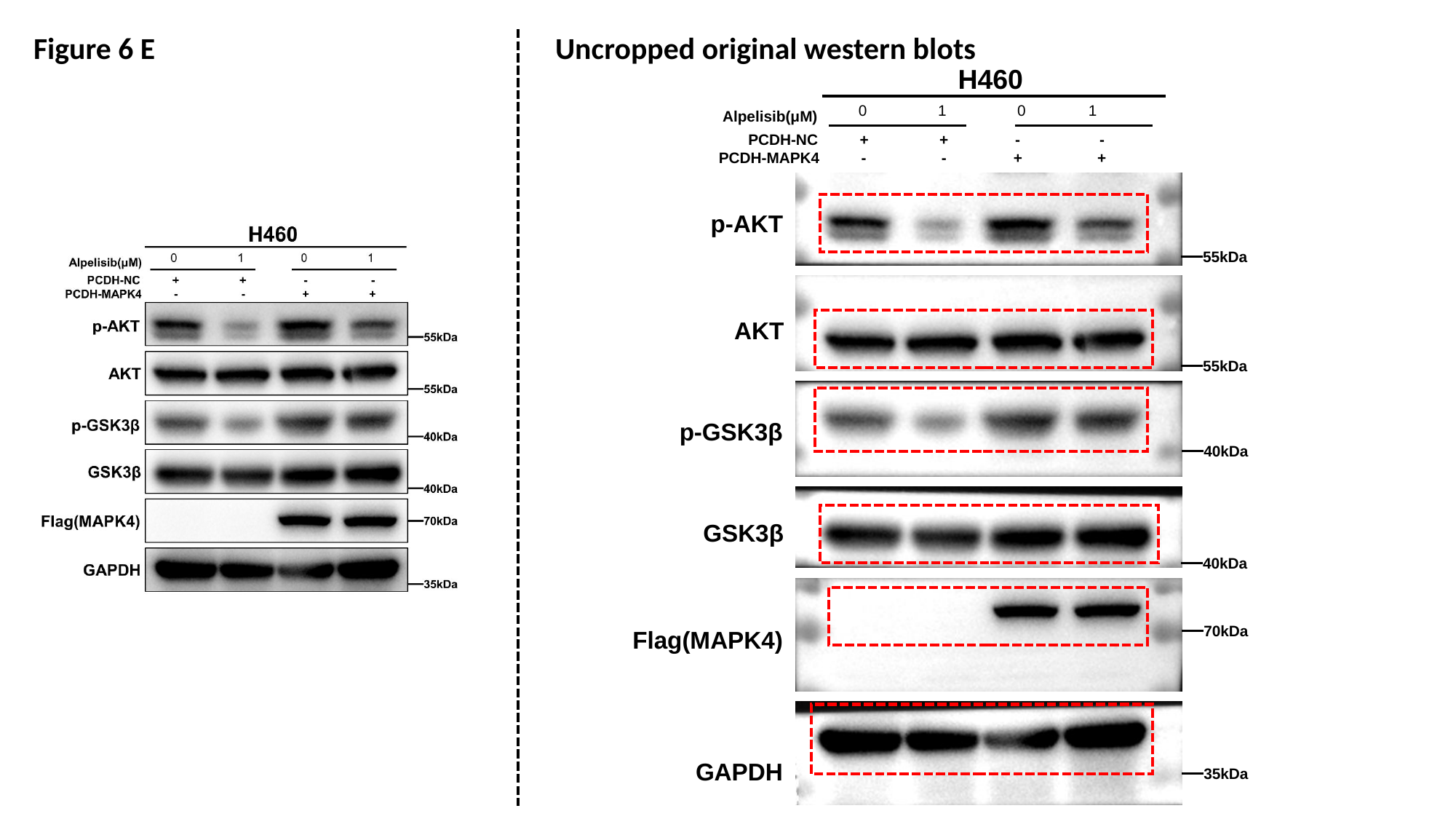

Figure 6 E
Uncropped original western blots
H460
0 1 0 1
Alpelisib(μM)
 PCDH-NC + + - -
PCDH-MAPK4 - - + +
p-AKT
55kDa
AKT
55kDa
p-GSK3β
40kDa
GSK3β
40kDa
70kDa
Flag(MAPK4)
GAPDH
35kDa

## Slide 33
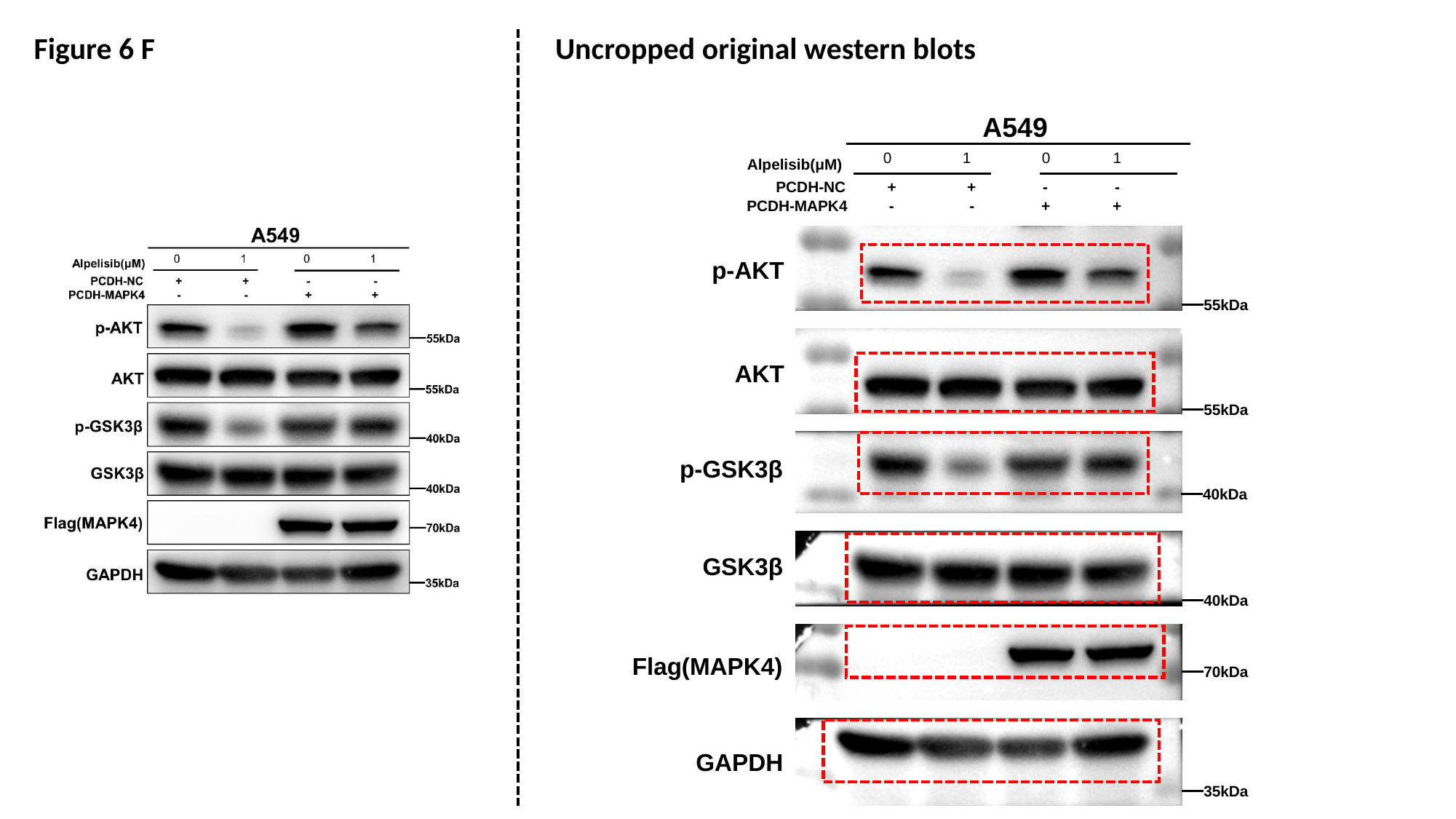

Figure 6 F
Uncropped original western blots
A549
0 1 0 1
Alpelisib(μM)
 PCDH-NC + + - -
PCDH-MAPK4 - - + +
p-AKT
55kDa
AKT
55kDa
p-GSK3β
40kDa
GSK3β
40kDa
Flag(MAPK4)
70kDa
GAPDH
35kDa

## Slide 34
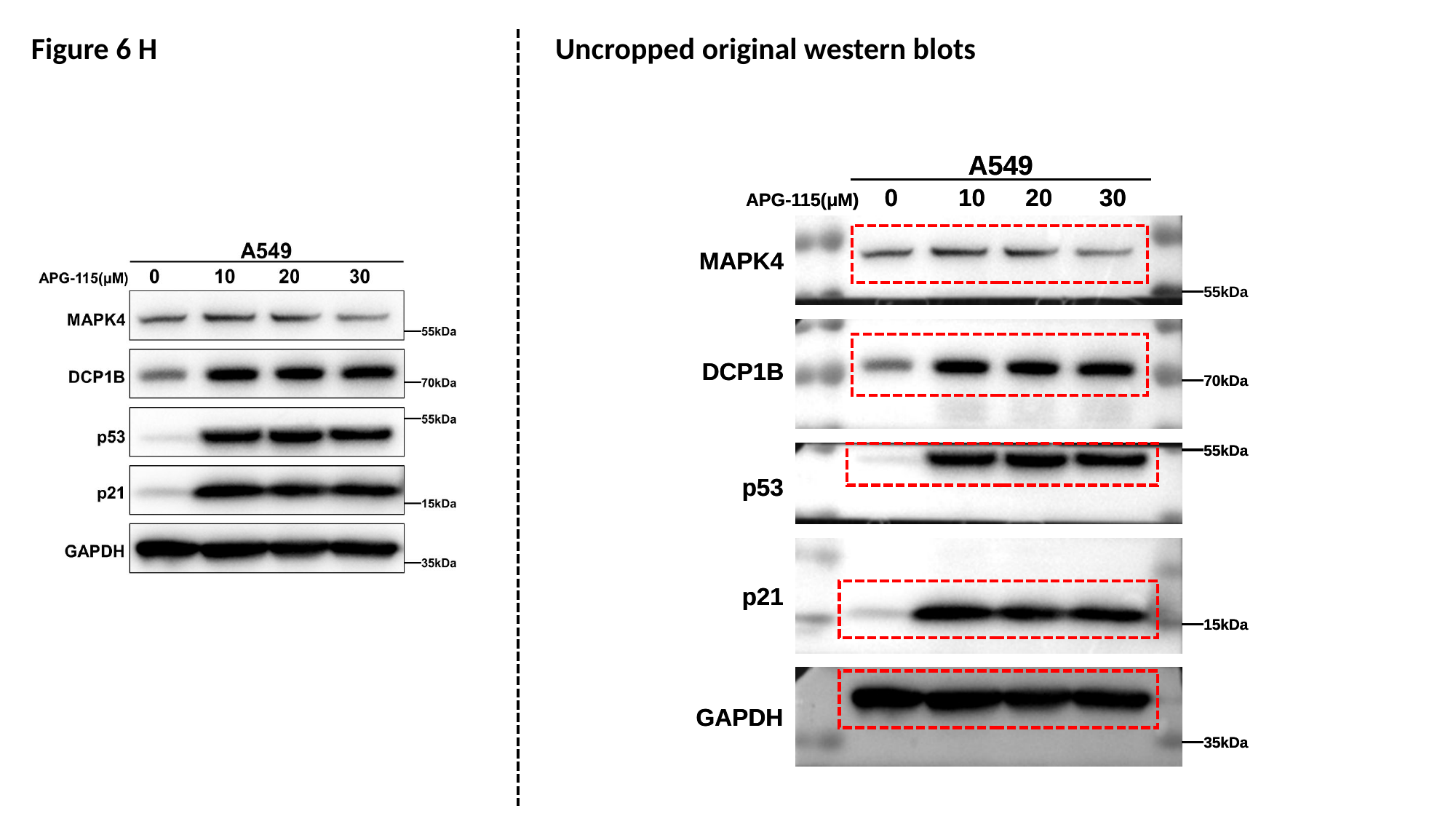

Figure 6 H
Uncropped original western blots
A549
A549
 APG-115(μM) 0 10 20 30
 APG-115(μM) 0 10 20 30
MAPK4
MAPK4
55kDa
DCP1B
DCP1B
70kDa
70kDa
55kDa
55kDa
p53
p53
p21
p21
15kDa
15kDa
GAPDH
GAPDH
35kDa
35kDa

## Slide 35
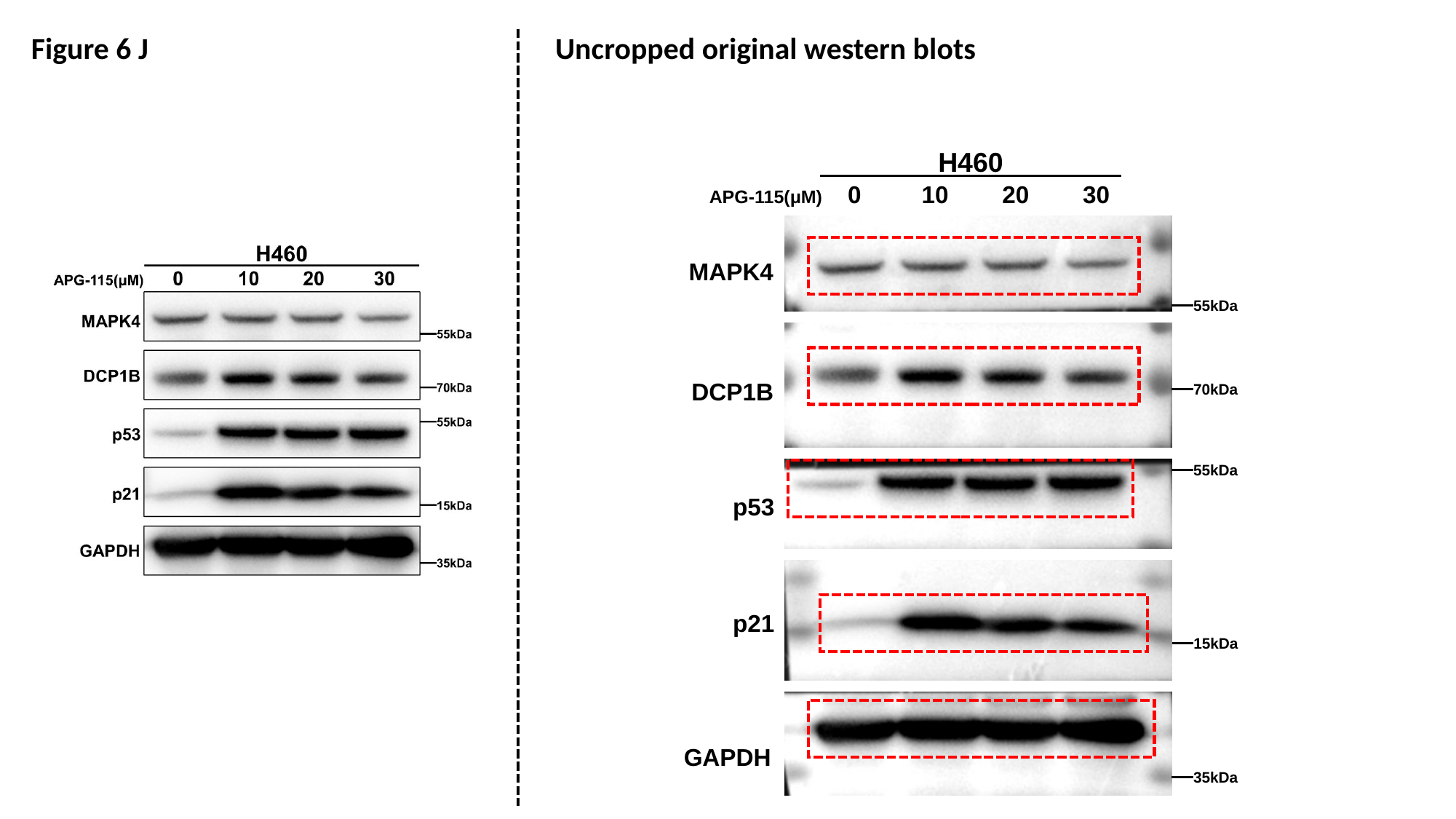

Figure 6 J
Uncropped original western blots
H460
 APG-115(μM) 0 10 20 30
MAPK4
55kDa
DCP1B
70kDa
55kDa
p53
p21
15kDa
GAPDH
35kDa

## Slide 36
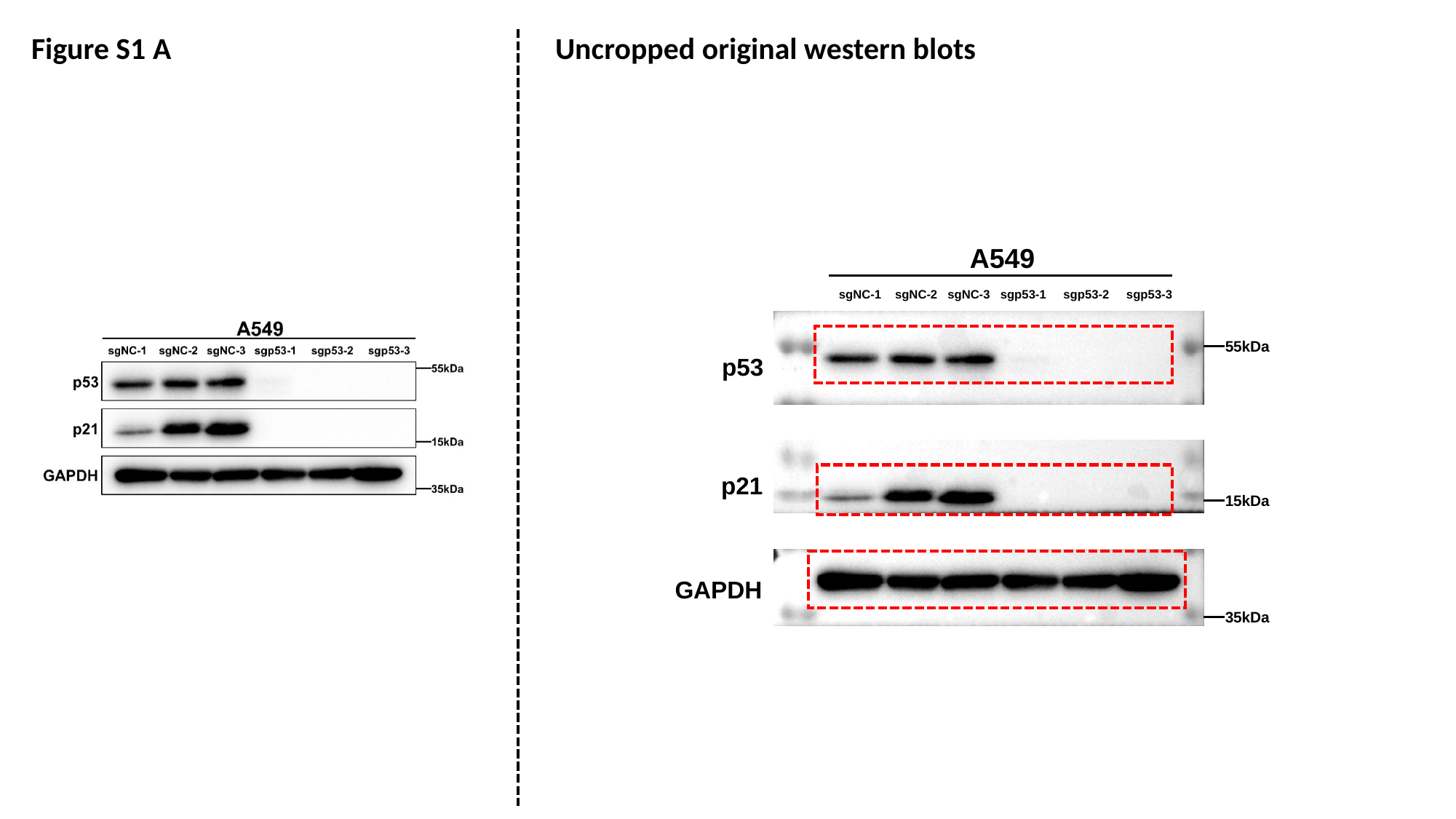

Figure S1 A
Uncropped original western blots
A549
 sgNC-1 sgNC-2 sgNC-3 sgp53-1 sgp53-2 sgp53-3
55kDa
p53
p21
15kDa
GAPDH
35kDa

## Slide 37
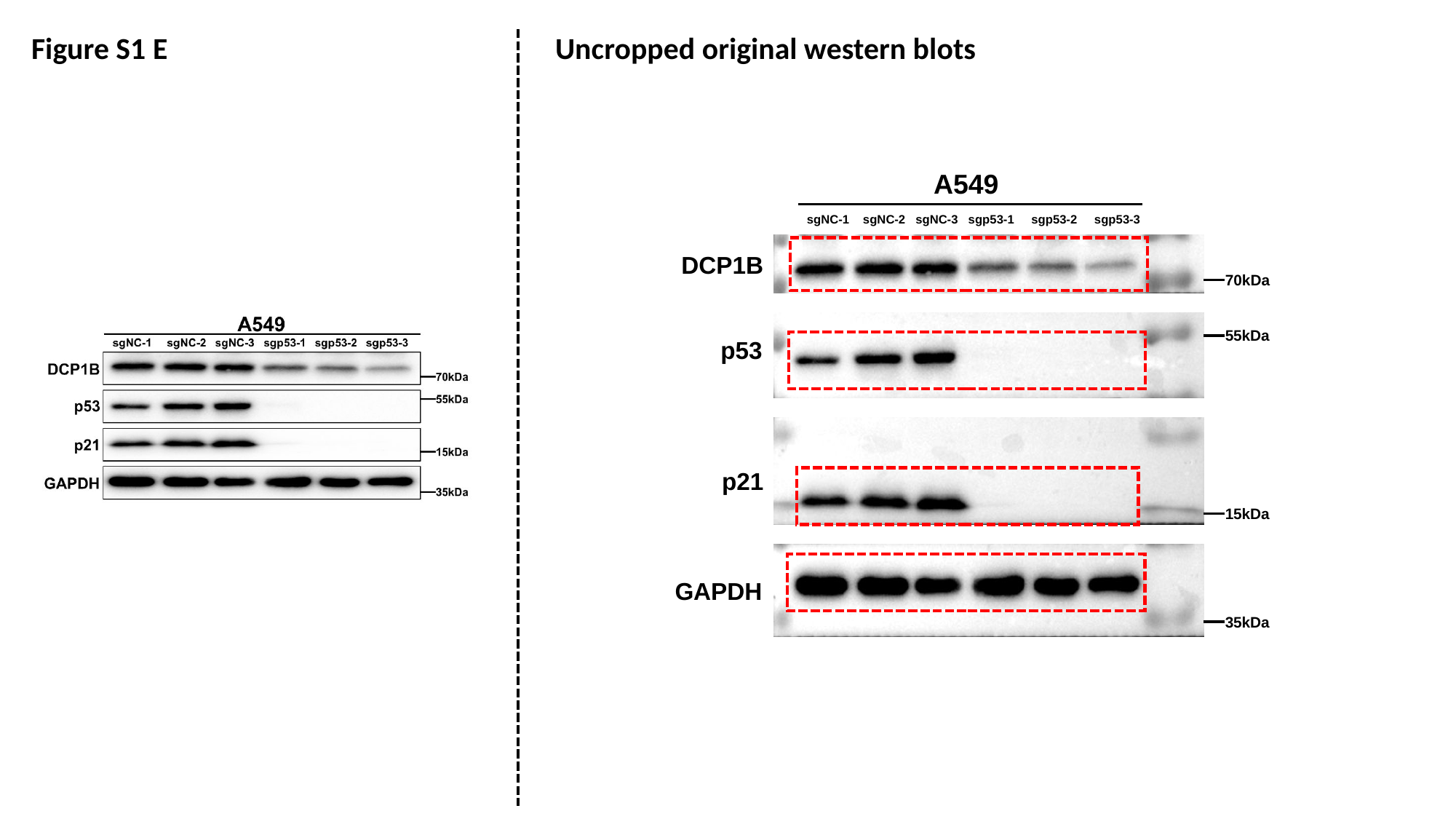

Figure S1 E
Uncropped original western blots
A549
 sgNC-1 sgNC-2 sgNC-3 sgp53-1 sgp53-2 sgp53-3
DCP1B
70kDa
55kDa
p53
p21
15kDa
GAPDH
35kDa

## Slide 38
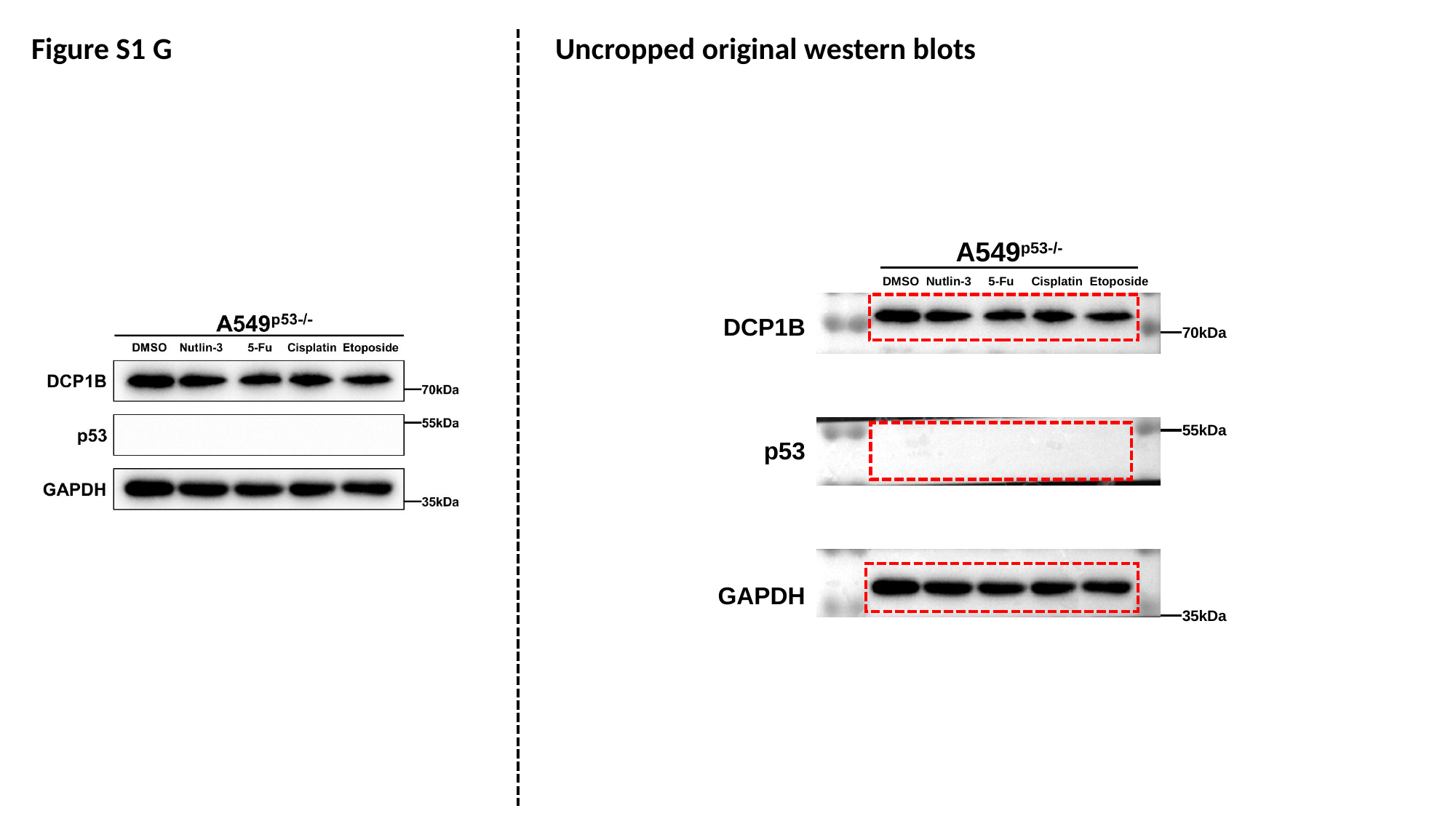

Figure S1 G
Uncropped original western blots
A549p53-/-
DMSO Nutlin-3 5-Fu Cisplatin Etoposide
DCP1B
70kDa
55kDa
p53
GAPDH
35kDa

## Slide 39
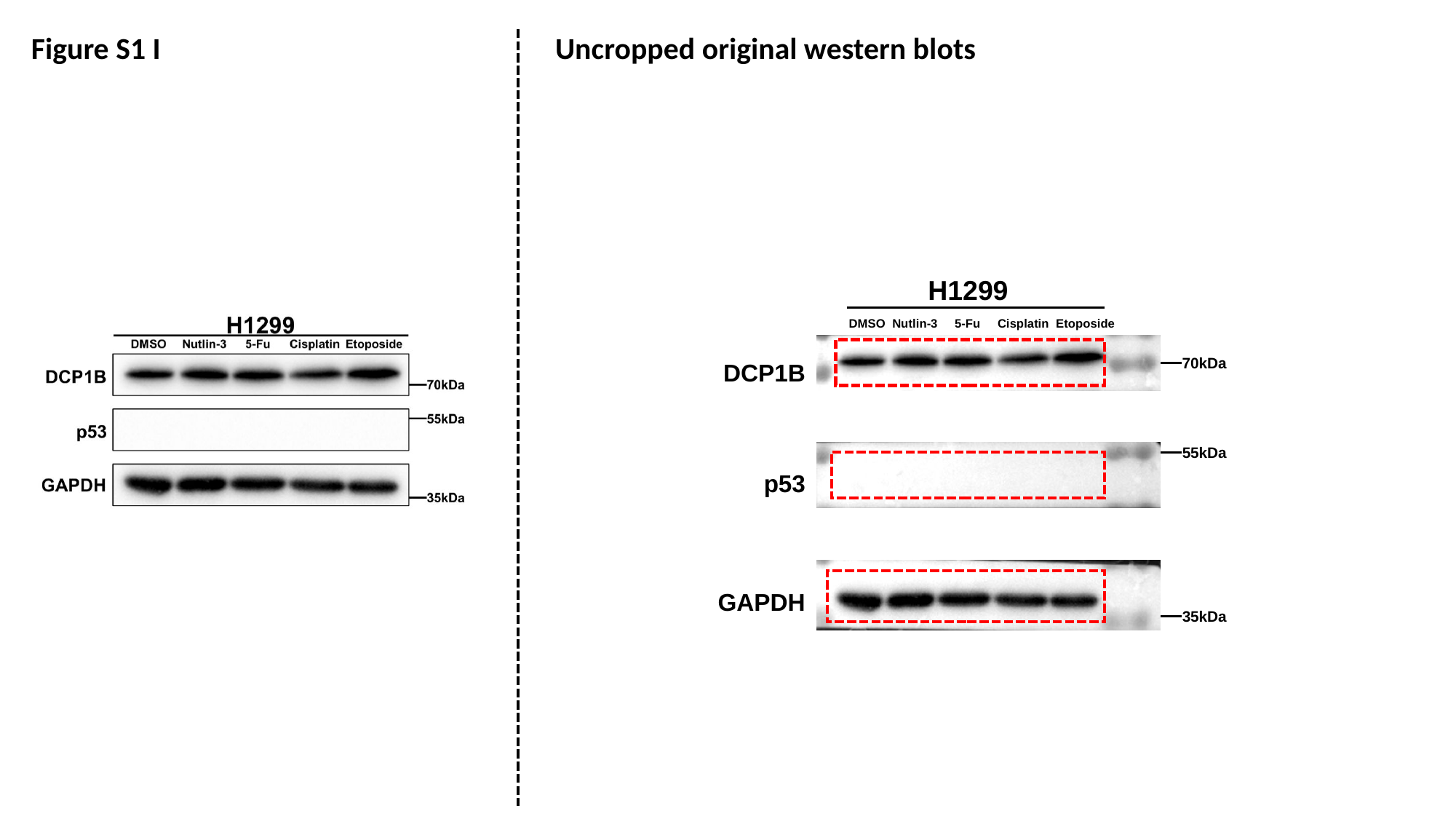

Figure S1 I
Uncropped original western blots
H1299
DMSO Nutlin-3 5-Fu Cisplatin Etoposide
70kDa
DCP1B
55kDa
p53
GAPDH
35kDa

## Slide 40
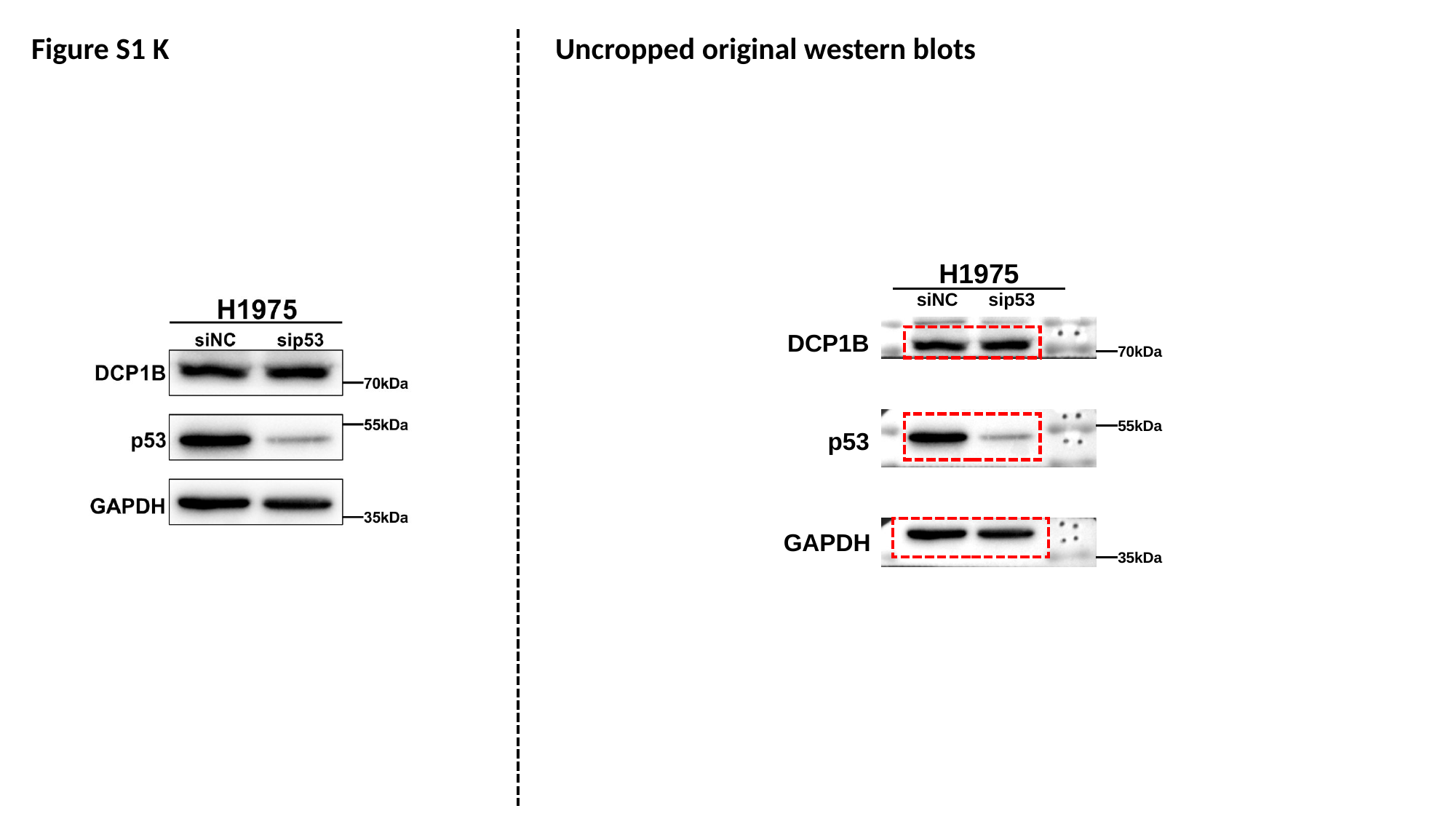

Figure S1 K
Uncropped original western blots
H1975
 siNC sip53
DCP1B
70kDa
55kDa
p53
GAPDH
35kDa

## Slide 41
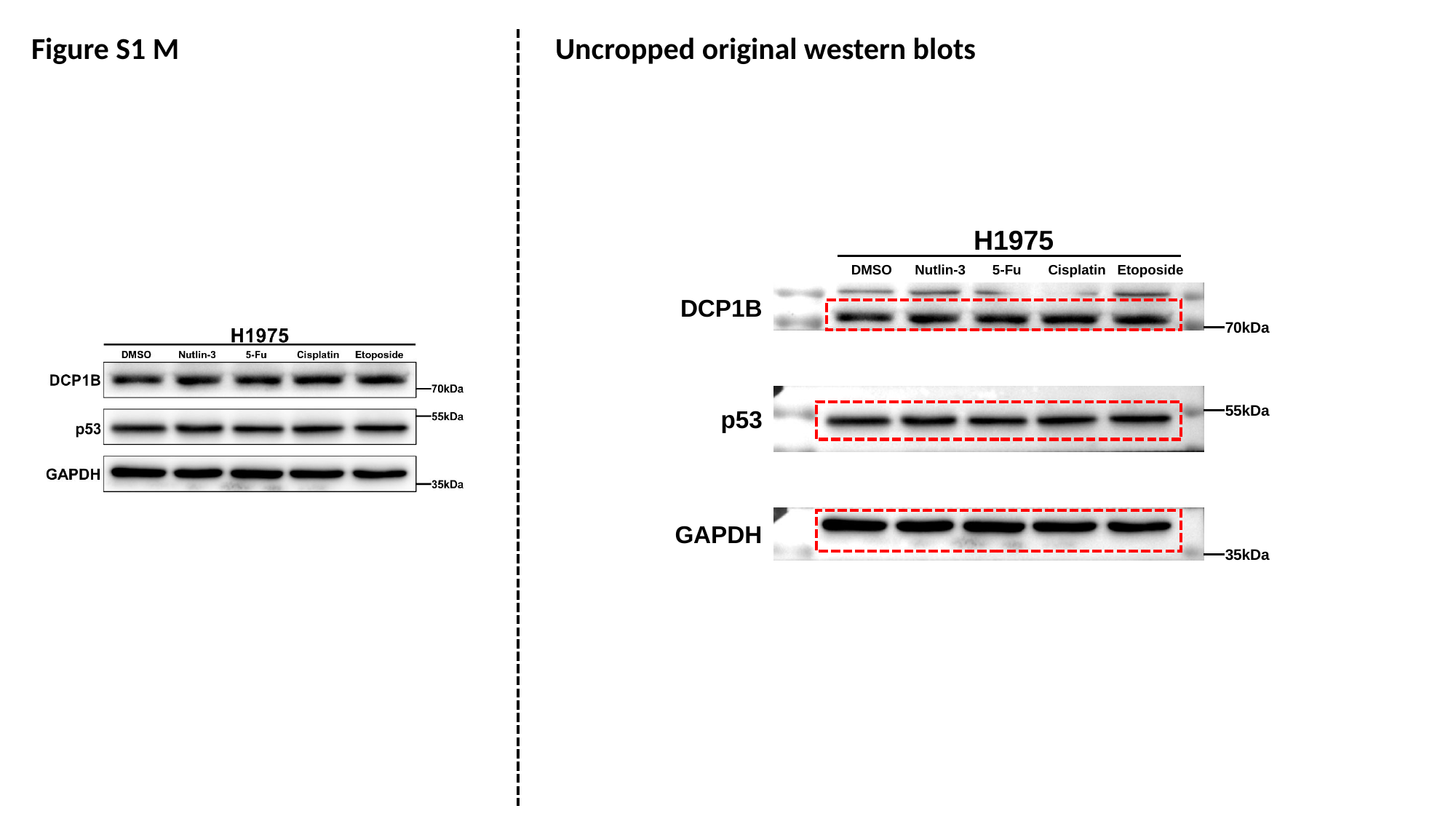

Figure S1 M
Uncropped original western blots
H1975
DMSO Nutlin-3 5-Fu Cisplatin Etoposide
DCP1B
70kDa
55kDa
p53
GAPDH
35kDa

## Slide 42
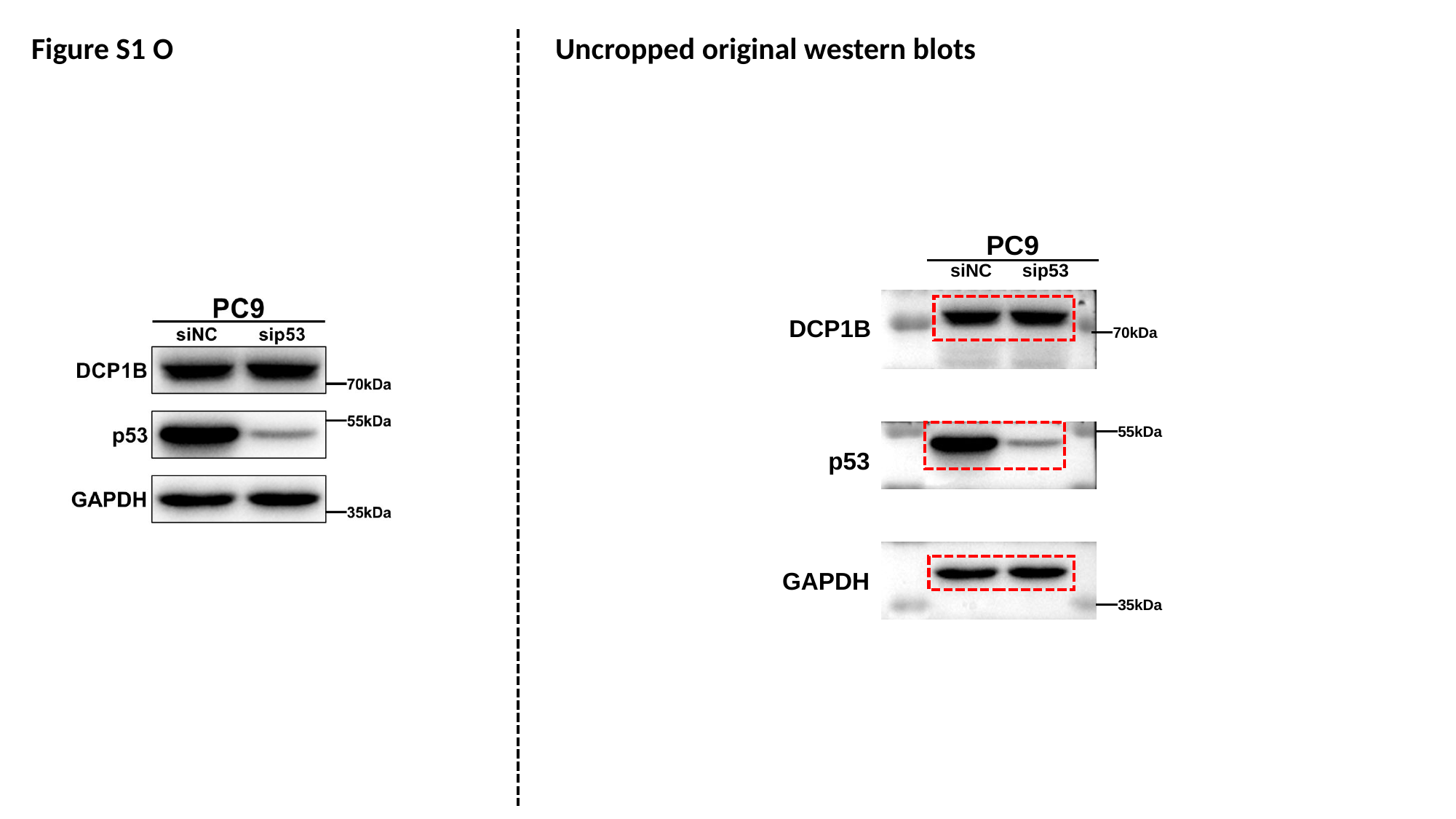

Figure S1 O
Uncropped original western blots
PC9
 siNC sip53
DCP1B
70kDa
55kDa
p53
GAPDH
35kDa

## Slide 43
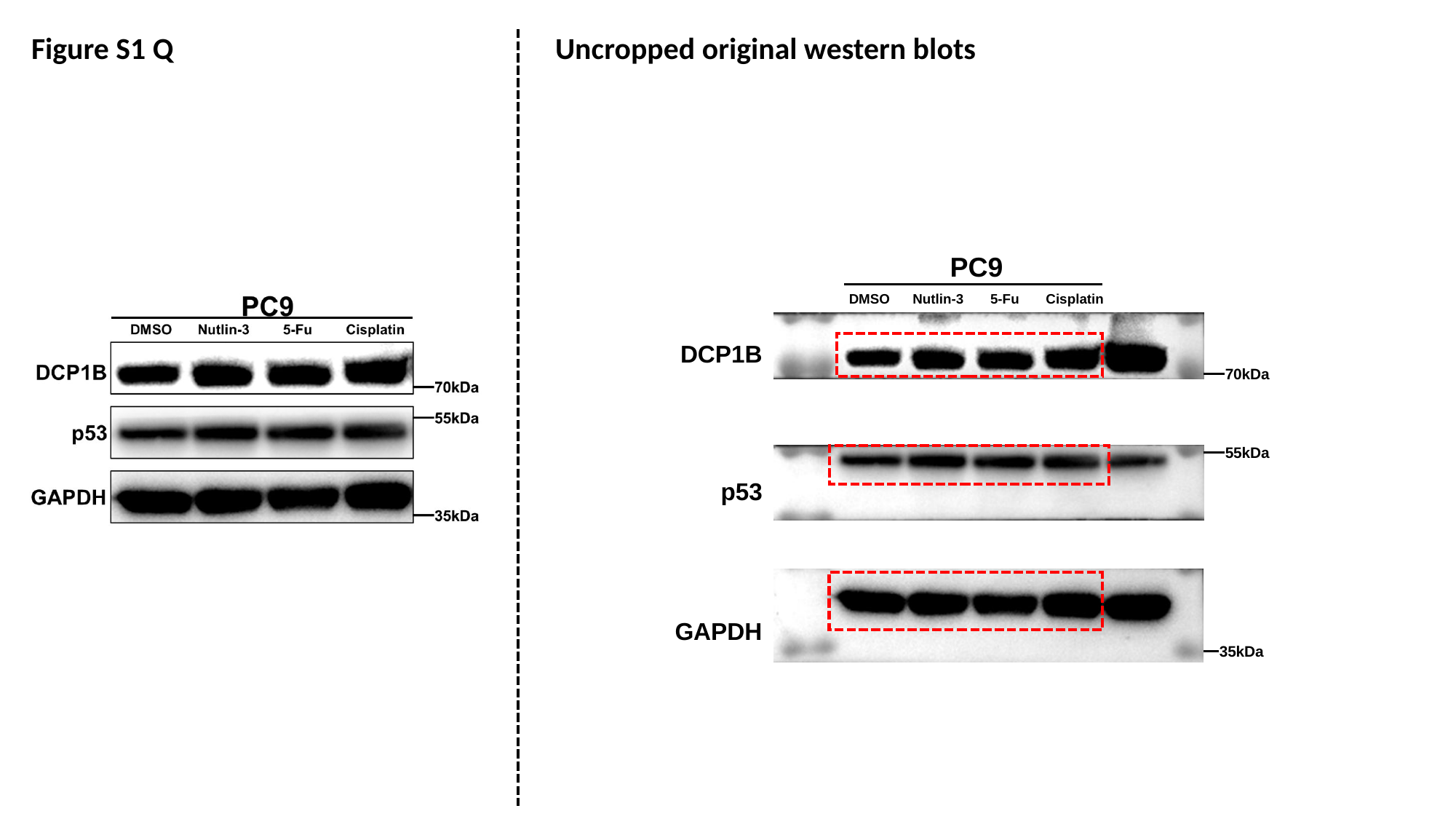

Figure S1 Q
Uncropped original western blots
PC9
DMSO Nutlin-3 5-Fu Cisplatin
DCP1B
70kDa
55kDa
p53
GAPDH
35kDa

## Slide 44
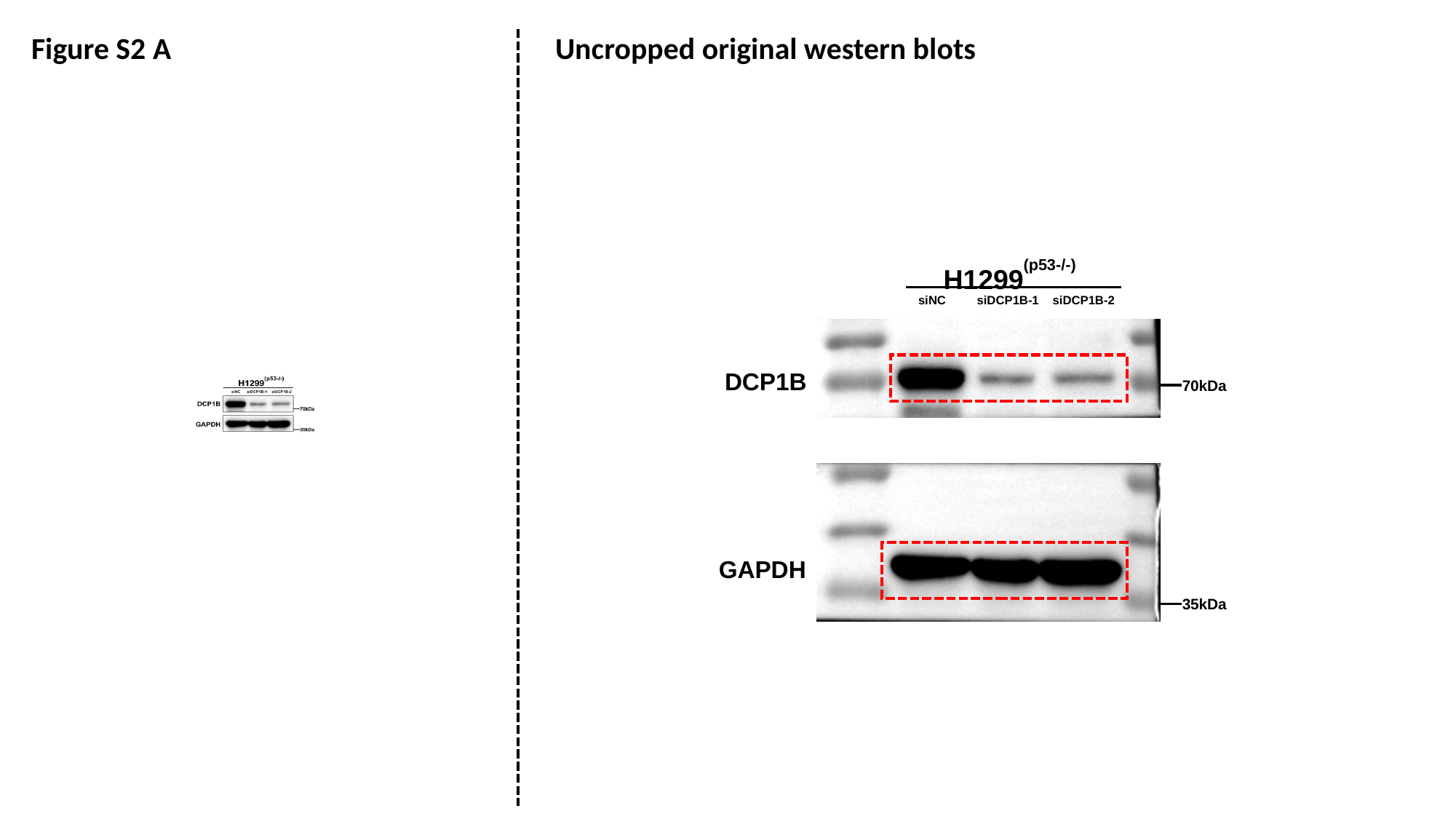

Figure S2 A
Uncropped original western blots
H1299(p53-/-)
 siNC siDCP1B-1 siDCP1B-2
DCP1B
70kDa
GAPDH
35kDa

## Slide 45
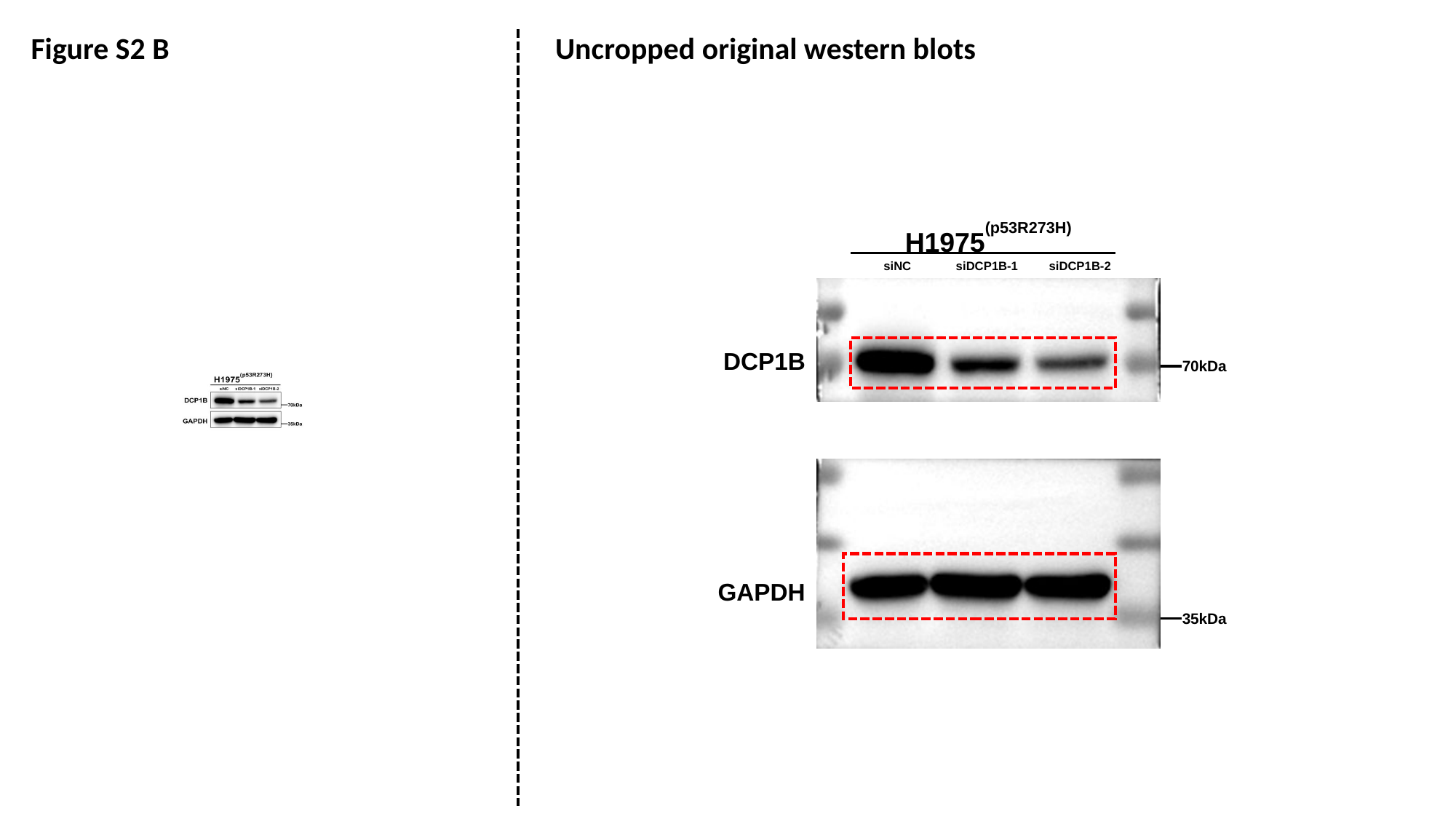

Figure S2 B
Uncropped original western blots
H1975(p53R273H)
 siNC siDCP1B-1 siDCP1B-2
DCP1B
70kDa
GAPDH
35kDa

## Slide 46
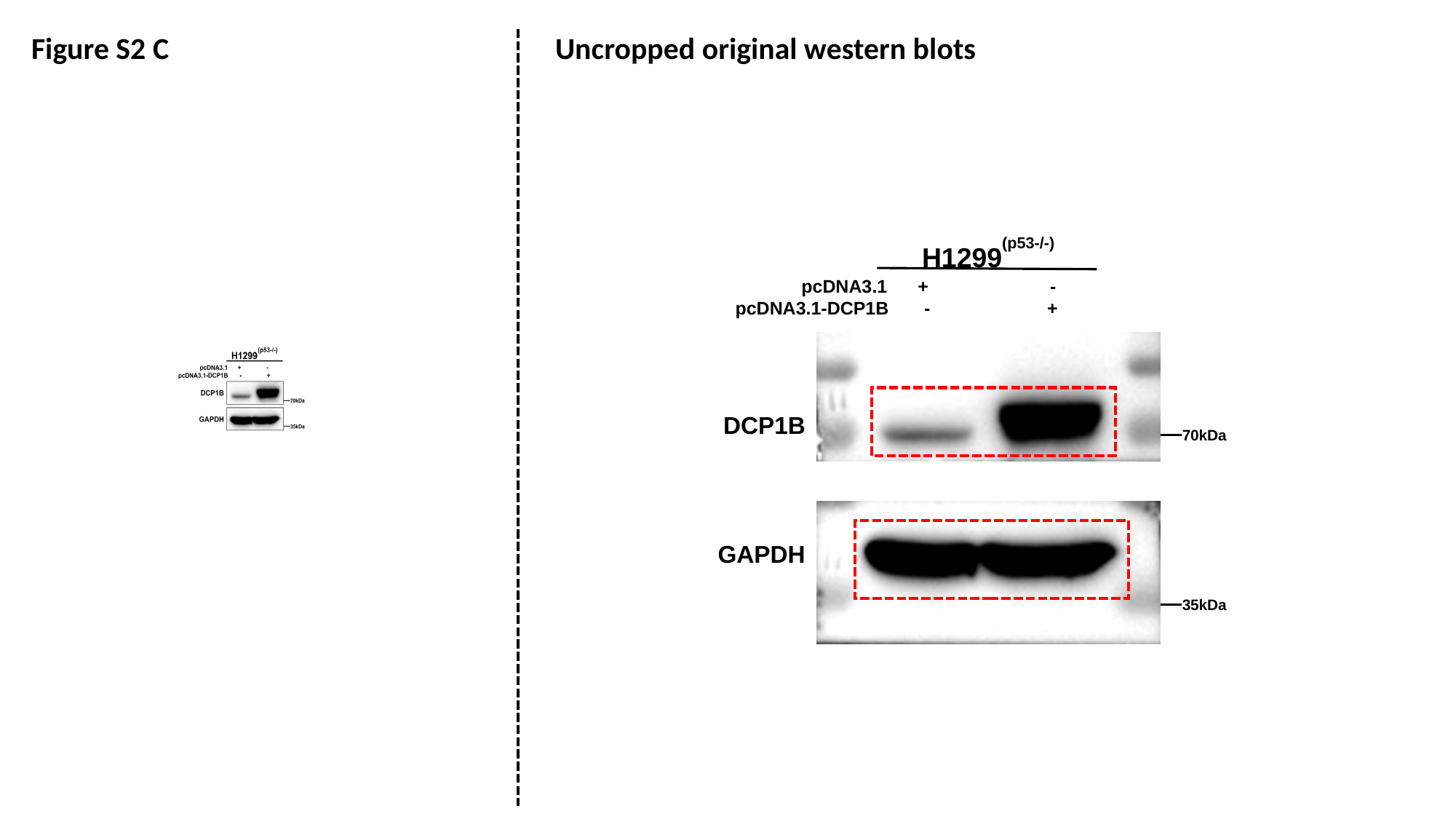

Figure S2 C
Uncropped original western blots
H1299(p53-/-)
 pcDNA3.1 + -
pcDNA3.1-DCP1B - +
DCP1B
70kDa
GAPDH
35kDa

## Slide 47
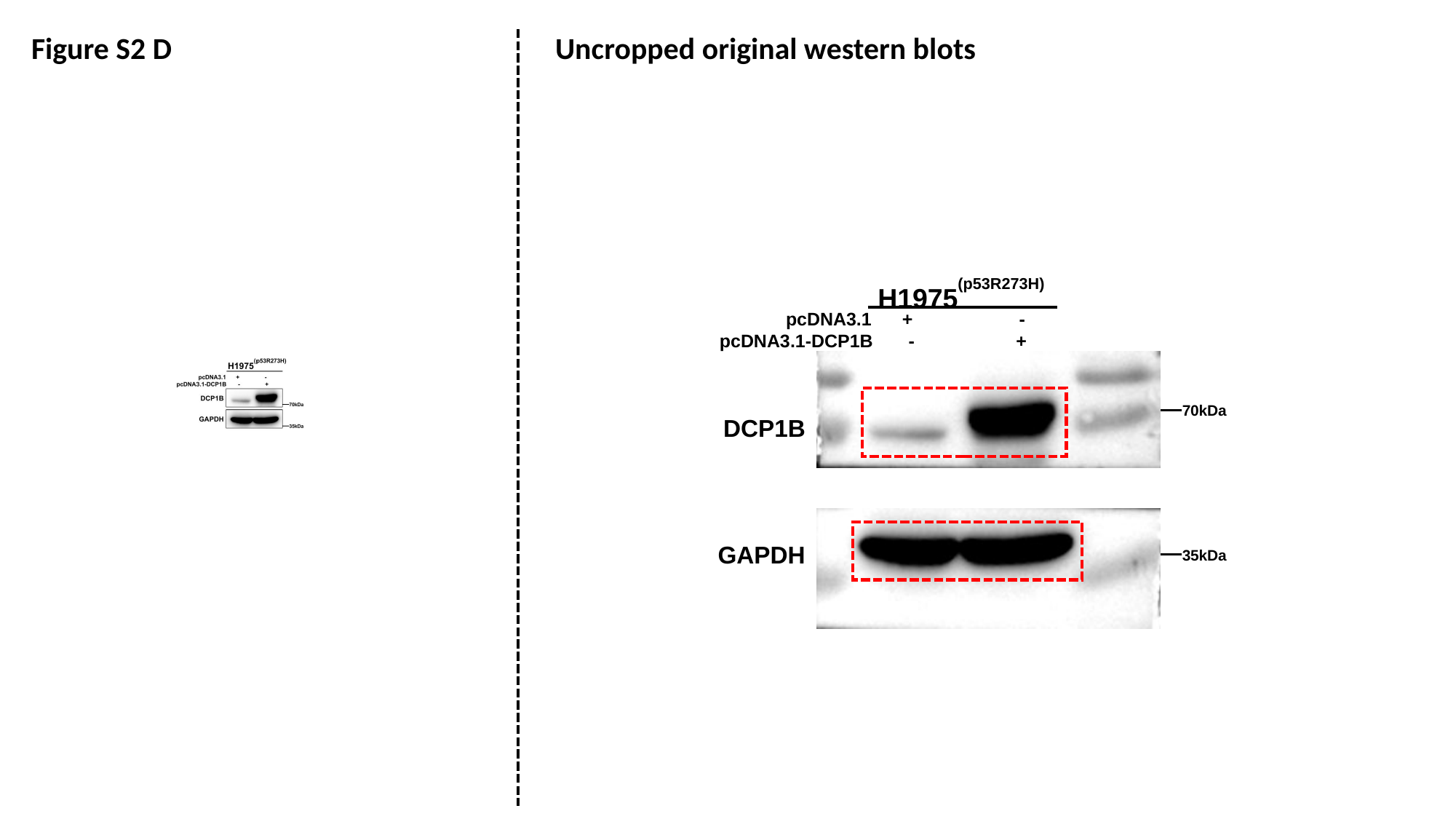

Figure S2 D
Uncropped original western blots
H1975(p53R273H)
 pcDNA3.1 + -
pcDNA3.1-DCP1B - +
70kDa
DCP1B
GAPDH
35kDa

## Slide 48
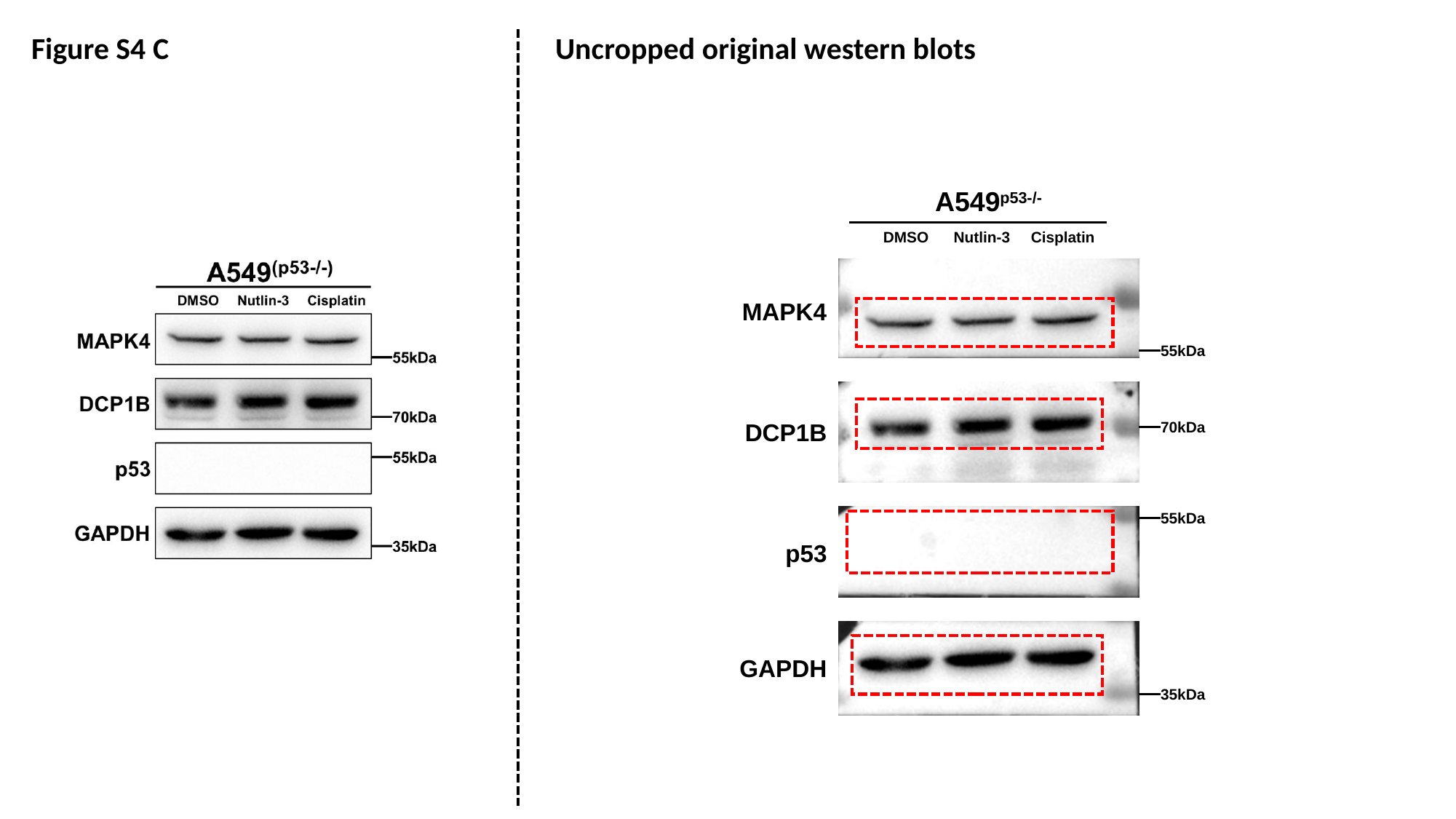

Figure S4 C
Uncropped original western blots
A549p53-/-
DMSO Nutlin-3 Cisplatin
MAPK4
55kDa
DCP1B
70kDa
55kDa
p53
GAPDH
35kDa

## Slide 49
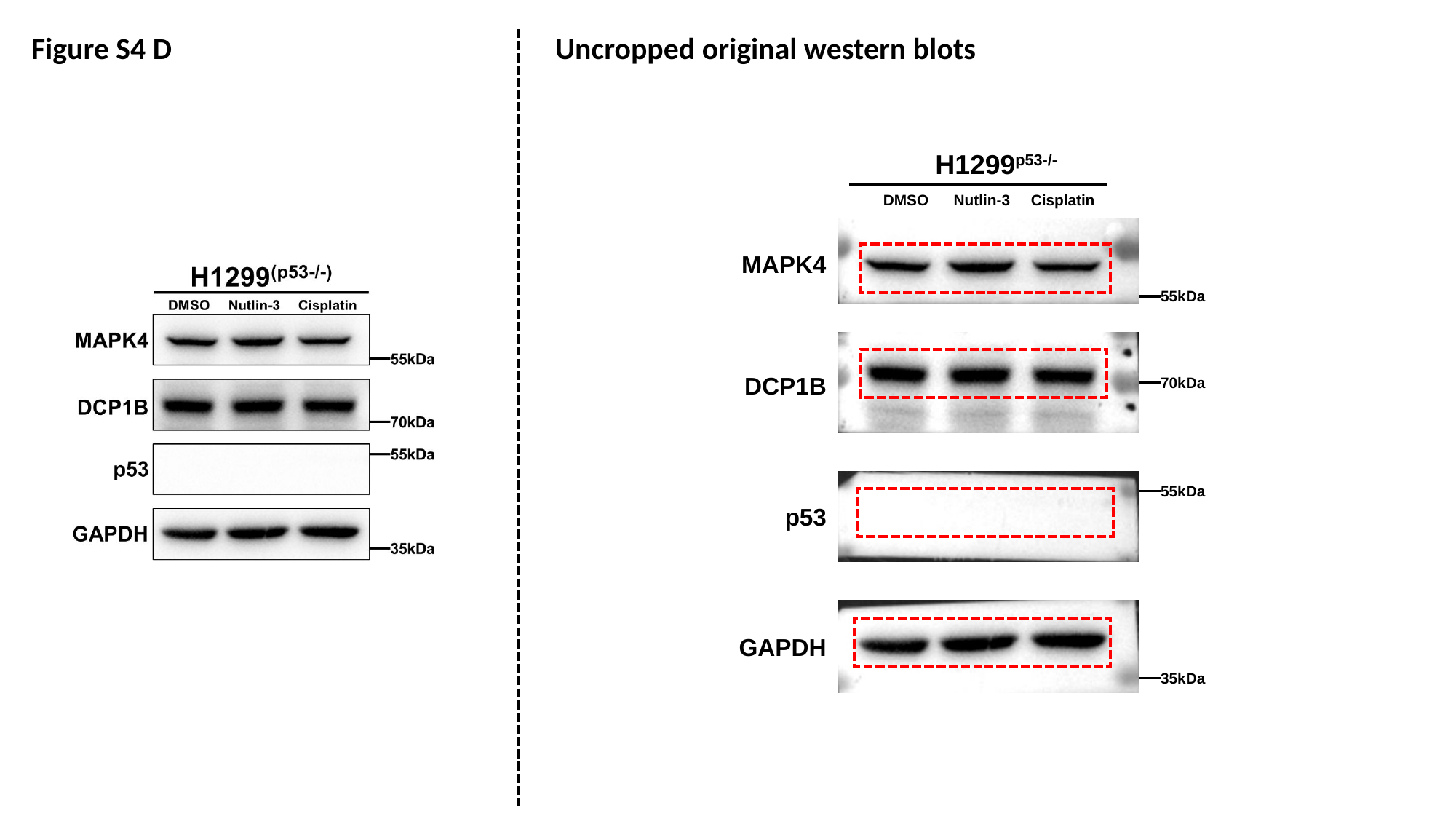

Figure S4 D
Uncropped original western blots
H1299p53-/-
DMSO Nutlin-3 Cisplatin
MAPK4
55kDa
DCP1B
70kDa
55kDa
p53
GAPDH
35kDa

## Slide 50
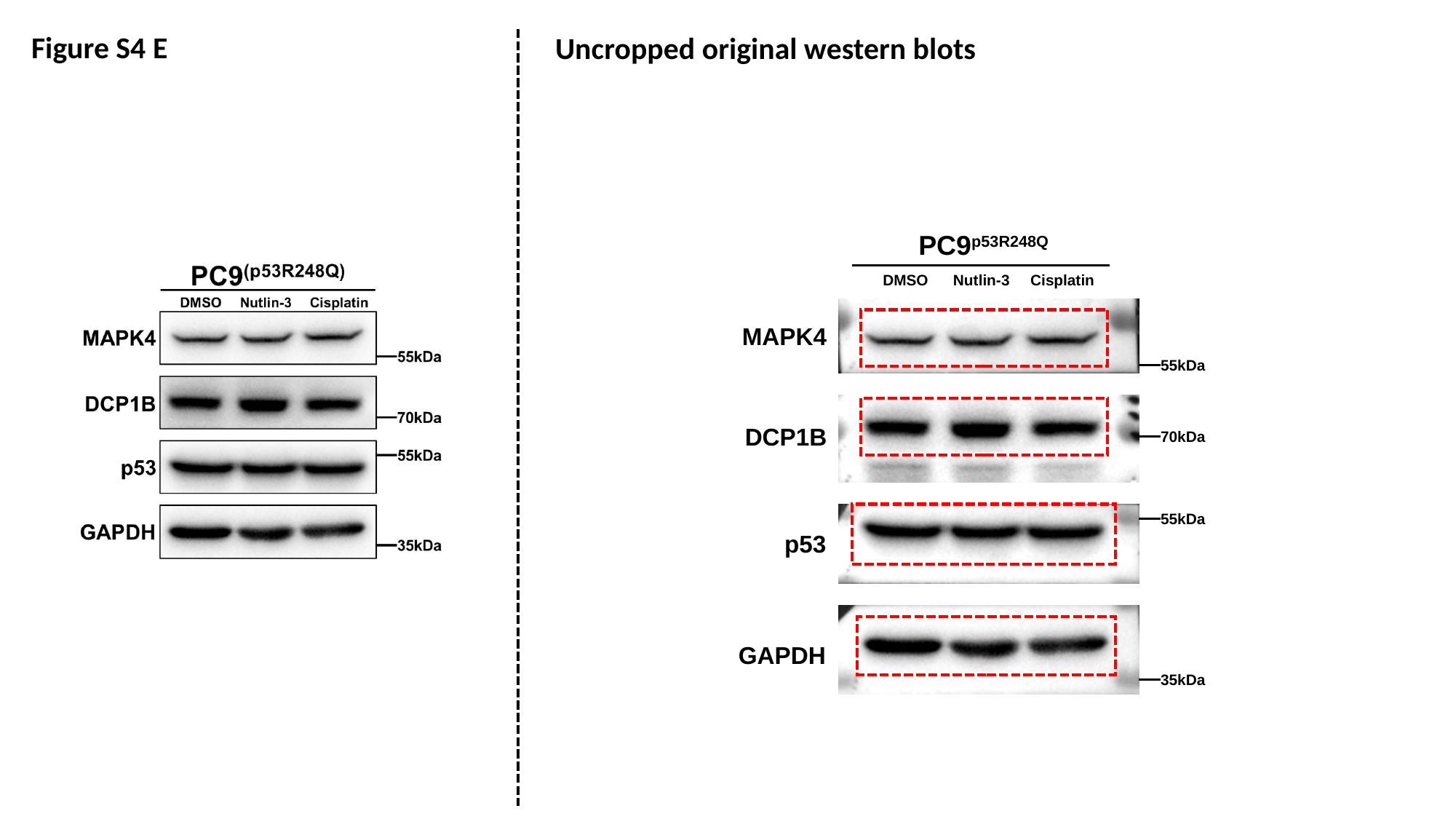

Figure S4 E
Uncropped original western blots
PC9p53R248Q
DMSO Nutlin-3 Cisplatin
MAPK4
55kDa
DCP1B
70kDa
55kDa
p53
GAPDH
35kDa

## Slide 51
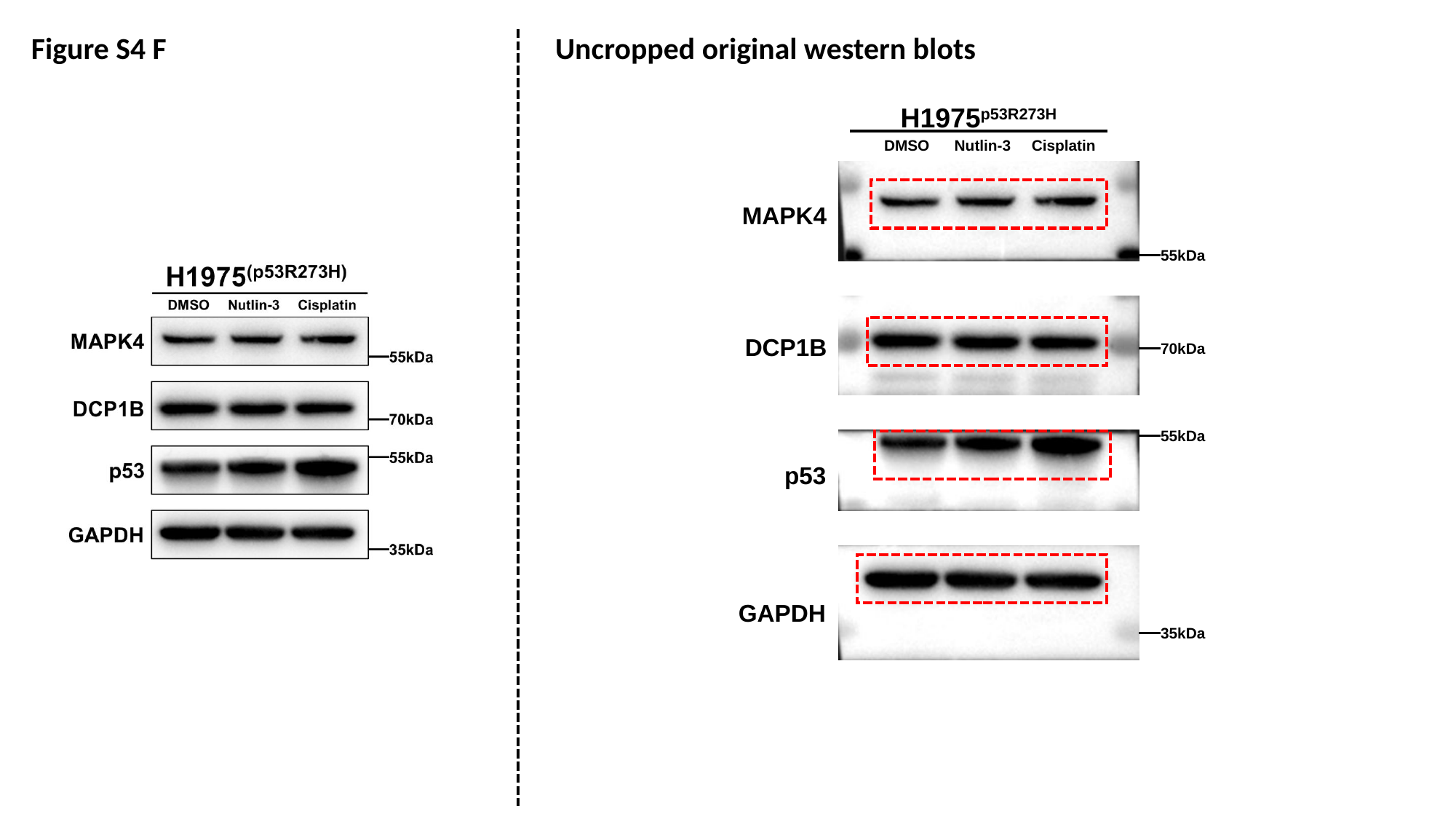

Figure S4 F
Uncropped original western blots
H1975p53R273H
DMSO Nutlin-3 Cisplatin
MAPK4
55kDa
DCP1B
70kDa
55kDa
p53
GAPDH
35kDa

## Slide 52
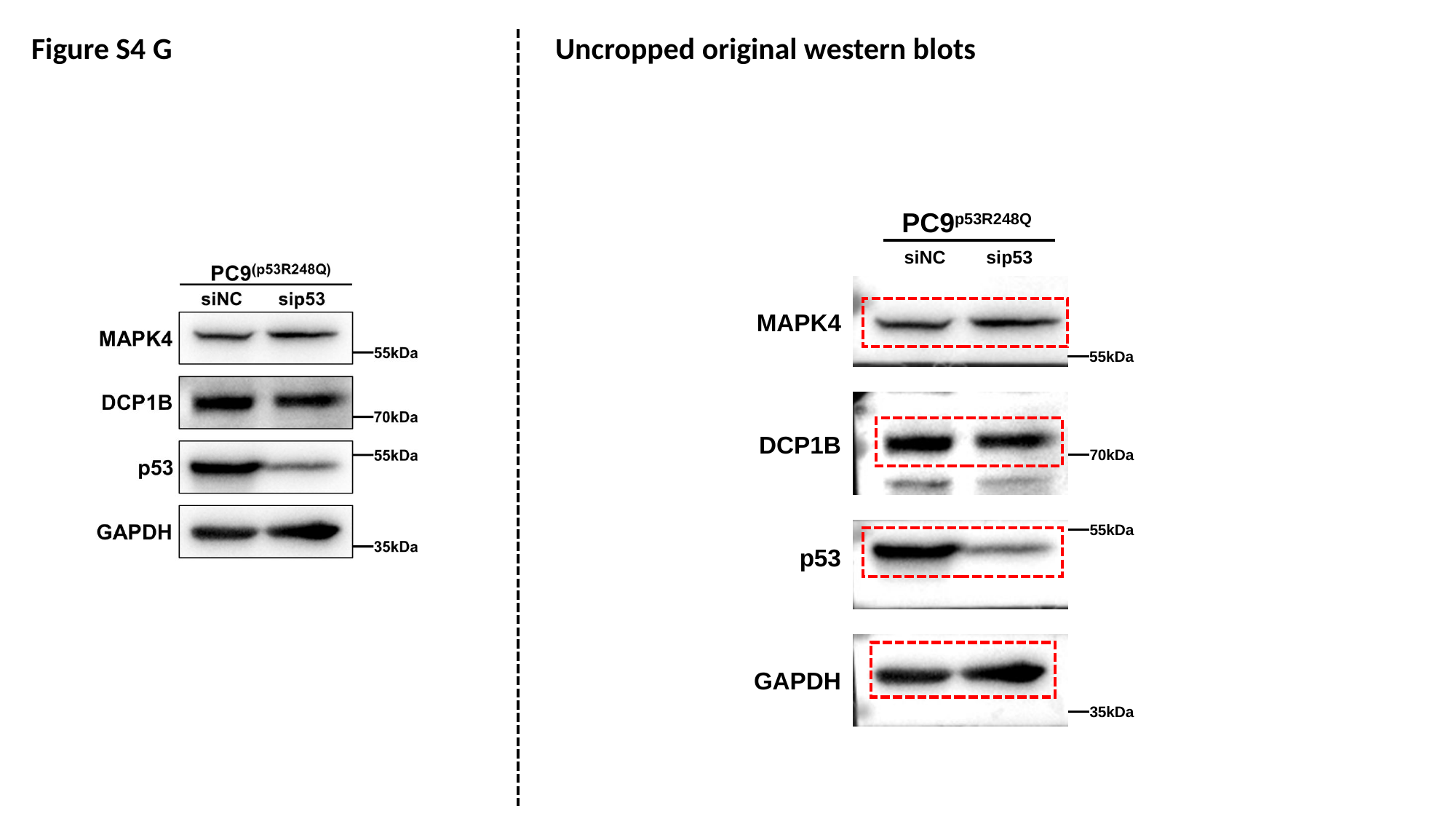

Figure S4 G
Uncropped original western blots
PC9p53R248Q
 siNC sip53
MAPK4
55kDa
DCP1B
70kDa
55kDa
p53
GAPDH
35kDa

## Slide 53
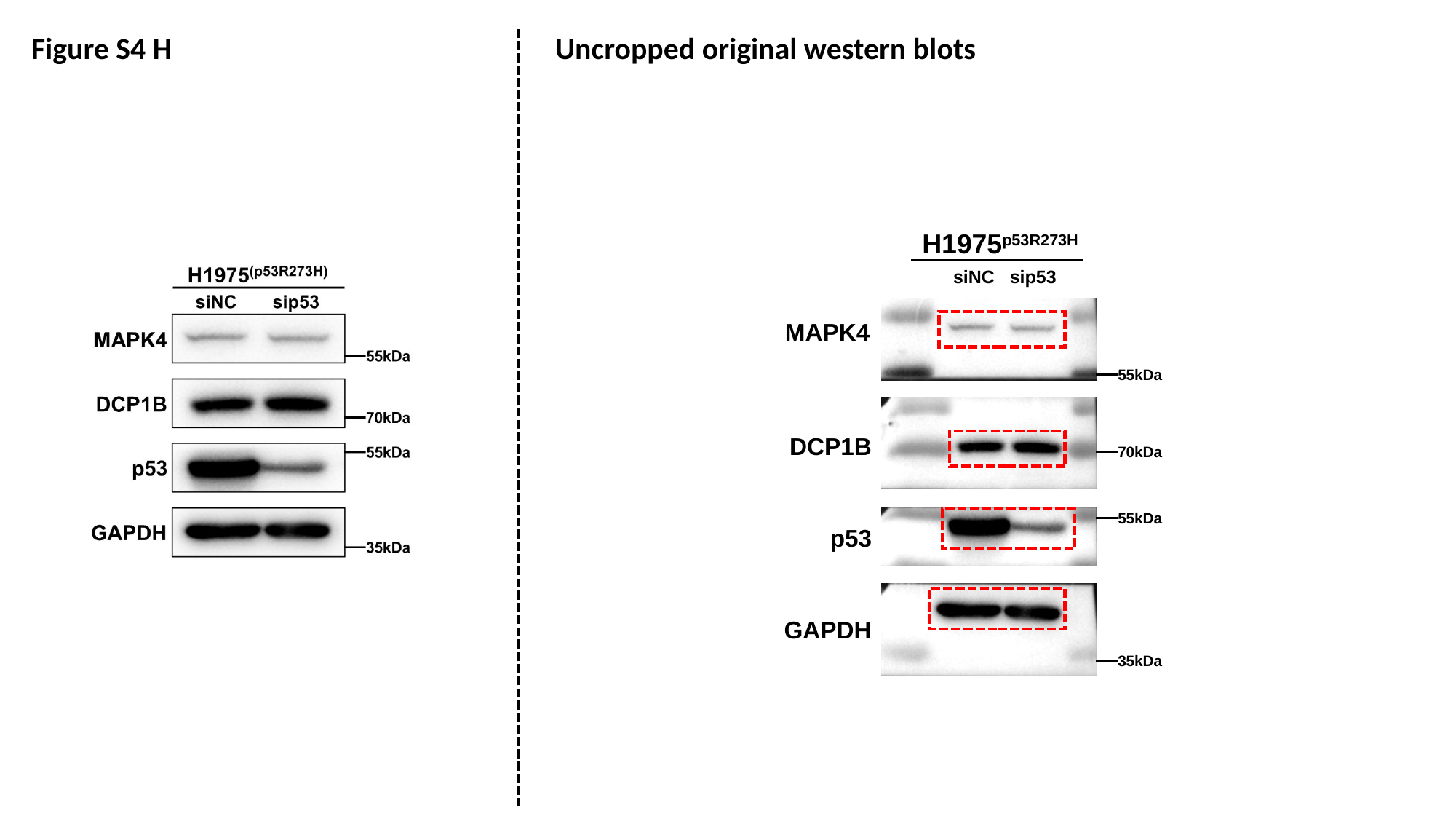

Figure S4 H
Uncropped original western blots
H1975p53R273H
 siNC sip53
MAPK4
55kDa
DCP1B
70kDa
55kDa
p53
GAPDH
35kDa

## Slide 54
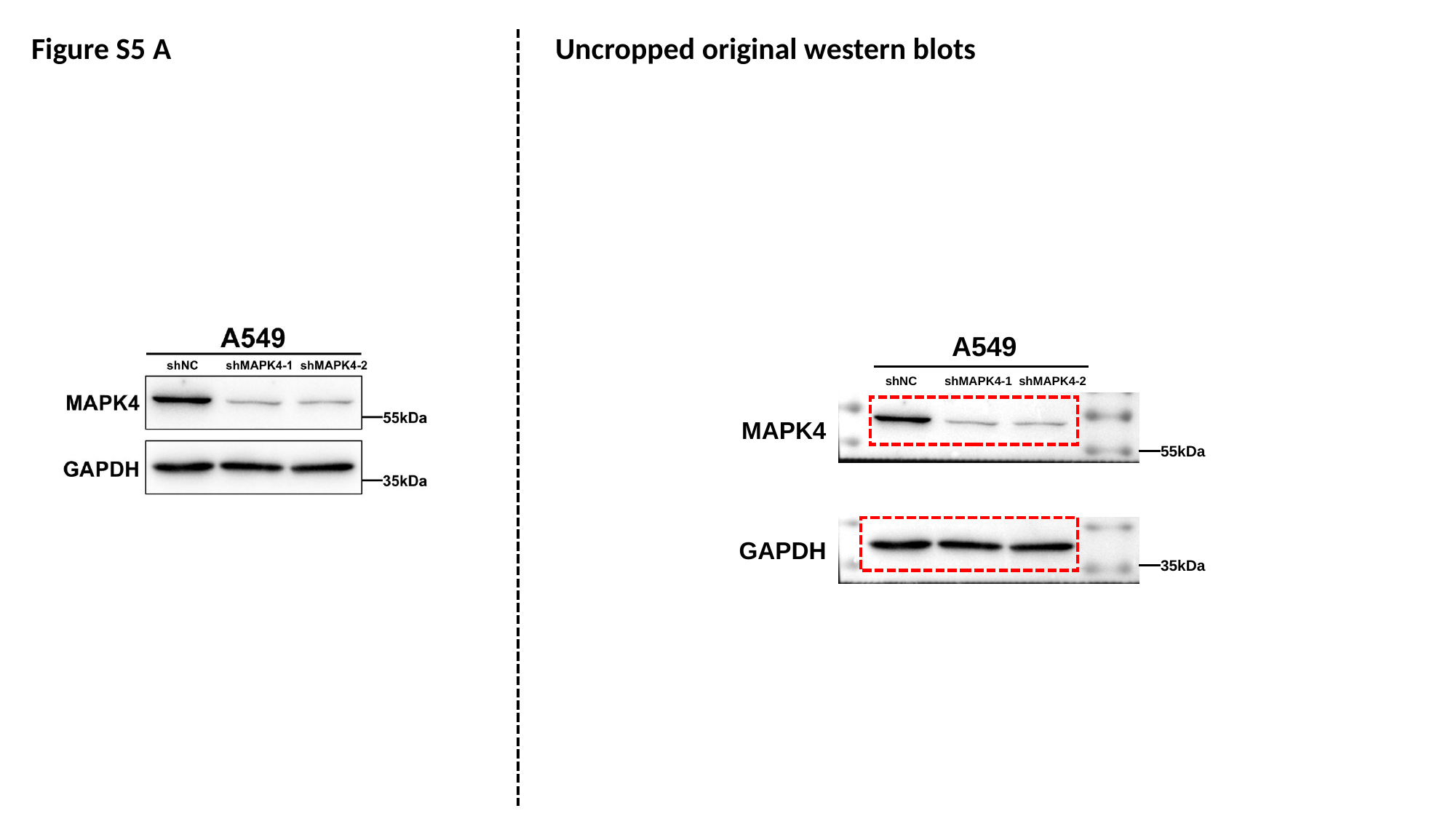

Figure S5 A
Uncropped original western blots
A549
 shNC shMAPK4-1 shMAPK4-2
MAPK4
55kDa
GAPDH
35kDa

## Slide 55
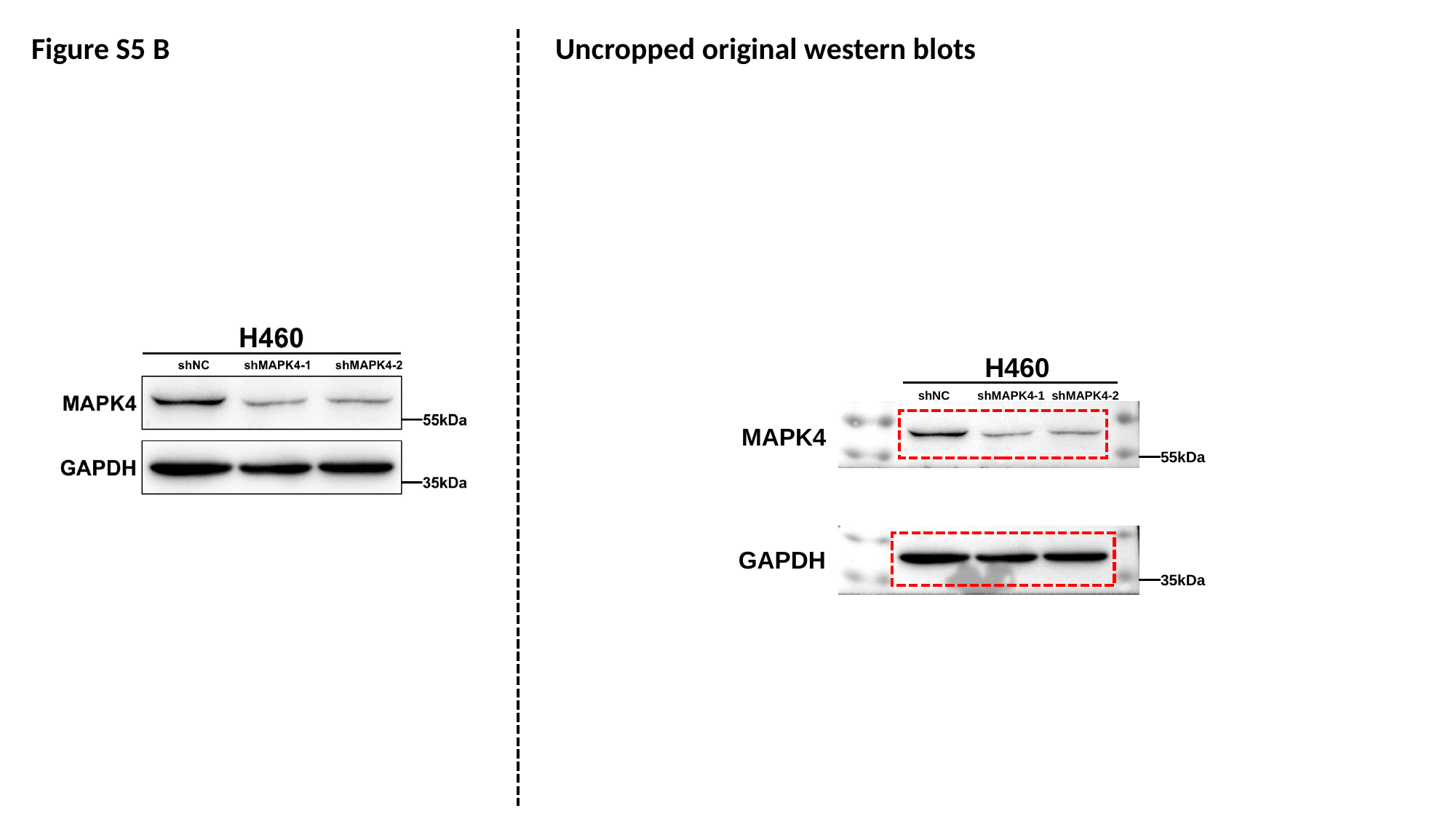

Figure S5 B
Uncropped original western blots
H460
 shNC shMAPK4-1 shMAPK4-2
MAPK4
55kDa
GAPDH
35kDa

## Slide 56
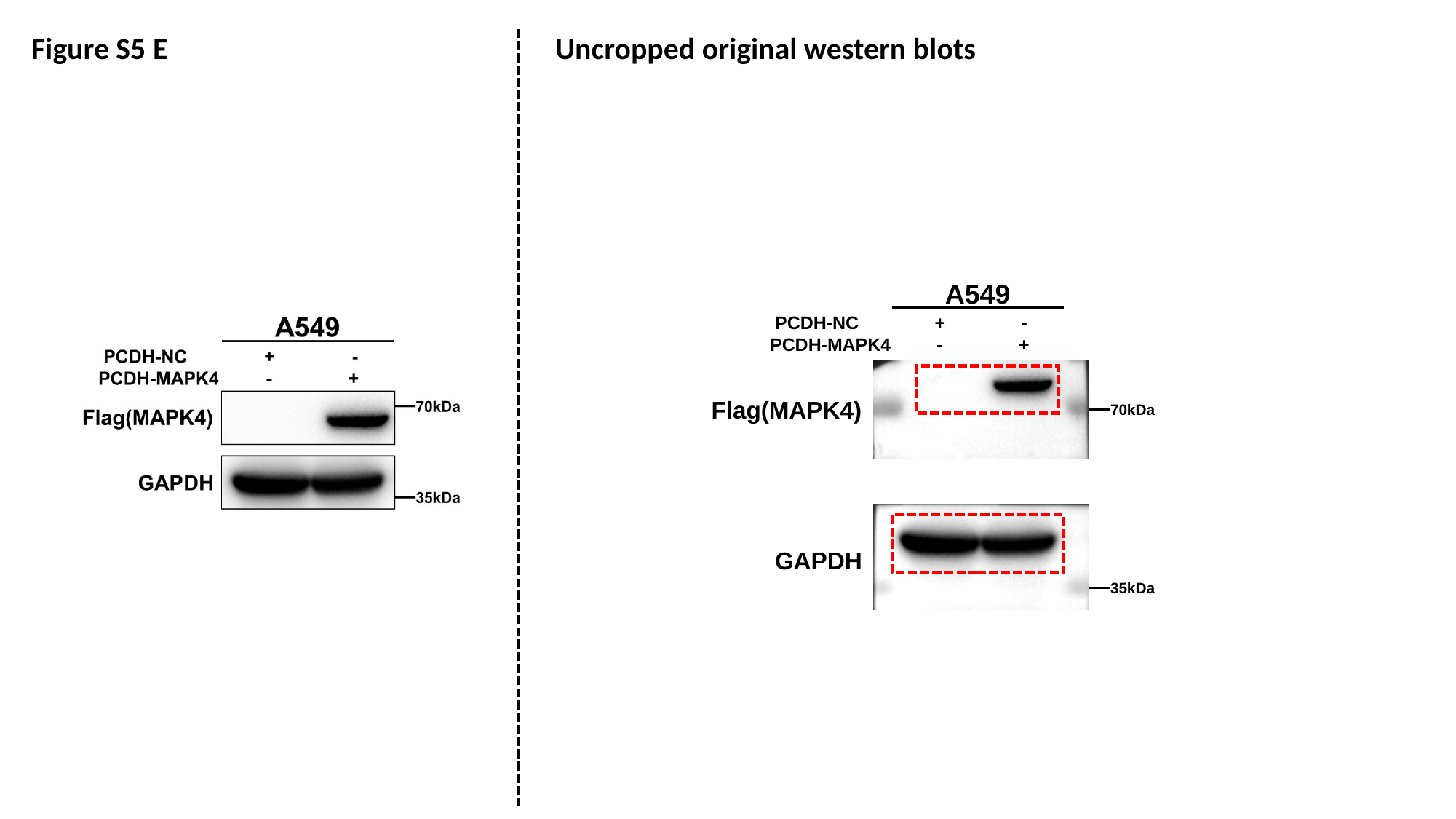

Figure S5 E
Uncropped original western blots
A549
 PCDH-NC + -
PCDH-MAPK4 - +
Flag(MAPK4)
70kDa
GAPDH
35kDa

## Slide 57
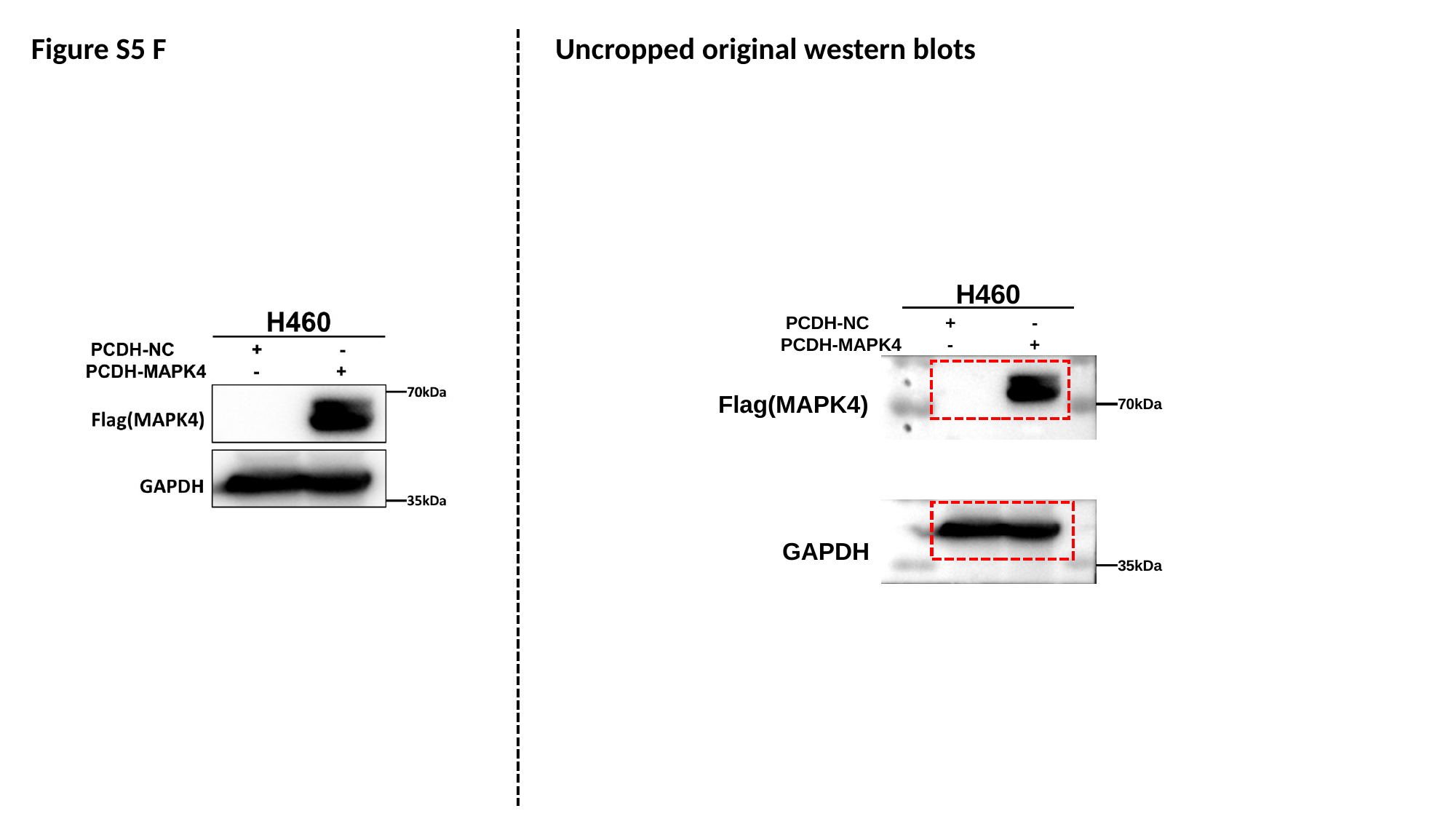

Figure S5 F
Uncropped original western blots
H460
 PCDH-NC + -
PCDH-MAPK4 - +
Flag(MAPK4)
70kDa
GAPDH
35kDa
